# Medical equipment in the global south: perspective of sustainability and donations

**DOI:** 10.3389/frhs.2025.1638305

**Published:** 2025-09-04

**Authors:** Frederik Paustian, Rasmus Gøl, Hannah Wolfe Julsgart, Sofie Bjerre Degn, Andreas Philip Rosenbom, Anton Aaby Henriksen, Liselotte Højgaard

**Affiliations:** ^1^ Department of Health Technology, Technical University of Denmark, Kgs. Lyngby, Denmark; ^2^ Department of Clinical Physiology and Nuclear Medicine, University of Copenhagen and Rigshospitalet, Copenhagen, Denmark

**Keywords:** medical equipment sustainability, healthcare and technology in developing countries, hospital infrastructure, medical equipment failure, medical equipment longevity, medical equipment donations

## Abstract

Disparities in healthcare infrastructure between the Global South and North continue to affect medical equipment availability, functionality, and sustainability in low- and middle-income countries (LMICs). This study combines a systematic literature review with on-site fieldwork in Ugandan hospitals to assess the current state of medical equipment in LMICs and propose actionable strategies for more sustainable donation practices. Following a systematic literature review, 18 articles were analyzed and categorized according to five research questions addressing sustainability metrics, affordability, recycling practices, systemic barriers, and innovations in medical equipment use. Parallel fieldwork conducted by biomedical engineering volunteers in two Ugandan hospitals documented over 1,400 devices and resulted in the repair of 51 items—generating estimated savings of $102,000. Many devices remained unused due to a lack of spare parts and contextual compatibility. A carbon footprint assessment of donated equipment shipment from Denmark to Uganda further underscored the environmental implications of donation programs. Drawing on literature insights and field observations, this paper proposes a set of eight principles to enhance the sustainability and long-term impact of medical equipment donations. Emphasizing context-aware design, training, maintenance, and donor-recipient collaboration, these recommendations aim to shift donation models toward more resilient and responsible healthcare partnerships.

## 1 Introduction

The disparity and gap between the quality of hospitals in the Global South and the Global North cause significant negative consequences regarding the healthcare delivery opportunities (1). However, healthcare in the Global South has improved significantly in the past 10–20 years, reflecting global progress. This development has enabled local hospitals to provide more efficient diagnosis and treatment. Despite ongoing challenges and inequalities in both the Global South and North, many regions have seen progress in health, education, and living standards, including lower child mortality, longer life expectancy, reduced poverty, and advances in medical care and technology (2). Nevertheless, hospitals in the Global South still lack robust and well-functioning medical equipment, with vast amounts of unused equipment taking up much space. Between 40%–70% of the medical equipment in hospitals in Low- and Middle-Income Countries (LMICs) is out of order.^1^

The equipment may be put away due to a lack of knowledge on how to repair and maintain it, and may even be unusable in the environment. Hospitals in LMICs rarely have the resources to provide the repair services needed. This is an immediate risk in the donation of used equipment from advanced hospitals to low-resource recipient settings.

Some of these issues were experienced firsthand during the Engineering World Health’s (EWH) Summer Institute 2024 in Uganda and Guatemala. EWH (now merged with Engineers Without Borders, EWB) is a non-profit organization that strives to inspire, educate, and empower the biomedical engineering community to improve healthcare delivery around the world. This is done, among other things, by facilitating “Summer Institutes for Volunteers.” These consist of training in repairs of medical equipment in low-resource hospitals, followed by volunteer work at hospitals for five weeks, repairing medical equipment.

In 2023, approximately 50 EWH volunteers completed 497 medical equipment repairs globally, saving LMICs an estimated $934,000. Approximately 70% of the equipment was restored to use, reducing waste and supporting both healthcare delivery and environmental sustainability.^1^

The Technical University of Denmark (DTU) has established a local branch of EWH, which has been running for nearly 15 years. In the summer of 2024, a group of 10 Biomedical Engineering master’s students from DTU were in Uganda and Guatemala on the EWH Summer Institute, working pro bono for the summer.

The purpose of this paper is to provide a brief report on our findings regarding medical equipment in a group of LMIC hospitals, supplemented by a systematic literature review, and a sustainability assessment of a medical equipment donation. This forms the basis for our call to action for more sustainable solutions regarding the interplay of the Global North and South regarding medical equipment.

## 2 Methods

### 2.1 Literature review

A systematic literature review was conducted to evaluate the current state of sustainability in relation to the use, maintenance, and procurement of medical devices in the Global South. The search followed the PRISMA guidelines (3) using the PICO method to build the search strings. The articles have then been screened on abstract levels for relevance regarding the research questions presented in Table 1. The ones deemed relevant have been scored in a full-text appraisal screening, by two individual readers.

**Table 1:** Research questions for sustainability in medical technologies.

No.	Research questions
1	Key metrics for assessing environmental sustainability in the design, implementation, and maintenance of medical technologies in low- and middle-income countries (LMICs).
2	Affordability challenges in the deployment of sustainable medical devices while ensuring equitable healthcare access in LMICs.
3	Recycling and reuse strategies for improving the environmental and economic sustainability of medical devices in low-resource settings.
4	Social and economic barriers in the adoption and maintenance of sustainable medical technologies in LMICs.
5	Innovations in sustainable medical device design

Searches were conducted using the research questions (Table 1) in the PubMed and EMBASE databases. The search strings used are found in Supplementary Material S1.

### 2.2 Equipment observations: inventory assessment

Engineering World Health (EWH) organized a volunteer initiative in the summer of 2024, sending biomedical engineering student volunteers to the Global South. Among these, four individuals were deployed to Uganda, working pro bono in local hospitals in two cities, Mbarara and Masaka. Their efforts were concentrated in the local hospitals: St. Joseph’s Hospital Kitovu in Masaka and Mbarara Regional Referral Hospital. Medical equipment inventory lists were obtained for both hospitals. At one hospital, a digital inventory list was provided by the hospital director. This list was detailed and comprehensive, including information such as manufacturer details, model and serial numbers, and the precise location of each item within the facility. Conversely, at the other hospital, no electronic inventory existed. To address this, the volunteers conducted a thorough manual inventory, meticulously cataloging equipment by visiting every ward, office, and storage area.

Equipment was categorized into predefined groups based on function (e.g., diagnostic, surgical, imaging), and used for analysis and comparison.

**Figure 1:**
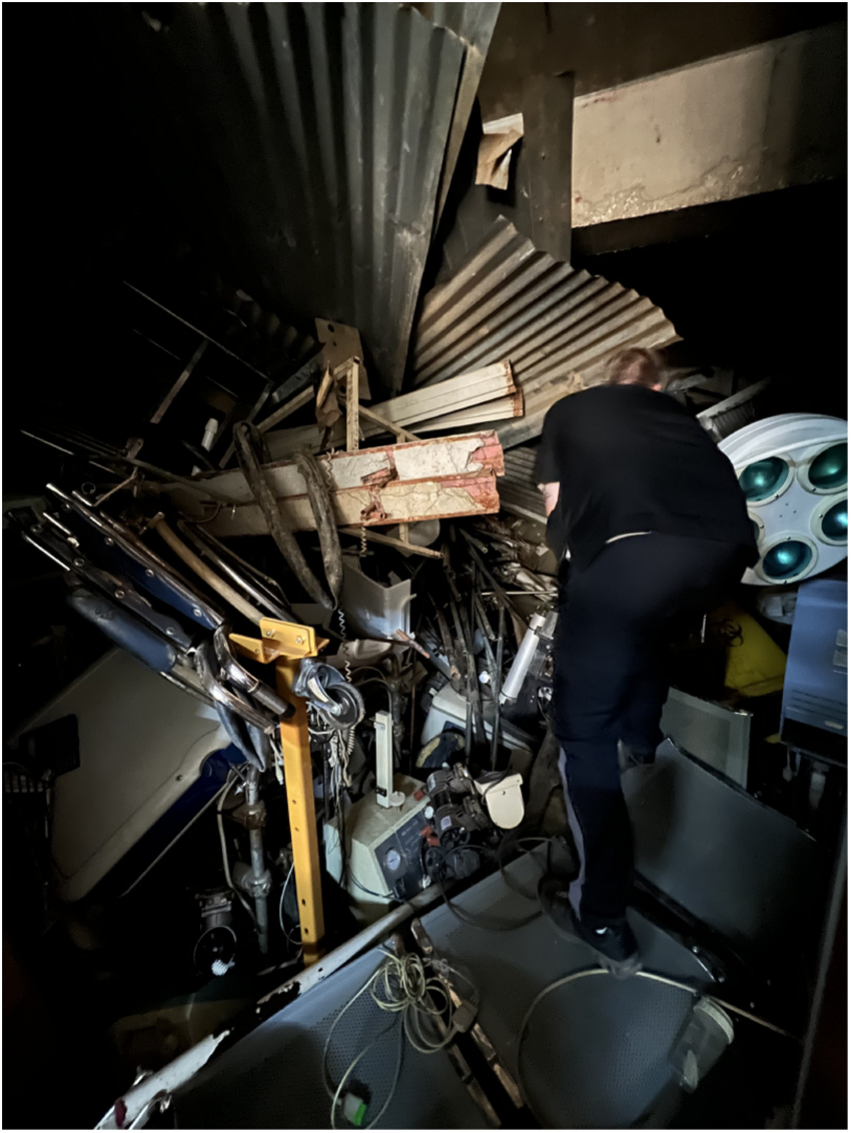
Volunteer biomedical technician in search of spare parts from the hospital equipment graveyard.

In their day-to-day work, volunteers identified and repaired faulty equipment throughout the hospitals. Lacking prior equipment histories, volunteers relied on direct troubleshooting. They carried essential tools and spare parts to enable immediate repairs whenever possible. When specific components were needed, they searched local storage (Figure 1) or sourced replacements from nearby vendors. Detailed documentation was kept for each repair, including device specifications, fault description, and repair actions taken.

### 2.3 Environmental sustainability assessment

An environmental sustainability assessment was conducted for a shipment of medical equipment transported in a container from Rigshospitalet, Copenhagen, Denmark, to Kampala, Uganda. The carbon footprint in terms of Global Warming Potential over a 100-year timeframe (GWP100) was estimated using the IPCC 2021 method, as implemented in the Ecoinvent database. It is expressed as kg ${\mathrm{CO}}_{2}$-equivalents (kg ${\mathrm{CO}}_{2}$e), meaning that the weighted sum of greenhouse gas emissions from the shipment has a GWP100 corresponding to X kg of ${\mathrm{CO}}_{2}$. The assumptions and methods underlying the estimate are detailed below.

The weight of the container filled with medical equipment was assumed to be 6 metric tons, based on prior experience with EWH, where three 40-foot high-cube containers were shipped to Ethiopia. Each container weighed approximately 6 tons and could fit the following contents: 70 larger items (e.g., beds and wheelchairs), 70 smaller items (e.g., endoscopy and surgical tools), and 200 miscellaneous hospital articles (e.g., gloves, syringes, and glasses).

The shipping route and distances from Rigshospitalet to Kampala were determined using Google Maps, Maersk’s route planning tools at Maersk.com, and Ports.com. The route comprised the following sub-routes:

1. Rigshospitalet, Denmark to Kalundborg, Denmark (121 km by truck).
2. Kalundborg, Denmark to Mombasa Terminal, Kenya via Bremerhaven, Port Tangier Mediterranee, and Salalah Terminal (14,534.5 km by container ship).
3. Mombasa Terminal, Kenya to Kampala, Uganda (1,164 km by truck).

Emission factors in terms of kg ${\mathrm{CO}}_{2}$e per ton-kilometer (kg ${\mathrm{CO}}_{2}$e/tkm) for each sub-route were obtained from the Ecoinvent database (ICCP 2021 method), using the following processes:

**Sub-route 1:** Transport by lorry (EURO4 class), with an emission factor of 0.14520 kg ${\mathrm{CO}}_{2}$e/tkm.

**Sub-route 2:** Transport by container ship, with an emission factor of 0.01016 kg ${\mathrm{CO}}_{2}$e/tkm.

**Sub-route 3:** Transport by lorry (EURO3 class), with an emission factor of 0.15729 kg ${\mathrm{CO}}_{2}$e/tkm.

The total GWP100 was calculated as the product of the container weight (6 tons), the distances traveled along each sub-route, and the respective emission factors for each transport mode.

## 3 Results

### 3.1 Litterature review

**Figure 2:**
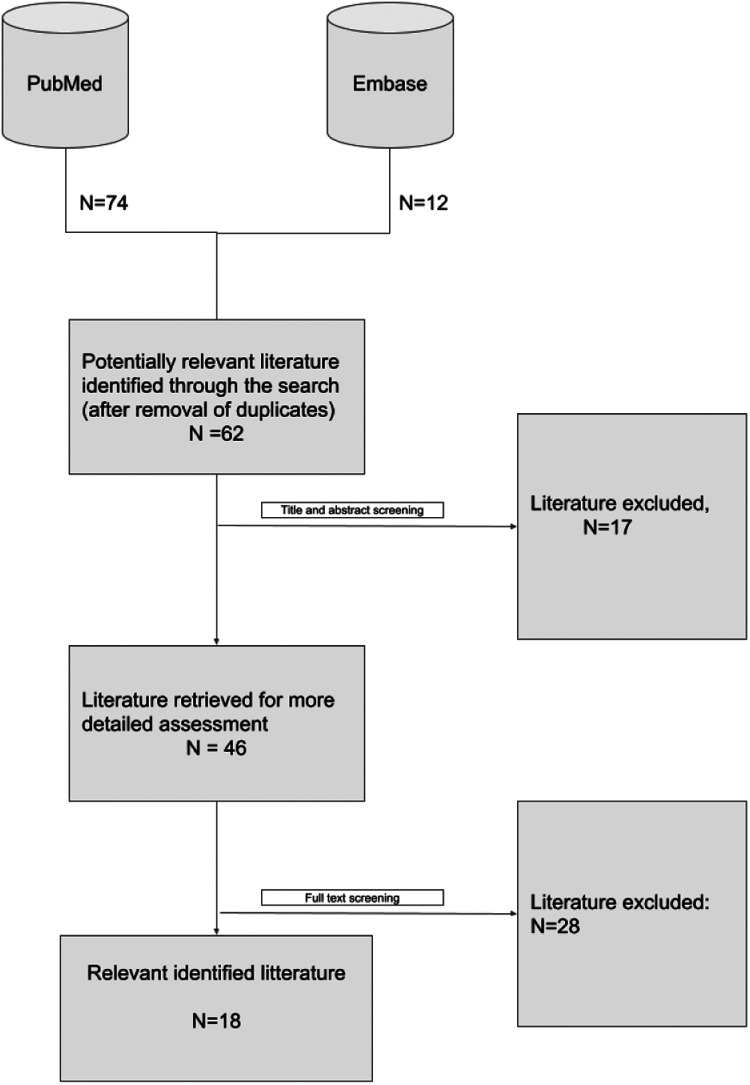
PRISMA plot of screening process.

A total of 18 articles were included following the PRISMA screening process (Figure 2). Each article was assessed for relevance to the five predefined research questions (Table 1).

The articles were categorized according to the research questions they addressed, providing a structured overview of the current evidence base within LMIC healthcare sustainability and medical equipment donation. The distribution is as follows:

RQ1 (Sustainability metrics): Addressed by 15 of the 18 articles.

RQ2 (Affordability): Addressed by 9 articles.

RQ3 (Recycling and reuse): Addressed by 6 articles.

RQ4 (Social and economic barriers): Addressed by 15 articles.

RQ5 (Innovations): Addressed by 14 articles.

This classification highlights that most studies focused on sustainability metrics (RQ1), systemic barriers (RQ4), and innovation (RQ5), while affordability (RQ2) and recycling strategies (RQ3) received comparatively less attention. A detailed breakdown of each article’s alignment with the research questions is presented in Table 2.

**Table 2:** Included articles categorized by relevance to each research question (RQ1–RQ5).

Related RQ	Reference
RQ1, RQ4 & RQ5	Sharma et al. (4)
RQ1 & RQ4	Zolo et al. (5)
RQ1, RQ4 & RQ5	Labrique et al. (6)
RQ2, RQ3 & RQ5	Samenjo et al. (7)
RQ1, RQ2 & RQ4	Bauserman et al. (8)
RQ1, RQ4 & RQ5	Webber et al. (9)
RQ4 & RQ5	Ameso (10)
RQ1, RQ2 & RQ4	Reuland et al. (11)
RQ3, RQ4 & RQ5	Ongaro et al. (12)
RQ1, RQ2, RQ4 & RQ5	Ditta et al. (13)
RQ1, RQ4 & RQ5	Martins et al. (14)
RQ1, RQ3, RQ4 & RQ5	Faktor et al. (15)
RQ1 & RQ2	Fasseeh et al. (16)
RQ4 & RQ5	Bijlmakers et al. (17)
RQ1, RQ2, RQ3, RQ4 & RQ5	Oturu et al. (18)
RQ1, RQ4 & RQ5	Lister et al. (19)
RQ3, RQ4 & RQ5	de Cates et al. (20)
RQ3, RQ4 & RQ5	Calvache (21)

### 3.2 Equipment observations: inventory assessment

Reports from two on-site observations on medical equipment have been categorized and counted, one from a Regional Referral Hospital in Mbarara and another from a Private Hospital in Masaka. The medical equipment has been categorized into nine types of medical equipment categories. These categories and respective counts can be found in Table 3 for the Mbarara Regional Referral Hospital and the St. Joseph’s Kitovu Private Hospital. In total, 1,306 and 123 pieces of medical equipment were assessed from the hospital inventory lists, respectively.

**Table 3:** Comparison of the medical equipment inventory lists from St. Joseph’s Hospital Kitovu and Mbarara Regional Referral Hospital, categorized based on on-site observations.

Category	St. Joseph’s Hospital Kitovu	Mbarara Regional Referral Hospital
Oxygen concentrators	30	156
Suction machines	13	35
Diagnostic laboratory equipment	13	62
Monitoring devices	12	174
Infant care equipment	11	48
Surgical equipment	9	31
Sterilization equipment	6	46
Imaging equipment	5	8
Weighing scales	3	74

As a result of the volunteer biomedical technicians’ stay, reports on repaired medical equipment have been collected and analyzed. A total of 28 pieces of medical equipment were repaired in Mbarara and 23 in Masaka. EWH estimates this work caused savings of $102.000 in repair costs.

Some of the assessed equipment was deemed unrepairable. This equipment was abandoned most often due to missing spare parts. At Mbarara, this acquainted for a total of 24 pieces of equipment, and for Masaka, a total of 12. The most common cause of medical equipment failure was identified as issues related to an insufficient supply of equipment power. This included blown fuses, worn batteries, or power supply problems. Mechanical failures were the second most reported, including broken heating elements, compressors, or worn-out parts.

A description of some repaired equipment based on type and its corresponding repair descriptions has been provided in Supplementary Material S2, illustrating some of the common causes of equipment failure.

### 3.3 Environmental sustainability assessment

The route for a 6-ton container shipped from Rigshospitalet, Copenhagen, Denmark to Kampala, Uganda comprised the following sub-routes, with corresponding estimated GWP100 carbon footprints:

1. 121 km by truck, Rigshospitalet to Kalundborg: 105.4 kg ${\mathrm{CO}}_{2}$e.
2. 14,534.5 km by container ship: Kalundborg to Mombasa Terminal: 886.3 kg ${\mathrm{CO}}_{2}$e.
3. 1,164 km by truck: Mombasa Terminal to Kampala: 1098.5 kg ${\mathrm{CO}}_{2}$e.

The total estimated GWP100 for the shipment was **2,090.3 kg CO_2_e**.

## 4 Discussion

Improving healthcare in LMICs requires more than good intentions. It requires sustainable, long-term strategies that consider not just the economic factors but also the environmental impact. Healthcare needs continue to grow in these regions and it’s essential that donations of medical equipment are planned with sustainability in mind, ensuring that they offer meaningful, lasting support rather than short-lived relief.

A significant concern surrounding medical equipment donations is the excessive waste generated due to inadequate planning and lack of infrastructure. It is estimated that up to 80% of medical devices in sub-Saharan Africa are donated, yet many are unsuitable and ultimately contribute to medical waste rather than enhancing healthcare delivery (7). The World Health Organization (WHO) reports that as much as 70% of donated medical equipment in sub-Saharan Africa is not used effectively, often due to missing technical support and inadequate user training (9). These findings highlight the need for a shift in donation strategies, focusing on sustainability rather than short-term solutions with insufficient collaboration between donor and recipient.

One critical factor in improving donation effectiveness is addressing the lifecycle of medical equipment, from delivery to disposal. Current literature emphasizes the environmental and health hazards posed by improper disposal of single-use devices, particularly in settings lacking regulatory frameworks (12). A potential solution to mitigate this is the implementation of a "reverse logistics" model, where donors commit to taking back expired or defunctioning devices, ensuring responsible disposal and minimizing environmental harm.

Medical professionals in sub-Saharan countries exhibit a strong willingness to learn about sustainability but lack formal education on the topic. A study in South Africa found that while healthcare professionals expressed a significant interest in sustainability practices, they lacked the necessary knowledge to implement them effectively (19). Integrating sustainability education into medical and technical training programs could bridge this gap and lead to long-term improvements in healthcare waste management.

Another pressing challenge in LMIC healthcare settings is the availability of consistent electricity and infrastructure. Many medical devices require stable power sources, yet power outages remain a significant barrier to effective healthcare delivery (4). This highlights the necessity for donors and manufacturers to consider alternative energy solutions, such as solar-powered medical equipment, which could enhance reliability and sustainability in resource-limited settings.

Beyond equipment donation, broader issues related to healthcare infrastructure must be addressed. Surgery and anesthesia, fundamental components of universal healthcare coverage, require not only functional medical devices but also a stable supply chain, oxygen availability, and facility management (22). Thus, any effective donation model must consider these supporting factors to ensure long-term functionality of the equipment provided.

Innovative approaches, such as drone-based healthcare solutions, have been proposed to bypass traditional infrastructural challenges, particularly in remote regions (10). However, while these technologies show promise, further research is needed to assess their environmental impact and ensure their sustainability.

To enhance sustainability in medical equipment donations, manufacturers must shift their focus towards designing devices tailored to LMIC settings. Many devices are currently designed for high-income countries (HICs), resulting in the equipment being ineffective or impractical in resource-limited environments (23). Designing devices with minimal maintenance requirements, modular spare parts, and easy-to-follow repair manuals could significantly improve usability and longevity of medical equipment in resource-limited settings. Additionally, a “Train-the-Trainer” model has been suggested to equip local healthcare personnel with the skills necessary to maintain and repair medical devices, fostering long-term self-sufficiency (9).

While short-term medical missions (STMMs) have raised concerns about sustainability and long-term impact, initiatives such as Engineering World Health (EWH) demonstrate that well-structured, sustainability-focused work can contribute meaningfully to healthcare improvements (15). By emphasizing repair, training, and capacity-building rather than one-time interventions, self-funded and sustainability-driven STMMs can play a role in strengthening local healthcare infrastructure and ensuring long-term benefits. Benefits of programs like EWH and STMMs is the opportunity for insights in LMICs and building of relations and partnerships. Relations are one of the key elements in a meaningful impact for the healthcare systems in LMICs.

Medical equipment donations ought to be re-evaluated with a focus on sustainability, education, infrastructure integration and strong relations. Instead of viewing donations as a one-time act of aid, they should be embedded in a broader framework of responsible partnerships, preventive maintenance, and long-term healthcare development. A study evaluating 112,040 medical devices across 16 countries found that, 38.4% of equipment was out of service due to factors such as lack of maintenance and spare parts availability (20). Addressing these shortcomings requires donors to tailor their contributions to the specific needs of the recipient community, ensuring a meaningful and lasting impact.

In conclusion, the paradigm of medical equipment donations must evolve towards sustainability-driven practices. Key solutions include responsible disposal programs, improved training initiatives, infrastructure-focused support, and context-aware device design. By integrating these considerations into future aid efforts, healthcare in the Global South can be strengthened more effectively, fostering long-term resilience and sustainability in LMIC healthcare systems.

## 5 The 8 theses

Based on our field observations and a review of existing literature on sustainable medical equipment donation and healthcare development in LMICs, the following eight principles have been formulated for future sustainable medical equipment donation. These suggested guidelines, to help maximize the long-term impact of medical equipment donations, highlight key factors that can support more effective, context-aware, and sustainable contributions to healthcare systems in the Global South.

1. **Sustainable implementation is the donor’s responsibility.** Sustainable medical equipment donations must be planned, implemented, and supported by the donor. The responsibility for long-term impact can never lie with the recipient.
2. **Donor–recipient collaboration is essential.** A strong professional relationship and ongoing communication between the donor and recipient are crucial for ensuring trust and a positive long-term outcome.
3. **Context matters: infrastructure must be understood.** Donors must conduct thorough assessments of the recipient’s local infrastructure, including energy, water, waste management, and human resources, to ensure compatibility and feasibility of donated equipment.
4. **Installation and technical training are non-negotiable.** Donations must be accompanied by appropriate installation and/or training of personnel responsible for setup, operation, and maintenance.
5. **Lifecycle responsibility and appropriate design lie with the donor.** Donors are responsible for ensuring that equipment is context-appropriate. This includes considering usability in low-resource settings, availability of spare parts, feasibility of repairs, and access to trained personnel. HIC equipment often underperforms in LMIC settings due to environmental and infrastructural mismatches.
6. **Environmental impact must be evaluated.** The carbon footprint of production, shipping, and the disposal of medical equipment must be weighed against the projected local health benefits. Emissions and environmental consequences are an ethical component of donations.
7. **Legal compliance is part of ethical donation.** It is the donor’s duty to investigate and comply with local laws, including import regulations and taxation policies, in the recipient country.
8. **Poor quality or outdated equipment is rarely appropriate** If medical equipment is considered too outdated, worn or unreliable for use in the donor’s setting, it should not be assumed to be acceptable for the recipient. Donations should meet basic standards of safety, functionality, and relevance.
