# Supplementary material for: Medical equipment in the global south: perspective of sustainability and donations
Source: Front Health Serv. 2025 Sep 4;5:1638305. doi: 10.3389/frhs.2025.1638305 (PMC12443703; doi:10.3389/frhs.2025.1638305)
Supplement: Supplementary file 1 [file Datasheet1.pdf]

DB	DOI	Title	Author	Year	Method	Innovation	RQ Relevance	Avg. Score (Pass
PubMed	10.1016/j.jaapos.	A cost analysis of pediatric cataract surgery at two child eye health tertiary facilities in Africa	Evans CT, Lenha	2014	3,50	3,50	2,50	3,17
PubMed	10.1186/s40545-	A rapid assessment of the National Regulatory Systems for medical products in the Southern African Development Community	Dube-Mwedzi S,	2020	3,00	3,50	2,50	3,00
PubMed	10.7759/cureus.5	Advancing Solar Energy for Primary Healthcare in Developing Nations: Addressing Current Challenges and Enabling Progress Through UNICEF and Collaborative Partnerships	Sharma L, Singh	2024	4,00	3,00	4,50	3,83
PubMed	10.1128/CMR.00	Antimicrobial Resistance Surveillance in Low- and Middle-Income Countries: Progres	Gandra S, Alvare	2020	4,00	2,50	4,00	3,50
PubMed	10.1002/hsr2.224	Assessing the presence of sustainability education in the curriculum of medical students and surgical trainees in Africa: A cross-sectional study	Zolo Y, Demissie	2024	4,50	4,50	5,00	4,67
Embase	Click here for full	Best practices and current implementation of emerging smartphone-based (bio)senso	Geballa-Koukoul<br>Ross G.M.S.<br>Bosman A.J.<br>Zhao Y.<br>Zhou H.<br>Nielen M.W.F.<br>Rafferty K.<br>Elliott C.T.<br>Salentijn G.I.	2023	4,00	3,50	3,00	3,50
PubMed	10.1186/s12992-	Best practices in scaling digital health in low and middle income countries	Labrique AB, Wa	2018	3,50	4,00	4,00	3,83
PubMed	10.3390/ijerph18	Climate Change and Health Preparedness in Africa: Analysing Trends in Six African Countries	Opoku SK, Leal F	2021	4,00	2,50	3,50	3,33
PubMed	10.26717/bjstr.20	Closing the Gaps on Medical Education in Low-Income Countries Through Informatio	Zavale BL, Spen	2019	3,50	4,00	3,50	3,67
PubMed	10.1371/journal.p	Corruption and informal sector households' participation in health insurance in Sierra	Jofre-Bonet M, K	2023	4,00	3,00	4,00	3,67
PubMed	10.3389/fmedt.20	Design of a syringe extension device (Chloe SED®) for low-resource settings in sub-Saharan Africa: a circular economy approach	Samenjo KT, Rar	2023	5,00	5,00	5,00	5,00
PubMed	10.1093/inthealth	Determining the utility and durability of medical equipment donated to a rural clinic in a low-income country	Bauserman M, H	2015	5,00	5,00	5,00	5,00
Embase	Click here for full	Developing strategies for sustainable medical equipment maintenance in under-resou	Webber C.M.<br>Martinez-Galvez<br>Higuita M.L.<br>Ben-Abraham E<br>Berry B.M.<br>Porras M.A.G.<br>Aristizabal S.<br>Asp A.<br>Lujan J.L.<br>Wilson J.W.	2020	3,50	4,00	5,00	4,17
PubMed	10.1080/1744169	Digital entanglements: Medical drones in African healthcare systems	Ameso EA.	2024	4,00	5,00	2,50	3,83
PubMed	10.1093/heapol/c	Energy access in Malawian healthcare facilities: consequences for health service delivery and environmental health conditions	Reuland F, Behnl	2020	3,50	4,00	4,00	3,83
PubMed	10.1039/d2lc003	Engineering a sustainable future for point-of-care diagnostics and single-use microflu	Ongaro AE, Ndlo	2022	3,50	4,00	5,00	4,17
PubMed	10.1016/j.jaapos.	Establishing a surgical outreach program in the developing world: pediatric strabismus surgery in Guatemala City, Guatemala	Ditta LC, Pereira	2015	3,50	4,00	4,50	4,00
PubMed	10.1186/s12893-	From emissions to incisions and beyond: the repercussions of climate change on sur	Martins RS, Poul	2023	4,00	4,00	5,00	4,33
PubMed	10.1016/j.sempe	Gaps and priorities in innovation for children's surgery	Fitzgerald TN, Za	2023	3,50	2,50	4,50	3,50
PubMed	10.1002/bjs.1104	Global surgery and the sustainable development goals	Roa L, Jumbam I	2019	2,00	2,00	1,00	1,67
Embase	Click here for full	Health technology assessment in Malaysia.	Sivalal S.	2009	N/A	N/A	N/A	
PubMed	10.1002/hpm.333	Healthcare systems strengthening in Africa: The call for action to achieve SDG 3	Olaniyi PO, Oluw	2022	3,50	3,00	4,00	3,50
FUJIFILM IVIZ		https://seelearn-emea.fujifilm.com/iviz-wireless/			1,00	1,00	2,50	1,50
PubMed	10.5334/aogh.39	Impact and Sustainability of Foreign Medical Aid: A Qualitative Study with Honduran H	Faktor KL, Payár	2023	3,50	3,50	5,00	4,00
Embase	Click here for full	Impact of health technology assessment implementation with a special focus on midd	Fasseeh A.N.<br>Saragih S.M.<br>Hayek N.<br>Brodovska S.<br>Ismail A.<br>ElShalakani A.<br>Abaza S.<br>Obeng G.D.<br>Ameyaw D.<br>Kalo Z.	2022	4,50	4,00	4,50	4,33
PubMed	10.1080/165497	Innovating to increase access to diabetes care in Kenya: an evaluation of Novo Nordi	Shannon GD, Ha	2019	4,00	4,00	3,00	3,67
PubMed	10.1017/S02664	INTEGRATE-HTA: A LOW- AND MIDDLE-INCOME COUNTRY PERSPECTIVE	Bijlmakers L, Mu	2017	3,00	4,00	4,50	3,83
Embase	Click here for full	KNOW ESSENTIALS - A novel algorithm for informed vaccine-related decision-makin	Mathew J.L.	2010	N/A	N/A	N/A	
PubMed	10.1186/s12913-	Mentorship and coaching to support strengthening healthcare systems: lessons learn	Manzi A, Hirschh	2017	3,50	3,00	2,00	2,83
PubMed	10.1136/bmjoper	National health information systems for achieving the Sustainable Development Goals	Suthar AB, Khalif	2019	3,50	3,00	3,50	3,33
PubMed	10.1371/journal.p	National medicines regulatory authorities financial sustainability in the East African Co	Ndomondo-Sigor	2020	3,50	3,00	2,00	2,83
PubMed	10.1186/s12889-	Principles for task shifting hypertension and diabetes screening and referral: a qualita	Ingenhoff R, Mur	2023	3,50	2,00	2,50	2,67
PubMed	10.1186/s13012-	Proceedings of the 8th Annual Conference on the Science of Dissemination and Impl	Chambers D, Sin	2016	N/A	N/A	N/A	
PubMed	10.1007/s12553-	Remanufacturing of single-use medical devices: a case study on cross-border collabo	Oturu K, Ijomah V	2022	4,50	4,00	5,00	4,50

Embase	https://dx.doi.org/10.1016/j.tipsro.2024.100264	Setting up a new radiation therapy centre in Malawi: Opportunities and challenges.	Tembo E.<br>Kyei K.A.<br>Thulu F.<br>Masamba L.<br>Chiwanda J.<br>Kuyeli S.<br>Nyirenda R.<br>Nyasosela R.<br>Mzikamanda R.<br>Ndarukwa S.	2024	4,00	2,50	3,00	3,17
PubMed	10.3390/ijerph19	South African Healthcare Professionals' Knowledge, Attitudes, and Practices Regarding Environmental Sustainability in Healthcare: A Mixed-Methods Study	Lister HE, Moste	2022	4,00	3,50	4,00	3,83
Embase	Click here for full	Sustainable equipment donation in otolaryngology in low-resource settings.	De Cates C.<br>- Gueroult A.M.<br>- Narantsolmon G	2024	4,50	4,50	5,00	4,67
PubMed	10.1177/0734242	Sustainable waste management of medical waste in African developing countries: A r	Chisholm JM, Za	2021	3,00	2,50	3,00	2,83
PubMed	10.1186/1471-24	Systematic review on what works, what does not work and why of implementation of	Aranda-Jan CB,	2014	2,50	1,50	2,00	2,00
PubMed	10.3389/fped.202	The case of the neonate vs. LMIC medical academia-a jury-style systematic review o	Amadi HO, Abioy	2024	3,50	3,50	4,00	3,67
Embase	Click here for full	The environmental responsibility of modern anesthesiology and perioperative care.	Calvache J.A.	2023	4,50	4,00	3,50	4,00
PubMed	10.1093/intqhc/m	The future of health systems to 2030: a roadmap for global progress and sustainability	Braithwaite J, Ma	2018	2,00	4,50	2,50	3,00
PubMed	10.1186/s12939-	The right of access to healthcare: an analysis of how legal and institutional frameworks constrain or facilitate access to healthcare for residents in border areas in the East African Community	Ssengooba F, Ba	2022	2,50	1,50	1,00	1,67
PubMed	10.1186/s12913-	The rise of resilient healthcare research during COVID-19: scoping review of empirica	Ellis LA, Saba M,	2023	4,00	1,50	2,00	2,50
Embase	Click here for full	UNDERSTANDING THE CONTEXTUAL FACTORS THAT INFLUENCE VILLAGE HE	Scott K.<br>- Sheikh K.<br>- George A.<br>- Garimella S.<br>- Mondal S.<br>- Patel G.<br>- Ved R.	2016	2,50	2,00	2,00	2,17

**Included articles**	**Excluded articles**
18	28
