# Supplementary material for: Medical equipment in the global south: perspective of sustainability and donations
Source: Front Health Serv. 2025 Sep 4;5:1638305. doi: 10.3389/frhs.2025.1638305 (PMC12443703; doi:10.3389/frhs.2025.1638305)
Supplement: Supplementary file 2 [file Datasheet2.pdf]

Annex 2 - Inventory Lists
Kitovu Inventory List

Date	Customer	Department	Activity	Equipment	Manufacturer	Model	SerialNumber	In use?	Status	Comment	Supplier
2024-07-04	Kitovu Hospital	Divine Mercy		Weighing scale	Seca	7621019008	SN10000000434265	Y	Functional		Purchased
2024-07-04	Kitovu Hospital	Divine Mercy		18 Beds	N/A	N/A	N/A	Y	Functional		Purchased
2024-07-04	Kitovu Hospital	Divine Mercy		Blood pressure monitor	Scian	LD520	2252001921	Y	Functional		Purchased
2024-07-04	Kitovu Hospital	Divine Mercy		SPO2 monitor	Elektro	ELK30B	SN:19120500073	N	Non-functional	Sensor is broken	Purchased
2024-07-04	Kitovu Hospital	Divine Mercy		SPO2 monitor	MEDITECH	FOs2 plus	N/A	Y	Functional		Purchased
2024-07-04	Kitovu Hospital	Divine Mercy		Nebulizer	Detter	BT8002	BT800218050036	Y	Functional		Purchased
2024-07-04	Kitovu Hospital	Divine Mercy		Patient Monitor	MEDITECH	MD9012	MD1491391	Y	Functional		Purchased
2024-07-04	Kitovu Hospital	Surgical ward		Patient Monitor	BLT	M9000A	M013E007543	Y	Functional		Purchased
2024-07-04	Kitovu Hospital	Surgical ward		Suction Machine	SAM	BSEN60601-1	0720-2404	Y	Functional	Missing a cap for pressure	Purchased
2024-07-04	Kitovu Hospital	Surgical ward		27 Beds	N/A	N/A	N/A	Y	Functional		Purchased
2024-07-04	Kitovu Hospital	Surgical ward		Oxygen concentrator	Krober	Aeropres5	A517043965	Y	Functional		Purchased
2024-07-04	Kitovu Hospital	Surgical ward		Oxygen concentrator	Krober	Aeropres5	A517043961	Y	Functional		Purchased
2024-07-04	Kitovu Hospital	Neonatal		Phototherapy	Drager	Babytherm 8000	N/A	Y	Functional		Purchased
2024-07-04	Kitovu Hospital	Neonatal		Phototherapy	MTTS	Firefly	N/A	Y	Functional		Purchased
2024-07-04	Kitovu Hospital	Neonatal		Phototherapy	MTTS	Firefly	F17123002	Y	Functional		Purchased
2024-07-04	Kitovu Hospital	Neonatal		Infant radiant warmer	MTTS	Wallaby	W18135005	Y	Functional		Purchased
2024-07-04	Kitovu Hospital	Neonatal		Infant radiant warmer	Vickers Medical	185	N/A	Y	Functional		Purchased
2024-07-04	Kitovu Hospital	Neonatal		Infant radiant warmer	Dyson Med	BN100	1110802013	Y	Functional		Purchased
2024-07-04	Kitovu Hospital	Neonatal		Infant radiant warmer	Dyson Med	BN100A	1230201006	Y	Functional		Purchased
2024-07-04	Kitovu Hospital	Neonatal		Patient Monitor	N/A	YK-8000G	21122409070107	Y	Functional		Purchased
2024-07-04	Kitovu Hospital	Neonatal		Baby CPAP	diamedica	Baby CPAP 20	A20A280008US	Y	Functional		Purchased
2024-07-04	Kitovu Hospital	Neonatal		Baby CPAP	N/A	FREO2	N/A	Y	Functional		Purchased
2024-07-04	Kitovu Hospital	Neonatal		Baby CPAP	N/A	FREO3	N/A	Y	Functional		Purchased
2024-07-04	Kitovu Hospital	Neonatal		Baby CPAP	N/A	FREO4	N/A	Y	Functional		Purchased
2024-07-04	Kitovu Hospital	Neonatal		Infant Incubator	N/A	FNBI-3000A(G)	240180201	Y	Functional		Donated
2024-07-04	Kitovu Hospital	Neonatal		Oxygen concentrator	Obelis	JAY-5	MZJ5D41997	Y	Functional		Purchased
2024-07-04	Kitovu Hospital	Neonatal		Oxygen concentrator	N/A	YRK-10L	N/A	Y	Functional		Purchased
2024-07-04	Kitovu Hospital	Neonatal		Oxygen concentrator	Obelis	JAY-6	MZJ5D33500	Y	Functional		Purchased
2024-07-04	Kitovu Hospital	Neonatal		3 Beds	N/A	N/A	N/A	Y	Functional		Purchased
2024-07-10	Kitovu Hospital	Radiology	Service	Ultrasound machine	Mindray	DC30	9P-08007446	Y	Functional		Purchased
2024-07-10	Kitovu Hospital	Radiology		Mammography X-ray	Bemems	BNG-RT	BMR-100311	Y	Functional		Purchased
2024-07-10	Kitovu Hospital	Radiology		CT	Siemens Healthcare	11061610	168387	Y	Functional		Purchased
2024-07-10	Kitovu Hospital	Radiology		Infusion pump	Medtron	Accutron CT-D	862022232	Y	Functional		Purchased
2024-07-10	Kitovu Hospital	Radiology		X-ray machine	Listem	BLD150RK	D5564	Y	Functional		Purchased
2024-07-10	Kitovu Hospital	Radiology		Bucku stand	Listem	BS20	N2282	Y	Functional		Purchased
2024-07-10	Kitovu Hospital	Childrens ward		30 beds	N/A	N/A	N/A	Y	Functional		Purchased
2024-07-10	Kitovu Hospital	Childrens ward		Slit lamp	N/A	N/A	N/A	Y	Functional		Purchased
2024-07-10	Kitovu Hospital	Childrens ward		1 bed	N/A	N/A	N/A	N	Not functioning		Purchased
2024-07-10	Kitovu Hospital	Childrens ward		Dental chair	N/A	N/A	N/A	Y	Functional		Purchased
2024-07-10	Kitovu Hospital	Childrens ward		Autoclave	Lorma	M072	490334	Y	Functional		Purchased

2024-07-10	Kitovu Hospital	Childrens ward		Patient monitor	Meditech	M800E	MD1489218	Y	Functional		Purchased
2024-07-10	Kitovu Hospital	Childrens ward		Oxygen Concentrator	Devilbiss	515KS	N/A	Y	Functional		Purchased
2024-07-10	Kitovu Hospital	Childrens ward		Oxygen Concentrator	Devilbiss	525KS	N/A	Y	Functional		Purchased
2024-07-10	Kitovu Hospital	Childrens ward		Oxygen Concentrator	Devilbiss	515KS	N/A	Y	Functional		Purchased
2024-07-10	Kitovu Hospital	Childrens ward		Oxygen Concentrator	Devilbiss	515KS	N/A	Y	Functional		Purchased
2024-07-10	Kitovu Hospital	Childrens ward		Oxygen Concentrator	Devilbiss	515KS	N/A	Y	Functional		Purchased
2024-07-10	Kitovu Hospital	Childrens ward		Oxygen Concentrator	Devilbiss	525KS	B22B160058KS	Y	Functional		Purchased
2024-07-10	Kitovu Hospital	Childrens ward		Oxygen Concentrator	Obelis	JAY-5	MZJ5D34681	Y	Functional		Purchased
2024-07-10	Kitovu Hospital	Laboratory		Blood analyzer, HIV	Abbott	Pima-analyzer	NAT-04002238	Y	Functional		Purchased
2024-07-10	Kitovu Hospital	Laboratory		PIMA™ ANALYSER	Abbott	Pima-analyzer	NAT-04005058	Y	Functional		Purchased
2024-07-10	Kitovu Hospital	Laboratory		PIMA™ ANALYSER	Alere	Pima	Pima-D-007799	Y	Functional		Purchased
2024-07-10	Kitovu Hospital	Laboratory		Blood analyzer, CD4 counter	Becton	BDFacs Presto	R65100001337	Y	Functional		Purchased
2024-07-10	Kitovu Hospital	Laboratory		Centrifuge	Wincom	C-802D	N/A	Y	Functional		Purchased
2024-07-10	Kitovu Hospital	Laboratory		Microscope	Olympus	CX21	8J08605	Y	Functional		Purchased
2024-07-10	Kitovu Hospital	Laboratory		Digital colorimeter	WPA	CO7000	1132	Y	Functional		Purchased
2024-07-10	Kitovu Hospital	Laboratory		CBC Machine	Pulse	Hemato 3- 80T	EC382211JVNC88	Y	Functional		Purchased
2024-07-10	Kitovu Hospital	Laboratory		Biochemistry analyzer	Roche	cobas c 111	N/A	Y	Functional		Purchased
2024-07-10	Kitovu Hospital	Laboratory		Biochemistry analyzer	Seamaty	SD1	128002962	Y	Functional		Purchased
2024-07-10	Kitovu Hospital	Laboratory		Centrifuge	Hettich	EBA200	0037674-06	Y	Functional		Purchased
2024-07-10	Kitovu Hospital	Laboratory		Hormonal analyzer	Boditech	IchtomaII	N/A	Y	Functional		Purchased
2024-07-10	Kitovu Hospital	Laboratory		I-chamber heater	Boditech	I-chamber	N/A	Y	Functional		Purchased
2024-07-10	Kitovu Hospital	Laboratory		Microscope	Motic	BA200	N/A	Y	Functional		Donated
2024-07-10	Kitovu Hospital	Laboratory		Microscope	Motic	N/A	N/A	Y	Functional		Purchased
2024-07-10	Kitovu Hospital	Laboratory		Water bath	Memmert	N/A	N/A	Y	Functional		Purchased
2024-07-10	Kitovu Hospital	Laboratory		Autoclave	All American	50X	0000748	Y	Functional		Purchased
2024-07-10	Kitovu Hospital	Laboratory		Incubator	Memmert	N/A	N/A	Y	Functional		Purchased
2024-07-10	Kitovu Hospital	Laboratory		Microscope	Olympus	CX21 FS1	8J08878	Y	Functional		Purchased
2024-07-10	Kitovu Hospital	Laboratory		Biological safety cabinet	Biobase	11231BBC86	BSC31A1903115A	Y	Functional		Purchased
2024-07-10	Kitovu Hospital	Medical Ward		18 beds	N/A	N/A	N/A	Y	Functional		Purchased
2024-07-10	Kitovu Hospital	Medical Ward		Oxygen concentrator	Devilbiss	525KS	B204130150KS	Y	Functional		Purchased
2024-07-10	Kitovu Hospital	Medical Ward		Oxygen concentrator	Olive	OLV-5	N/A	Y	Functional		Purchased
2024-07-10	Kitovu Hospital	Medical Ward		Oxygen concentrator	N/A	N/A	N/A	Y	Functional		Purchased
2024-07-10	Kitovu Hospital	Medical Ward		Oxygen concentrator	N/A	N/A	N/A	Y	Functional		Purchased
2024-07-10	Kitovu Hospital	Maternity ward		Weighing scale	N/A	N/A	N/A	Y	Functional		Purchased
2024-07-10	Kitovu Hospital	Maternity ward		29 beds	N/A	N/A	N/A	Y	Functional		Purchased
2024-07-10	Kitovu Hospital	Maternity ward		1 bed	N/A	N/A	N/A	N	Not functioning		Purchased
2024-07-10	Kitovu Hospital	Labour ward		Infant warmer	Ohmeda	Ohio	N/A	Y	Functional		Purchased
2024-07-10	Kitovu Hospital	Labour ward		Infant weighing scale	Seca	N/A	N/A	Y	Functional		Purchased
2024-07-10	Kitovu Hospital	Labour ward		Oxygen concentrator	Obelis	JAY-5	MZJ5D32074	Y	Functional		Purchased
2024-07-10	Kitovu Hospital	Labour ward		Suction machine	MGE	SAM 14	N/A	Y	Functional		Purchased
2024-07-10	Kitovu Hospital	Theatre		Patient monitor	Meditech	MD9012	MD1493285	Y	Functional		Purchased
2024-07-10	Kitovu Hospital	Theatre		Operating lamp	N/A	N/A	N/A	Y	Functional		Purchased

2024-07-10	Kitovu Hospital	Theatre		Operating lamp	Bicakcilar	1150A	32	Y	Functional		Purchased
2024-07-10	Kitovu Hospital	Theatre		Oxygen Concentrator	Devilbiss	515KS	N/A	Y	Functional		Purchased
2024-07-10	Kitovu Hospital	Theatre		Suction machine	CAMI	H 350	48456	Y	Functional		Purchased
2024-07-10	Kitovu Hospital	Theatre		Electro cautery	BPL	SurgiX-E2	NQTA1D1070	Y	Functional		Purchased
2024-07-10	Kitovu Hospital	Theatre		Patient monitor	EDAN	X10	261590-M22A10830073	Y	Functional		Purchased
2024-07-10	Kitovu Hospital	Theatre		Operating lamp	HFMED	Shadowless operating lamp		Y	Functional		Purchased
2024-07-10	Kitovu Hospital	Theatre		Operating lamp	Bicakcilar	300A	268	Y	Functional		Purchased
2024-07-10	Kitovu Hospital	Theatre		Anaesthesia Machine	AEOMED	Aeon7200	Aeon7200xxzv00029	Y	Functional		Purchased
2024-07-10	Kitovu Hospital	Theatre		Anaesthesia Machine	OES medical	UAM	21402-030	Y	Functional		Purchased
2024-07-10	Kitovu Hospital	Theatre		Suction machine	SAM	MG electric	0821-1111	Y	Functional		Purchased
2024-07-10	Kitovu Hospital	Theatre		Autoclave	KSG	Sterialisator	2496	Y	Functional		Purchased
2024-07-10	Kitovu Hospital	Theatre		Autoclave	All American	75X	3780	Y	Functional		Purchased
2024-07-10	Kitovu Hospital	Theatre		Autoclave	N/A	N/A	N/A	Y	Functional		Purchased
2024-07-10	Kitovu Hospital	Theatre		Operating lamp	Bicakcilar	1150A	31	Y	Functional		Purchased
2024-07-10	Kitovu Hospital	Theatre		Oxygen Concentrator	Devilbiss	525KS	B204140018KS	Y	Functional		Purchased
2024-07-10	Kitovu Hospital	Theatre		Anaesthesia Machine	OES medical	UAM	N/A	Y	Functional		Purchased
2024-07-10	Kitovu Hospital	Theatre		Patient monitor	Shenzhen	UP7000	J3300KD00190	Y	Functional		Purchased
2024-07-10	Kitovu Hospital	Theatre		Patient monitor	EDAN	X10	261590-M22A10830075	Y	Functional		Purchased
2024-07-10	Kitovu Hospital	Theatre		Suction machine	SAM	MG electric	0722-1095	Y	Functional		Purchased
2024-07-10	Kitovu Hospital	Theatre		Infant scale	N/A	ACS-20B-YE	N/A	Y	Functional		Purchased
2024-07-10	Kitovu Hospital	Theatre		Patient monitor	Contec	CMS7000	20120600044	Y	Functional		Purchased
2024-07-10	Kitovu Hospital	Theatre		Oxygen Concentrator	Devilbiss	525KS	B206170363KS	Y	Functional		Purchased
2024-07-10	Kitovu Hospital	Theatre		Endoscope	Olympus	CLV-U40	N/A	Y	Functional		Purchased
2024-07-10	Kitovu Hospital	Theatre		Endoscope	Olympus	CV-240	N/A	Y	Functional		Purchased
2024-07-11	Kitovu Hospital	Workshop		Oxygen concentrator	Devilbiss	525KS	N/A	N	Non-functional	Compressor and Zeiolite ch	Purchased
2024-07-12	Kitovu Hospital	Workshop		Oxygen concentrator	Yuerkang	YRK-10L	21053409	N	Non-functional		Purchased
2024-07-12	Kitovu Hospital	Workshop		Oxygen concentrator	Yuerkang	YRK-10L	N/A	N	Non-functional		Purchased
2024-07-12	Kitovu Hospital	Workshop		Oxygen concentrator	Devilbiss	525KS	F642718KS	N	Non-functional		Purchased
2024-07-12	Kitovu Hospital	Workshop		Suction machine	MGE	SAM 14	0421-0709	N	Non-functional		Purchased
2024-07-12	Kitovu Hospital	Workshop		Suction machine	MGE	SAM 14	0720-2418	N	Non-functional		Purchased
2024-07-12	Kitovu Hospital	Workshop		Suction machine	MGE	SAM 14	N/A	N	Non-functional		Purchased
2024-07-12	Kitovu Hospital	Workshop		Suction machine	MGE	SAM 14	N/A	N	Non-functional		Purchased
2024-07-12	Kitovu Hospital	Workshop		Suction machine	MGE	SAM 15	N/A	N	Non-functional		Purchased
2024-07-12	Kitovu Hospital	Workshop		Centrifuge	Wincom	C-801P		N	Non-functional		Purchased
2024-07-12	Kitovu Hospital	Workshop		Blood pressure monitor	Nantong	LD7	1000700974	N	Non-functional		Purchased
2024-07-12	Kitovu Hospital	Workshop		Infant weighing scale	Kinloo	EBSA-20	N/A	N	Non-functional		Purchased
2024-07-12	Kitovu Hospital	Workshop		Patient monitor	Shenzhen	UP7000	J3300KD00232	N	Non-functional		Purchased
2024-07-12	Kitovu Hospital	Workshop store		Oxygen Concentrator	Devilbiss	525KS	F683849KS	N	Non-functional	Gulv	Purchased
2024-07-12	Kitovu Hospital	Workshop store		Oxygen Concentrator	Devilbiss	525KS	F650585KS	N	Non-functional	Hylde 1v	Purchased
2024-07-12	Kitovu Hospital	Workshop store		Oxygen Concentrator	Devilbiss	525KS	F650516KS	N	Non-functional	Hylde 2v	Purchased
2024-07-12	Kitovu Hospital	Workshop store		Oxygen Concentrator	Devilbiss	525KS	F642700KS	N	Non-functional	Hylde 3v	Purchased
2024-07-12	Kitovu Hospital	Workshop store		Oxygen Concentrator	Devilbiss	515AKS	XXXX608KS	N	Non-functional	Gulv	Purchased

2024-07-12	Kitovu Hospital	Workshop store		Oxygen Concentrator	Devilbiss	515KS	H30666KS	N	Non-functional	Gulv	Purchased
2024-07-12	Kitovu Hospital	Workshop store		Oxygen Concentrator	Devilbiss	515KS	H35484KS	N	Non-functional	Gulv	Purchased
2024-07-12	Kitovu Hospital	Workshop store		Oxygen Concentrator	AirZep	NewLife	926303	N	Non-functional	Hylde 4v	Purchased
2024-07-13	Kitovu Hospital	Workshop store		Suction Machine	CAMI	Hospivac400	1346	N	Non-functional		Purchased
2024-07-14	Kitovu Hospital	Workshop store		Suction Machine	YUWell	7A-23D		N	Non-functional		Purchased
2024-07-15	Kitovu Hospital	Workshop store		Operating lamp	Bicakcilar	1150A	18	N	Non-functional		Purchased

Mbarara Regional Referal Hospital, Inventory List Summarised

Type	Equipment	Count
	Air Compressor	9
	Anaesthesia Machine With Oxyger	2
	Anaesthesia Cart	9
	Anaesthesia Machine With Monitor	6
	CD4 Count Machine	12
	Heamatology Analyzer	18
	PCR System Analyzer (DNA)	13
	Analyzer, TB	2
	Urine Analyzer	1
	Arterial Blood	1
	Blood gas Analyzer	1
	Gas Machine (ABG)	1
	Audiometer	1
	Autoclave	45
	Auto Blood Culture Machine	2
	Automated Coagulation Analyzer	2
	Automated External Defibrillator	1
	Automatic Bacterial Identification M	3
	Automatic Pipette	17
	Blood Pressure Machine, Paediatr	16
	Baby COT (Crib)	5
	Baby Incubator	7
	Balance, Organs Weighing	1
	Hospital Bed, Bed	163
	Hospital Bed, Children	10
	Operation Bed, Electric	4
	Biological Safety Cabinet	28
	Blood Warmer	1
	Operation Bed, Electric	3
	BP Machine, Mercury	17
	Burn Out Machine, Oven	1
	Photographic Camera	1
	CD4 Count Machine	14
	Centrifugal Manual	6
	Analzyer, Chemistry	16
	Colorimeter	3
	Deep Freezer	1
	Defibrillator Tester	1
	Delivery Bed	30
	Dental X-Ray Machine	1
	Differential Counter	2
	Drip Stand	49
	Drug Cabinet	8
	ECG	2
	Suction Machine	35
	Examination Light	10
	GeneXpert Machine	8

Hematology Analyser	8
Glucometer	19
ICU Bed	2
Incubator	7
Infant Warmer	25
Infusion Pump	36
Refrigerator	34
LaryngoScope	2
Medical Gas Flow Meter	23
Microscope	35
Operating Light	18
MRI Machine	1
Nebulizer	9
Operation Lamp	4
Oxygen Concentrator	156
Oxygen Theraphy Apparatus	12
Patient Monitor	123
Phototherapy Unit	32
Pulse Oximeter	12
Vaccine Refrigerator	32
Stethoscope	5
Suction Apparatus	18
Ultrasound Machine	6
Ventilator	23
Weighing Scale	74
X-ray Machine	1
SUM	1306

RAW DATA FROM MBARARA REGIONAL REFERAL HOSPITAL

MOH - INVENTORY

Equipment Name	Type	System ID	Model Name	Model No.	Serial No.
Adaptive Communication Switches.	HSS	Mbarara-RRH-1420	NOUVAG		GD1889
Air Compressor, Dental	HSS	Mbarara-RRH-990	KAESAR	SK 25T	1563
Air Compressor, Dental	HSS	Mbarara-RRH-1254		V01507.K60.N110.000.20.0	V015-2020-001107
Air Compressor, dental	HSS	Rugazi-HC IV-28940			
Air Conditioner (Laminar flow type)	HSS	Mbarara-RRH-1177			
Air Conditioner (Laminar flow type)	HSS	Mbarara-RRH-1179			
Air Conditioner (Laminar flow type)	HSS	Mbarara-RRH-1181			
Air Conditioner (Laminar flow type)	HSS	Mbarara-RRH-1184			
Air Conditioner (Laminar flow type)	HSS	Mbarara-RRH-1187			
Air Conditioner (Laminar flow type)	HSS	Kiruhura-HC IV-27820			
Ambubag, Adult	ME	Rugazi-HC IV-28561			
Anaesthesia Machine with inbuilt medical oxygen generator	ME	Bwizibwera-HC IV-27533	9100C		MEI2110579
Anaesthesia Machine with inbuilt medical oxygen generator	ME	Mbarara-RRH-452		1009-9011-000	AMX0840663WA
Anaesthesia Cart	ME	Mbarara-RRH-425		9100C	ME12110532
Anaesthesia Cart	ME	Mbarara-RRH-465		9100C	ME12100468
Anaesthesia Cart	ME	Ishongororo-HC IV-27625		GE9100C	MEI2110607
Anaesthesia Cart	ME	Ruhoko-HC IV-27698		GE9100C	MEI211O506
Anaesthesia Cart	ME	Kabuyanda-HC IV-27859		9100C	MEI211O582
Anaesthesia Cart	ME	Kabuyanda-HC IV-27885	DATEX OHMEDA		1009-9011-000
Anaesthesia Cart	ME	Kazo-HC IV-27753		GE9100C	MEIZ100473

Equipment Name	Type	System ID	Model Name	Model No.	Serial No.
Anaesthesia Cart	ME	Kiruhura-HC IV-27822		GE9100C	MEI2110499
Anaesthesia Cart	ME	Kiruhura-HC IV-27828		9100C	10/17041
Anaesthesia Machine with a Monitor and Ventillator	ME	Nyamuyanja-HC IV-28011	9100C		ME12110548
Anaesthesia Machine with a Monitor and Ventillator	ME	Rugazi-HC IV-28896	9100C		ME12110510
Anaesthesia Machine with a Monitor and Ventillator	ME	Shuuku-HC IV-29750			10117050
Anaesthesia Unit and Ventillator	ME	Bugamba-HC IV-32327		9100C	ME12110507
Anaesthesia Unit and Ventillator	ME	Shuuku-HC IV-29514	9100c		ME12110569
Anaesthesia Unit and Ventillator	ME	Kyabugimbi-HC IV-30349		9100C	ME12110545
Analyzer CD4 (PIMA) count Machine	ME	Ishongororo-HC IV-27611			NAT-04005082
Analyzer CD4 (PIMA) count Machine	ME	Ishongororo-HC IV-27612		UG-0472	PIMA -D-008033
Analyzer CD4 (PIMA) count Machine	ME	Kabuyanda-HC III-27989			PIMA -D-00788
Analyzer CD4 (PIMA) count Machine	ME	Kazo-HC IV-27734			PIMA -D-008068
Analyzer CD4 (PIMA) count Machine	ME	Kiruhura-HC IV-27818	M-PIMA		NAT-04005115
Analyzer CD4 (PIMA) count Machine	ME	Bugamba-HC IV-32397	PIMA ANALYZR		Pima-A-D-003603
Analyzer CD4 (PIMA) count Machine	ME	Bugamba-HC IV-32403	PIMA ANALYZR		PIMA-D-005502
Analyzer CD4 (PIMA) count Machine	ME	Kabwohe-HC IV-29943	PIMA ANALYZR		PIMA-D-008224
Analyzer CD4 (PIMA) count Machine	ME	Bwizibwera-HC IV-27548			PIMA -D-005225
Analyzer CD4 (PIMA) count Machine	ME	Bwizibwera-HC IV-27559			NAT-04002308

Equipment Name	Type	System ID	Model Name	Model No.	Serial No.
Analyzer, CD4		Kyabugimbi-HC IV-29550	PIMA ANALYSER		PMA-D-002840
Analyzer, Heamatology		Bugamba-HC IV-32395			07317
Analyzer, Heamatology		Kabwohe-HC IV-29941		60 Ts	920246
Analyzer, Heamatology		Kabwohe-HC IV-29942	XQ-320		11110
Analyzer, Heamatology		Mbarara-RRH-1091	GF	GF 300	1A10001602
Analyzer, Heamatology		Mbarara-RRH-1092	MINDRAY	BC-5600	RX-39001168
Analyzer, Heamatology		Mbarara-RRH-1093	CD RUBY	ABBOTT	
Analyzer, Heamatology		Mbarara-RRH-1094	CD RUBY	ABBOTT	
Analyzer, Heamatology		Mbarara-RRH-1095	SYSMEX	XS1000i	69651
Analyzer, Heamatology		Mbarara-RRH-1096	SYSMEX XN 550	XN 550	20000
Analyzer, Heamatology		Mbarara-RRH-1097	SYSMEX XN 550	XN 550	20000
Analyzer, Heamatology		Mbarara-RRH-1098	SYSMEX XN 550	XN 550	20000
Analyzer, Heamatology		Mbarara-RRH-1099	SYSMEX XN 550	XN 550	20000
Analyzer, Heamatology		Mbarara-RRH-1100	SYSMEX XN 550	XN 550	20000
Analyzer, Heamatology		Mbarara-RRH-1101	SYSMEX XN 550	XN 550	20000
Analyzer, Heamatology		Mbarara-RRH-1102	SYSMEX XN 550	XN 550	20000
Analyzer, Heamatology		Mbarara-RRH-1103	SYSMEX XN 550	XN 550	20000
Analyzer, Heamatology		Mbarara-RRH-1104	SYSMEX XN 550	XN 550	20000
Analyzer, Heamatology		Mbarara-RRH-1105	SYSMEX XN 550	XN 550	20000
Analyzer, Heamatology		Mbarara-RRH-1106	SYSMEX XN 550	XN 550	20000
Analyzer, PCR System (DNA)	ME	Bugamba-HC IV-32396	mPIMA Analyzer		NAT-04002091
Analyzer, PCR System (DNA)	ME	Kabwohe-HC IV-29944	mPIMA		NAT-04005171
Analyzer, PCR System (DNA)	ME	Kabwohe-HC IV-29945	mPima		NAT-04002284
Analyzer, TB		Kabwohe-HC IV-29946	GXIV		816662
Analyzer, TB		Kabuyanda-HC IV-28008		GX-IVR2	8058001
Urine Analyser	ME	Mbarara-RRH-1115		GF-U28	
Arterial Blood	ME	Mbarara-	RAPID POINT 500e		162436

Equipment Name	Type	System ID	Model Name	Model No.	Serial No.
Gas (ABG) Machine		RRH-394			
Audiometer, Clinical	ME	Mbarara-RRH-1437		709-C	835LG
Autoclave, Electric 100 Liters	ME	Bwizibwera-HC IV-27540	LS-75HJ		16L-0972
Autoclave, Electric 100 Liters	ME	Mbarara-RRH-514		25X-2	0002515
Autoclave, Electric 100 Liters	ME	Kyabugimbi-HC IV-30358		3870MLV	2705700
Autoclave, Electric, 20 Liters	ME	Kyabugimbi-HC IV-29537			
Autoclave, Electric, 20 Liters	ME	Kyabugimbi-HC IV-30103	YXQG02		
Autoclave, Electric, 20 Liters	ME	Kyabugimbi-HC IV-30110		2340M	2605764
Autoclave, Electric, 20 Liters	ME	Bushenyi-HC IV-29011	BKM-P18(B)		1901043
Autoclave, Electric, 20 Liters	ME	Bushenyi-HC IV-29012			
Autoclave, Electric, 20 Liters	ME	Bushenyi-HC IV-29471	5OX		00003999
Autoclave, Electric, 20 Liters	ME	Ishongororo-HC IV-27569			
Autoclave, Electric, 20 Liters	ME	Ishongororo-HC IV-27657	MLV		
Autoclave, Electric, 20 Liters	ME	Ishongororo-HC IV-27662			
Autoclave, Electric, 20 Liters	ME	Ruhoko-HC IV-27691		75X	10007315
Autoclave, Electric, 20 Liters	ME	Ruhoko-HC IV-27703	PJ EXPORTS		133
Autoclave, Electric, 20 Liters	ME	Ruhoko-HC IV-27704	YXQG02		
Autoclave, Electric, 20 Liters	ME	Ruhoko-HC IV-27705			
Autoclave, Electric, 20 Liters	ME	Ruhoko-HC IV-27706			

Equipment Name	Type	System ID	Model Name	Model No.	Serial No.
Autoclave, Electric, 20 Liters	ME	Kabuyanda-HC IV-27930		XYQG02	
Autoclave, Electric, 20 Liters	ME	Kabuyanda-HC III-27864		3850E2	16070946
Autoclave, Electric, 20 Liters	ME	Kazo-HC IV-27755		YXQD02	
Autoclave, Electric, 20 Liters	ME	Kazo-HC IV-27763		2540E	1003123
Autoclave, Electric, 20 Liters	ME	Bugamba-HC IV-30431	YXQG02		
Autoclave, Electric, 20 Liters	ME	Bugamba-HC IV-32390		2540E	1003119
Autoclave, Electric, 20 Liters	ME	Rugazi-HC IV-28910	YXQGO2		
Autoclave, Electric, 20 Liters	ME	Rugazi-HC IV-28935	CS-B50L		722
Autoclave, Electric, 20 Liters	ME	Kiruhura-HC IV-27832			
Autoclave, Electric, 20 Liters	ME	Kiruhura-HC IV-27834			
Autoclave, Electric, 20 Liters	ME	Bwizibwera-HC IV-27510			
Autoclave, Electric, 40 Litres	ME	Bwizibwera-HC IV-27509		XXQG02	
Autoclave, Electric, 40 Litres	ME	Ishongororo-HC IV-27629		3870 MLV	2503701
Autoclave, Electric, 40 Litres	ME	Ishongororo-HC IV-27630		RUHS12	6082
Autoclave, Electric, 40 Litres	ME	Ishongororo-HC IV-27637		2340M	2505807
Autoclave, Electric, 40 Litres	ME	Kabuyanda-HC IV-27866		1941x	0002148
Autoclave, Electric, 40 Litres	ME	Kabuyanda-HC IV-27976		AE-75Dry	17798
Autoclave, Electric, 40 Litres	ME	Kazo-HC IV-27756			
Autoclave,	ME	Kazo-HC		PJ-EXPORT	145

Equipment Name	Type	System ID	Model Name	Model No.	Serial No.
Electric, 40 Litres		IV-27757			
Autoclave, Electric, 40 Litres	ME	Kiruhura-HC IV-27830		YX-24LM	205-15671
Autoclave, Electric, 40 Litres	ME	Kiruhura-HC IV-27831	UMCLAVE	8840X60	740
Autoclave, Electric, 570 Liters	ME	Mbarara-RRH-1113	PANASONIC	MLS	PE280063
Autoclave, Electric, 60 Liters	ME	Bushenyi-HC IV-29007	387EAD		13021921
Autoclave, Electric, 60 Liters	ME	Bugamba-HC IV-32389	Uniclave 88		741
Autoclave, Electric, 60 Liters	ME	Shuuku-HC IV-29532	Uniclave 88 (75 ltrs)		738
Autoclave, External Heated, 20 Litres	ME	Bugamba-HC IV-32391	WN1415MN		101107
Autoclave, External Heated, 20 Litres	ME	Rugazi-HC IV-28914			
Autoclave, External Heated, 20 Litres	ME	Shuuku-HC IV-29533	2540E		1003124
Autoclave, External Heated, 20 Litres	ME	Shuuku-HC IV-29535			
Autoclave, External Heated, 20 Litres	ME	Kyabugimbi-HC IV-29528		YX-280A	146
Automated Blood Culture Machine	ME	Mbarara-RRH-1144	BD	BACTEC9050	NB6102
Automated Blood Culture Machine	ME	Mbarara-RRH-1145	BD	BACTEC FX	FT9458
Automated Coagulation Analyzer	ME	Mbarara-RRH-1114		RT-2202	561101006E
Automated Coagulation Analyzer	ME	Kabuyanda-HC IV-27908		HTU-110	001839
Automated External Defibrillator (AED)	ME	Mbarara-RRH-393			1510-051
Automatic Bacterial Identification Machine	ME	Mbarara-RRH-963		BDBACTECFX40	FF7554

Equipment Name	Type	System ID	Model Name	Model No.	Serial No.
Automatic Bacterial Identification Machine	ME	Mbarara-RRH-964		BDBACTECFX40	FF7554
Automatic Bacterial Identification Machine	ME	Mbarara-RRH-965		BDBACTECFX40	FF7554
Automatic Pipette, Adjustable	ME	Mbarara-RRH-1116	Finnpipette		OH48776
Automatic Pipette, Adjustable	ME	Mbarara-RRH-1117	Finnpipette		OH48776
Automatic Pipette, Adjustable	ME	Mbarara-RRH-1119	Finnpipette		OH48281
Automatic Pipette, Adjustable	ME	Mbarara-RRH-1121	Finnpipette		OH67360
Automatic Pipette, Adjustable	ME	Mbarara-RRH-1124	Finnpipette		OH53405
Automatic Pipette, Adjustable	ME	Mbarara-RRH-1126	Brand		
Automatic Pipette, Adjustable	ME	Mbarara-RRH-1128	Optipette		356062132
Automatic Pipette, Adjustable	ME	Mbarara-RRH-1135	Volac		
Automatic Pipette, Adjustable	ME	Mbarara-RRH-1138			
Automatic Pipette, Adjustable	ME	Mbarara-RRH-1141			V83687
Automatic Pipette, Adjustable	ME	Mbarara-RRH-1143			3130
Automatic Pipette, Adjustable	ME	Mbarara-RRH-1151	Assistant		O5H15326
Automatic Pipette, Adjustable	ME	Mbarara-RRH-1153	Reflotron		10112
Automatic Pipette, Adjustable	ME	Mbarara-RRH-1155	Gilson		069656K
Automatic Pipette, Adjustable	ME	Mbarara-RRH-1158	Gilson		050814N
Automatic Pipette, Adjustable	ME	Mbarara-RRH-1160	Gilson		R54787J

Equipment Name	Type	System ID	Model Name	Model No.	Serial No.
Automatic Pipette, Adjustable	ME	Mbarara-RRH-1172	Finnpipette		OH07842
Automatic Pipette, Adjustable	ME	Mbarara-RRH-1173	Finnpipette		V70439
Blood Pressure Machine Anaeroid, Paediatric	ME	Kyabugimbi-HC IV-30123			
Blood Pressure Machine Anaeroid, Paediatric	ME	Kyabugimbi-HC IV-30350			
Blood Pressure Machine Anaeroid, Paediatric	ME	Kabwohe-HC IV-30069			
Blood Pressure Machine Anaeroid, Paediatric	ME	Kabwohe-HC IV-30070			
Blood Pressure Machine Anaeroid, Paediatric	ME	Kabwohe-HC IV-30071			
Blood Pressure Machine Anaeroid, Paediatric	ME	Kabwohe-HC IV-30072			
Blood Pressure Machine, Digital, Adult	ME	Bugamba-HC IV-30430			
Blood Pressure Machine, Digital, Adult	ME	Bugamba-HC IV-30432		BA-801	
Blood Pressure Machine, Digital, Adult	ME	Bugamba-HC IV-30445	BP-103H		18031258
Blood Pressure Machine, Digital, Adult	ME	Kabwohe-HC IV-29809		M2	20170108513VG
Blood Pressure Machine, Digital, Adult	ME	Kabwohe-HC IV-29832			2103200302030588
Blood Pressure Machine, Digital, Adult	ME	Kabwohe-HC IV-29922	M2		202102001865V
Blood Pressure Machine, Digital, Adult	ME	Kabwohe-HC IV-29923	M3		20180808882VG
Blood Pressure Machine, Digital, Adult	ME	Kabwohe-HC IV-29924	BA-801		
Blood Pressure Machine, Digital,	ME	Kabwohe-HC IV-29926	ba-801		

Equipment Name	Type	System ID	Model Name	Model No.	Serial No.
Adult					
Baby Cot	MF	Kyabugimbi-HC IV-30335			
Baby Cot	MF	Kyabugimbi-HC IV-30336			
Baby Cot	MF	Nyamuyanja-HC IV-28020			
Baby Cot	MF	Kabuyanda-HC IV-27961			
Baby Cot	MF	Kabuyanda-HC IV-27975			
Baby Incubator	ME	Nyamuyanja-HC IV-28024		YP-100	01121203046
Baby Incubator	ME	Kabuyanda-HC IV-27950		YP-100	01071003054
Baby Incubator	ME	Kabuyanda-HC IV-27954		YP-100	011212024042
Baby Incubator	ME	Kabuyanda-HC IV-27970		YP-100	01121203071
Baby Incubator	ME	Kiruhura-HC IV-27840		B/100	2180710153
Baby Incubator	ME	Mbarara-RRH-558			C3980090
Baby Incubator	ME	Rugazi-HC IV-28565			
Balance, Organs Weighing		Kabuyanda-HC IV-27993		JA2003	SHP0700314072
Hospital Bed, Adult	MF	Kyabugimbi-HC IV-29541			
Hospital Bed, Adult	MF	Kyabugimbi-HC IV-30152			
Hospital Bed, Adult	MF	Kyabugimbi-HC IV-30153			
Hospital Bed, Adult	MF	Kyabugimbi-HC IV-30156			
Hospital Bed, Adult	MF	Kyabugimbi-HC IV-30158			
Hospital Bed, Adult	MF	Kyabugimbi-HC IV-30159			
Hospital Bed, Adult	MF	Kyabugimbi-HC IV-30162			
Hospital Bed, Adult	MF	Kyabugimbi-HC IV-30164			
Hospital Bed, Adult	MF	Kyabugimbi-HC IV-30165			
Hospital Bed, Adult	MF	Kyabugimbi-HC IV-30166			
Hospital Bed, Adult	MF	Kyabugimbi-HC IV-30168			
Hospital Bed, Adult	MF	Kyabugimbi-HC IV-30373			
Hospital Bed, Adult	MF	Kyabugimbi-HC IV-30374			

Equipment Name	Type	System ID	Model Name	Model No.	Serial No.
Hospital Bed, Adult	MF	Kyabugimbi-HC IV-30375			
Hospital Bed, Adult	MF	Kyabugimbi-HC IV-30376			
Hospital Bed, Adult	MF	Kyabugimbi-HC IV-30377			
Hospital Bed, Adult	MF	Kyabugimbi-HC IV-30378			
Hospital Bed, Adult	MF	Kyabugimbi-HC IV-30379			
Hospital Bed, Adult	MF	Kyabugimbi-HC IV-30380			
Hospital Bed, Adult	MF	Kyabugimbi-HC IV-30381			
Hospital Bed, Adult	MF	Kyabugimbi-HC IV-30382			
Hospital Bed, Adult	MF	Kyabugimbi-HC IV-30383			
Hospital Bed, Adult	MF	Kyabugimbi-HC IV-30384			
Hospital Bed, Adult	MF	Kyabugimbi-HC IV-30385			
Hospital Bed, Adult	MF	Kyabugimbi-HC IV-30386			
Hospital Bed, Adult	MF	Kyabugimbi-HC IV-30387			
Hospital Bed, Adult	MF	Kyabugimbi-HC IV-30388			
Hospital Bed, Adult	MF	Kyabugimbi-HC IV-30389			
Hospital Bed, Adult	MF	Kyabugimbi-HC IV-30390			
Hospital Bed, Adult	MF	Kyabugimbi-HC IV-30391			
Hospital Bed, Adult	MF	Kyabugimbi-HC IV-30392			
Hospital Bed, Adult	MF	Kyabugimbi-HC IV-30393			
Hospital Bed, Adult	MF	Kyabugimbi-HC IV-30394			
Hospital Bed, Adult	MF	Kyabugimbi-HC IV-30400			
Hospital Bed, Adult	MF	Kyabugimbi-HC IV-30401			
Hospital Bed, Adult	MF	Kyabugimbi-HC IV-30402			
Hospital Bed, Adult	MF	Kyabugimbi-HC IV-30403			
Hospital Bed, Adult	MF	Kyabugimbi-HC IV-30404			
Hospital Bed, Adult	MF	Kyabugimbi-HC IV-30405			
Hospital Bed,	MF	Kyabugimbi-			

Equipment Name	Type	System ID	Model Name	Model No.	Serial No.
Adult		HC IV-30406			
Hospital Bed, Adult	MF	Kyabugimbi-HC IV-30407			
Hospital Bed, Adult	MF	Kyabugimbi-HC IV-30411			
Hospital Bed, Adult	MF	Kyabugimbi-HC IV-30413			
Hospital Bed, Adult	MF	Kyabugimbi-HC IV-30414			
Hospital Bed, Adult	MF	Kyabugimbi-HC IV-30415			
Hospital Bed, Adult	MF	Kyabugimbi-HC IV-30416			
Hospital Bed, Adult	MF	Kyabugimbi-HC IV-30417			
Hospital Bed, Adult	MF	Kyabugimbi-HC IV-30418			
Hospital Bed, Adult	MF	Kyabugimbi-HC IV-30419			
Hospital Bed, Adult	MF	Kyabugimbi-HC IV-30420			
Hospital Bed, Adult	MF	Kyabugimbi-HC IV-30421			
Hospital Bed, Adult	MF	Bushenyi-HC IV-29016			
Hospital Bed, Adult	MF	Ishongororo-HC IV-27632			
Hospital Bed, Adult	MF	Ishongororo-HC IV-27633			
Hospital Bed, Adult	MF	Ishongororo-HC IV-27634			
Hospital Bed, Adult	MF	Ishongororo-HC IV-27635			
Hospital Bed, Adult	MF	Ishongororo-HC IV-27636			
Hospital Bed, Adult	MF	Ishongororo-HC IV-27640			
Hospital Bed, Adult	MF	Ishongororo-HC IV-27641			
Hospital Bed, Adult	MF	Ishongororo-HC IV-27643			
Hospital Bed, Adult	MF	Ishongororo-HC IV-27645			
Hospital Bed, Adult	MF	Ishongororo-HC IV-27646			
Hospital Bed, Adult	MF	Ishongororo-HC IV-27648			
Hospital Bed, Adult	MF	Ishongororo-HC IV-27649			
Hospital Bed, Adult	MF	Ishongororo-HC IV-27651			
Hospital Bed, Adult	MF	Ishongororo-HC IV-27652			

Equipment Name	Type	System ID	Model Name	Model No.	Serial No.
Hospital Bed, Adult	MF	Ishongororo-HC IV-27653			
Hospital Bed, Adult	MF	Ruhoko-HC IV-27681			
Hospital Bed, Adult	MF	Ruhoko-HC IV-27685			
Hospital Bed, Adult	MF	Ruhoko-HC IV-27711			
Hospital Bed, Adult	MF	Nyamuyanja-HC IV-28025			
Hospital Bed, Adult	MF	Nyamuyanja-HC IV-28033			
Hospital Bed, Adult	MF	Isibuka-HC III-27647			
Hospital Bed, Adult	MF	Kabuyanda-HC IV-27940			
Hospital Bed, Adult	MF	Kabuyanda-HC IV-27942			
Hospital Bed, Adult	MF	Kabuyanda-HC IV-27947			
Hospital Bed, Adult	MF	Kabuyanda-HC IV-27949			
Hospital Bed, Adult	MF	Kazo-HC IV-27739			
Hospital Bed, Adult	MF	Kazo-HC IV-27745			
Hospital Bed, Adult	MF	Kazo-HC IV-27749			
Hospital Bed, Adult	MF	Kiruhura-HC IV-27825		3008AB	
Hospital Bed, Adult	MF	Mbarara-RRH-2774			
Hospital Bed, Adult	MF	Bugamba-HC IV-30433			
Hospital Bed, Adult	MF	Bugamba-HC IV-30434			
Hospital Bed, Adult	MF	Bugamba-HC IV-30435			
Hospital Bed, Adult	MF	Bugamba-HC IV-30436			
Hospital Bed, Adult	MF	Bugamba-HC IV-30437			
Hospital Bed, Adult	MF	Bugamba-HC IV-30438			
Hospital Bed, Adult	MF	Bugamba-HC IV-30439			
Hospital Bed, Adult	MF	Bugamba-HC IV-30440			
Hospital Bed, Adult	MF	Bugamba-HC IV-30444			
Hospital Bed, Adult	MF	Bugamba-HC IV-32392			
Hospital Bed,	MF	Bugamba-			

Equipment Name	Type	System ID	Model Name	Model No.	Serial No.
Adult		HC IV-32416			
Hospital Bed, Adult	MF	Bugamba-HC IV-32417			
Hospital Bed, Adult	MF	Bugamba-HC IV-32418			
Hospital Bed, Adult	MF	Bugamba-HC IV-32419			
Hospital Bed, Adult	MF	Bugamba-HC IV-32423			
Hospital Bed, Adult	MF	Bugamba-HC IV-32424			
Hospital Bed, Adult	MF	Bugamba-HC IV-32425			
Hospital Bed, Adult	MF	Bugamba-HC IV-32426			
Hospital Bed, Adult	MF	Bugamba-HC IV-32428			
Hospital Bed, Adult	MF	Bugamba-HC IV-32429			
Hospital Bed, Adult	MF	Bugamba-HC IV-32430			
Hospital Bed, Adult	MF	Bugamba-HC IV-32431			
Hospital Bed, Adult	MF	Rugazi-HC IV-28551			
Hospital Bed, Adult	MF	Rugazi-HC IV-28918			
Hospital Bed, Adult	MF	Rugazi-HC IV-28926			
Hospital Bed, Adult	MF	Rugazi-HC IV-28929			
Hospital Bed, Adult	MF	Rugazi-HC IV-28933			
Hospital Bed, Adult	MF	Kabwohe-HC IV-29833			
Hospital Bed, Adult	MF	Kabwohe-HC IV-29834			
Hospital Bed, Adult	MF	Kabwohe-HC IV-29835			
Hospital Bed, Adult	MF	Kabwohe-HC IV-29836			
Hospital Bed, Adult	MF	Kabwohe-HC IV-29837			
Hospital Bed, Adult	MF	Kabwohe-HC IV-29838			
Hospital Bed, Adult	MF	Kabwohe-HC IV-29839			
Hospital Bed, Adult	MF	Kabwohe-HC IV-29840			
Hospital Bed, Adult	MF	Kabwohe-HC IV-29843			
Hospital Bed, Adult	MF	Kabwohe-HC IV-29844			

Equipment Name	Type	System ID	Model Name	Model No.	Serial No.
Hospital Bed, Adult	MF	Kabwohe-HC IV-29846			
Hospital Bed, Adult	MF	Kabwohe-HC IV-29847			
Hospital Bed, Adult	MF	Kabwohe-HC IV-29849			
Hospital Bed, Adult	MF	Kabwohe-HC IV-29850			
Hospital Bed, Adult	MF	Kabwohe-HC IV-29852			
Hospital Bed, Adult	MF	Kabwohe-HC IV-29853			
Hospital Bed, Adult	MF	Kabwohe-HC IV-29854			
Hospital Bed, Adult	MF	Kabwohe-HC IV-29856			
Hospital Bed, Adult	MF	Kabwohe-HC IV-29927			
Hospital Bed, Adult	MF	Kabwohe-HC IV-29928			
Hospital Bed, Adult	MF	Kabwohe-HC IV-29929			
Hospital Bed, Adult	MF	Kabwohe-HC IV-29930			
Hospital Bed, Adult	MF	Kabwohe-HC IV-29931			
Hospital Bed, Adult	MF	Kabwohe-HC IV-29932			
Hospital Bed, Adult	MF	Kabwohe-HC IV-29933			
Hospital Bed, Adult	MF	Kabwohe-HC IV-29934			
Hospital Bed, Adult	MF	Kabwohe-HC IV-29935			
Hospital Bed, Adult	MF	Kabwohe-HC IV-29936			
Hospital Bed, Adult	MF	Kabwohe-HC IV-29938			
Hospital Bed, Adult	MF	Shuuku-HC IV-29495			
Hospital Bed, Adult	MF	Shuuku-HC IV-29496			
Hospital Bed, Adult	MF	Shuuku-HC IV-29497			
Hospital Bed, Adult	MF	Shuuku-HC IV-29499			
Hospital Bed, Adult	MF	Shuuku-HC IV-29501			
Hospital Bed, Adult	MF	Shuuku-HC IV-29539			
Hospital Bed, Adult	MF	Shuuku-HC IV-30529			
Hospital Bed,	MF	Kiruhura-HC			

Equipment Name	Type	System ID	Model Name	Model No.	Serial No.
Adult		IV-27842			
Hospital Bed, Adult	MF	Bwizibwera-HC IV-27535			
Hospital Bed, Adult	MF	Bwizibwera-HC IV-27536			
Hospital Bed, Adult	MF	Bwizibwera-HC IV-27551			
Hospital Bed, Adult	MF	Bwizibwera-HC IV-27556			
Hospital Bed, Adult	MF	Mbarara-RRH-484		2011061407020-016	
Bed, Children, Patient	MF	Mbarara-RRH-385		NTCR-SD05	NTCRSD05
Bed, Children, Patient	MF	Mbarara-RRH-509			
Bed, Children, Patient	MF	Mbarara-RRH-510			
Bed, Children, Patient	MF	Mbarara-RRH-529			
Bed, Children, Patient	MF	Kyabugimbi-HC IV-30337			
Bed, Children, Patient	MF	Kabwohe-HC IV-29857			
Bed, Children, Patient	MF	Kabwohe-HC IV-29859			
Bed, Children, Patient	MF	Kabwohe-HC IV-29861			
Bed, Children, Patient	MF	Shuuku-HC IV-30531			
Operation Bed, Electric	MF	Mbarara-RRH-34100		31250A+1125004A	W/O#00206544-0040
Operation Bed, Electric	MF	Mbarara-RRH-34101		31250A+1125004A	W/O#00206544-0034
Operation Bed, Electric	MF	Mbarara-RRH-34102		31250A+1125004A	W/O#00206544-0030
Operation Bed, Electric	MF	Mbarara-RRH-375	HBWO-H-5-75-25A		
Biological Safety Cabinet		Bwizibwera-HC IV-27558		AC2-4EI	2007-20698
Biosafety Cabinet Class II	ME	Mbarara-RRH-766	AIRTECH	BSC-100311A2	JT21070601
Biosafety Cabinet Class II	ME	Mbarara-RRH-767	AIRTECH	BSC-100311A2	JT21070601
Biosafety Cabinet Class II	ME	Mbarara-RRH-768	AIRTECH	BSC-100311A2	JT21070601
Biosafety Cabinet Class II	ME	Mbarara-RRH-769	AIRTECH	BSC-100311A2	JT21070601
Biosafety Cabinet Class II	ME	Mbarara-RRH-770	AIRTECH	BSC-100311A2	JT21070601
Biosafety Cabinet Class II	ME	Mbarara-RRH-771	AIRTECH	BSC-100311A2	JT21070601
Biosafety Cabinet Class II	ME	Mbarara-RRH-772	AIRTECH	BSC-100311A2	JT21070601

Equipment Name	Type	System ID	Model Name	Model No.	Serial No.
Biosafety Cabinet Class II	ME	Mbarara-RRH-773	AIRTECH	BSC-100311A2	JT21070601
Biosafety Cabinet Class II	ME	Mbarara-RRH-774	AIRTECH	BSC-100311A2	JT21070601
Biosafety Cabinet Class II	ME	Mbarara-RRH-775	AIRTECH	BSC-100311A2	JT21070601
Biosafety Cabinet Class II	ME	Mbarara-RRH-776	AIRTECH	BSC-100311A2	JT21070601
Biosafety Cabinet Class II	ME	Mbarara-RRH-777	AIRTECH	BSC-100311A2	JT21070601
Biosafety Cabinet Class II	ME	Mbarara-RRH-778	AIRTECH	BSC-100311A2	JT21070601
Biosafety Cabinet Class II	ME	Mbarara-RRH-779	AIRTECH	BSC-100311A2	JT21070601
Biosafety Cabinet Class II	ME	Mbarara-RRH-780	AIRTECH	BSC-100311A2	JT21070601
Biosafety Cabinet Class II	ME	Mbarara-RRH-781	AIRTECH	BSC-100311A2	JT21070601
Biosafety Cabinet Class II	ME	Mbarara-RRH-966	AIRTECH	BSC-100311A2	JT21070605
Biosafety Cabinet Class II	ME	Mbarara-RRH-967	AIRTECH	BSC-100311A2	JT21070605
Biosafety Cabinet Class II	ME	Mbarara-RRH-968	AIRTECH	BSC-100311A2	JT21070605
Biosafety Cabinet Class II	ME	Mbarara-RRH-982	AIRTECH	BSC-100311A2	JT21070603
Biosafety Cabinet Class II	ME	Mbarara-RRH-983	AIRTECH	BSC-100311A2	JT21070604
Biosafety Cabinet Class II	ME	Mbarara-RRH-1107	ESCO CLASS 11	AC2-4E1	2006-15385
Biosafety Cabinet Class II	ME	Mbarara-RRH-1108	GRANT	UVT-B-AR	04010920030068
Biosafety Cabinet Class II	ME	Mbarara-RRH-1109	GRANT	UVT-B-AR	04010920030068
Biosafety Cabinet Class II	ME	Mbarara-RRH-1110	THERMOSCIENTIFIC	MSC advantange	42567883
Biosafety Cabinet Class II	ME	Mbarara-RRH-1251	AIRTECH	BSC-1100311A2	JT21070602
Biosafety Cabinet Class II	ME	Bushenyi-HC IV-29027	msc advantage		42969800
Blood Gas Analyser	ME	Mbarara-RRH-1060			(21)420039
Blood Grouping Analyzer	ME	Mbarara-RRH-1146	ECHO LUMENA	echo lumena	M20940
Blood Warmer	ME	Kabuyanda-HC IV-27946	14103055	FD0902	
BP Machine, Mercury		Nyamiyaga-HC II-28042		BP-102M	16236303
BP Machine, Mercury		Nyamuyanja-HC IV-28037	GITTOES		
BP Machine, Mercury		Nyamuyanja-HC IV-28044		BP-102M	16236303
BP Machine,		Kabuyanda-			

Equipment Name	Type	System ID	Model Name	Model No.	Serial No.
Mercury		HC IV-27878			
BP Machine, Mercury		Kabuyanda-HC IV-27903		024	1001071827
BP Machine, Mercury		Bwizibwera-HC IV-27543	BA-823		
BP Machine, Mercury		Rugazi-HC IV-28562	LD581(LD3)		SLD1900315836
BP Machine, Mercury		Rugazi-HC IV-28563	BA-823		
BP Machine, Mercury		Rugazi-HC IV-28915			
BP Machine, Mercury		Rugazi-HC IV-28924			
BP Machine, Mercury		Kabwohe-HC IV-29954			
BP Machine, Mercury		Shuuku-HC IV-29509			
BP Machine, Mercury		Shuuku-HC IV-29511	M2ECO(HEM-7120-AF)		20180704468VG
BP Machine, Mercury		Shuuku-HC IV-30533			
BP Machine, Mercury		Shuuku-HC IV-30534			
Burn Out Machine (Oven)	ME	Bushenyi-HC IV-29008	DHG-9025A		211130057
Photographic camera	HSS	Mbarara-RRH-758	DELL		
CD4 Count Machine	ME	Bushenyi-HC IV-29473	Pima Analyser		Pima-D-008209
CD4 Count Machine	ME	Ruhoko-HC IV-27676	BD-FACS PRESTO		R8074479
CD4 Count Machine	ME	Mbarara-RRH-1118	Alere	Pima	D-006087
CD4 Count Machine	ME	Mbarara-RRH-1120	Alere	Pima	D-005043
CD4 Count Machine	ME	Mbarara-RRH-1122	Alere	Pima	D-006101
CD4 Count Machine	ME	Mbarara-RRH-1123	Alere	Pima	D-006101
CD4 Count Machine	ME	Mbarara-RRH-1125	Alere	Pima	D-006137
CD4 Count Machine	ME	Mbarara-RRH-1127	BD	FACSPresto	60632455
CD4 Count Machine	ME	Mbarara-RRH-1129	BD	FACSPresto	R312228
CD4 Count Machine	ME	Mbarara-RRH-1258	BD	FACSPresto	60632455
CD4 Count Machine, PIMA	ME	Ishongororo-HC IV-27610			NAT-04000680
CD4 Count Machine, PIMA	ME	Kabuyanda-HC IV-28004		NAT-04005020	
CD4 Count Machine, PIMA	ME	Kiruhura-HC IV-27813			PIMA/D/003446

Equipment Name	Type	System ID	Model Name	Model No.	Serial No.
Centrifuge, Manual	ME	Mbarara-RRH-519	ADAMS PHYSICIAN		R-13584
Centrifuge, Manual	ME	Mbarara-RRH-520	ADAMS DYNAC	CT-1300	AJ-4648
Centrifuge, Manual	ME	Mbarara-RRH-534	ADAMS PYSICIAN		13584
Centrifuge, Manual	ME	Mbarara-RRH-535	ADAMS DYNAC		AJ-4648
Centrifuge, Manual	ME	Mbarara-RRH-536	TDX		
Centrifuge, Manual	ME	Mbarara-RRH-1431			3178
Analyzer, Chemistry	ME	Mbarara-RRH-1079	HUMALYZER 2000	2500	2500-3724
Analyzer, Chemistry	ME	Mbarara-RRH-1081	HUMALYTE PLUS 5	PLUS 5	600018
Analyzer, Chemistry	ME	Mbarara-RRH-1082	HUMASTAR 200	16895	20150501002
Analyzer, Chemistry	ME	Mbarara-RRH-1083	HUMASTAR 200	16895	21191388003
Analyzer, Chemistry	ME	Mbarara-RRH-1085	ELISYS Uno	17350	2950-6099
Analyzer, Chemistry	ME	Mbarara-RRH-1086	HUMASTAR 180	16900	7631
Analyzer, Chemistry	ME	Mbarara-RRH-1088	FINCARE Wndfo	FS-113	FS1132112212558
Analyzer, Chemistry	ME	Mbarara-RRH-1089	HUMACLIA	150	IA10001602
Analyzer, Chemistry	ME	Mbarara-RRH-1246	HumaClia	150	1A10001602
Analyzer, Chemistry	ME	Mbarara-RRH-1255	Fincare TM	FS-112	FS1132012203240
Analyzer, Chemistry	ME	Bushenyi-HC IV-29477	Human 200		21220389004
Analyzer, Chemistry	ME	Ishongororo-HC IV-27658			TPV 21821
Analyzer, Chemistry	ME	Ishongororo-HC IV-27660			TLDU4809
Analyzer, Chemistry	ME	Ruhoko-HC IV-27677	HUMALYTE PLUS		1605760
Analyzer, Chemistry	ME	Ruhoko-HC IV-27678	HUMASTAR 100		11160324003
Analyzer, Chemistry	ME	Bwizibwera-HC IV-27566		XP-300	C1747
Colorimeter	ME	Mbarara-RRH-1036	COLOR WAVE	C.07000	1225
Colorimeter	ME	Mbarara-RRH-1037	SHERWOOD	254	14046
Colorimeter	ME	Mbarara-RRH-1039	WPA	C07000	1640
Computer Monitor		Mbarara-RRH-818	DELL	SUNON MAGLEV	CN-07CXPR-72872-727A66B-A0
Computer		Mbarara-	DELL	SUNON MAGLEV	CN-07CXPR-72872-727A66B-A0

Equipment Name	Type	System ID	Model Name	Model No.	Serial No.
Monitor		RRH-819			
Computer Monitor		Mbarara-RRH-820	DELL	SUNON MAGLEV	CN-07CXPR-72872-727A66B-A0[illegible]
Computer Monitor		Mbarara-RRH-821	DELL	SUNON MAGLEV	CN-07CXPR-72872-727A66B-A0[illegible]
Computer Monitor		Mbarara-RRH-822	DELL	SUNON MAGLEV	CN-07CXPR-72872-727A66B-A0[illegible]
Computer Monitor		Mbarara-RRH-823	DELL	SUNON MAGLEV	CN-07CXPR-72872-727A66B-A0[illegible]
Computer Monitor		Mbarara-RRH-824	DELL	SUNON MAGLEV	CN-07CXPR-72872-727A66B-A0[illegible]
Computer Monitor		Mbarara-RRH-825	DELL	SUNON MAGLEV	CN-07CXPR-72872-727A66B-A0[illegible]
Computer Monitor		Mbarara-RRH-826	DELL	SUNON MAGLEV	CN-07CXPR-72872-727A66B-A0[illegible]
Computer Monitor		Mbarara-RRH-827	DELL	SUNON MAGLEV	CN-07CXPR-72872-727A66B-A0[illegible]
Computer Monitor		Mbarara-RRH-828	DELL	SUNON MAGLEV	CN-07CXPR-72872-727A66B-A0[illegible]
Computer Monitor		Mbarara-RRH-829	DELL	SUNON MAGLEV	CN-07CXPR-72872-727A66B-A0[illegible]
Computer Monitor		Mbarara-RRH-830	DELL	SUNON MAGLEV	CN-07CXPR-72872-727A66B-A0[illegible]
Computer Monitor		Mbarara-RRH-831	DELL	SUNON MAGLEV	CN-07CXPR-72872-727A66B-A0[illegible]
Computer Monitor		Mbarara-RRH-832	DELL	SUNON MAGLEV	CN-07CXPR-72872-727A66B-A0[illegible]
Computer Monitor		Mbarara-RRH-833	DELL	SUNON MAGLEV	CN-07CXPR-72872-727A66B-A0[illegible]
Computer Monitor		Mbarara-RRH-834	DELL	SUNON MAGLEV	CN-07CXPR-72872-727A66B-A0[illegible]
Computer Monitor		Mbarara-RRH-835	DELL	SUNON MAGLEV	CN-07CXPR-72872-727A66B-A0[illegible]
Computer Monitor		Mbarara-RRH-836	DELL	SUNON MAGLEV	CN-07CXPR-72872-727A66B-A0[illegible]
Computer Monitor		Mbarara-RRH-837	DELL	SUNON MAGLEV	CN-07CXPR-72872-727A66B-A0[illegible]
Computer Monitor		Mbarara-RRH-917	DELL		CN-ONKOP9-FCOO-IBF-C8JB-AO[illegible]
Computer Monitor		Mbarara-RRH-918	DELL		CN-ONKOP9-FCOO-IBF-C8JB-AO[illegible]
Computer Monitor		Mbarara-RRH-919	DELL		CN-ONKOP9-FCOO-IBF-C8JB-AO[illegible]
Computer Monitor		Mbarara-RRH-920	DELL		CN-ONKOP9-FCOO-IBF-C8JB-AO[illegible]
Computer Monitor		Mbarara-RRH-921	DELL		CN-ONKOP9-FCOO-IBF-C8JB-AO[illegible]
Computer Monitor		Mbarara-RRH-922	DELL		CN-ONKOP9-FCOO-IBF-C8JB-AO[illegible]
Computer Monitor		Mbarara-RRH-923	DELL		CN-ONKOP9-FCOO-IBF-C8JB-AO[illegible]
Computer Monitor		Mbarara-RRH-924	DELL		CN-ONKOP9-FCOO-IBF-C8JB-AO[illegible]

Equipment Name	Type	System ID	Model Name	Model No.	Serial No.
Computer Monitor		Mbarara-RRH-925	DELL		CN-ONKOP9-FCOO-IBF-C8JB-AO
Computer Monitor		Mbarara-RRH-926	DELL		CN-ONKOP9-FCOO-IBF-C8JB-AO
Computer Monitor		Mbarara-RRH-927	DELL		CN-ONKOP9-FCOO-IBF-C8JB-AO
Computer Monitor		Mbarara-RRH-928	DELL		CN-ONKOP9-FCOO-IBF-C8JB-AO
Computer Monitor		Mbarara-RRH-929	DELL		CN-ONKOP9-FCOO-IBF-C8JB-AO
Computer Monitor		Mbarara-RRH-930	DELL		CN-ONKOP9-FCOO-IBF-C8JB-AO
Computer Monitor		Mbarara-RRH-931	DELL		CN-ONKOP9-FCOO-IBF-C8JB-AO
Computer Monitor		Mbarara-RRH-932	DELL		CN-ONKOP9-FCOO-IBF-C8JB-AO
Computer Monitor		Mbarara-RRH-933	DELL		CN-ONKOP9-FCOO-IBF-C8JB-AO
Computer Monitor		Mbarara-RRH-934	DELL		CN-ONKOP9-FCOO-IBF-C8JB-AO
Computer Monitor		Mbarara-RRH-935	DELL		CN-ONKOP9-FCOO-IBF-C8JB-AO
Computer Monitor		Mbarara-RRH-936	DELL		CN-ONKOP9-FCOO-IBF-C8JB-AO
Computer Monitor		Mbarara-RRH-937	DELL		CN-ONKOP9-FCOO-IBF-C8JB-AO
Computer Monitor		Mbarara-RRH-938	DELL		CN-ONKOP9-FCOO-IBF-C8JB-AO
Computer Monitor		Mbarara-RRH-939	DELL		CN-ONKOP9-FCOO-IBF-C8JB-AO
Computer Monitor		Mbarara-RRH-940	DELL		CN-ONKOP9-FCOO-IBF-C8JB-AO
Computer Monitor		Mbarara-RRH-941	DELL		CN-ONKOP9-FCOO-IBF-C8JB-AO
Computer Monitor		Mbarara-RRH-942	DELL		CN-ONKOP9-FCOO-IBF-C8JB-AO
Computer Monitor		Mbarara-RRH-943	DELL		CN-ONKOP9-FCOO-IBF-C8JB-AO
Computer Monitor		Mbarara-RRH-944	DELL		CN-ONKOP9-FCOO-IBF-C8JB-AO
Computer Monitor		Mbarara-RRH-945	DELL		CN-ONKOP9-FCOO-IBF-C8JB-AO
Computer Monitor		Mbarara-RRH-946	DELL		CN-ONKOP9-FCOO-IBF-C8JB-AO
Computer Monitor		Mbarara-RRH-947	DELL		CN-ONKOP9-FCOO-IBF-C8JB-AO
Computer Monitor		Mbarara-RRH-948	DELL		CN-ONKOP9-FCOO-IBF-C8JB-AO
Computer Monitor		Mbarara-RRH-949	DELL		CN-ONKOP9-FCOO-IBF-C8JB-AO
Computer Monitor		Mbarara-RRH-950	DELL		CN-ONKOP9-FCOO-IBF-C8JB-AO
Computer		Mbarara-	DELL		CN-ONKOP9-FCOO-IBF-C8JB-AO

Equipment Name	Type	System ID	Model Name	Model No.	Serial No.
Monitor		RRH-951			
Computer Monitor		Mbarara-RRH-952	DELL		CN-ONKOP9-FCOO-IBF-C8JB-AO[illegible]
Computer Monitor		Mbarara-RRH-953	DELL		CN-ONKOP9-FCOO-IBF-C8JB-AO[illegible]
Computer Monitor		Mbarara-RRH-954	DELL		CN-ONKOP9-FCOO-IBF-C8JB-AO[illegible]
Computer Monitor		Mbarara-RRH-955	DELL		CN-ONKOP9-FCOO-IBF-C8JB-AO[illegible]
Computer Monitor		Mbarara-RRH-956	DELL		CN-ONKOP9-FCOO-IBF-C8JB-AO[illegible]
Computer Monitor		Mbarara-RRH-1080			CNC9522NNC
Computer Monitor		Mbarara-RRH-1433	HP		CNC90726MP
Computer Monitor		Bwizibwera-HC IV-27515	DEL		CN-OF7FOW-FCCOO-OCO-ACL1-[illegible]
Computer Monitor		Bwizibwera-HC IV-27516	DEL		CN-OF7FOW-FCCOO-OCO-ACL1-[illegible]
Computer Monitor		Bwizibwera-HC IV-27519	DEL		CNC709Q25N
Computer Monitor		Bwizibwera-HC IV-27526	DEL		S/N:CN-ONH7J1/FCCOO-18GC6[illegible]
Computer Monitor		Bwizibwera-HC IV-27528	DEL		SN:CN-ONH7J1-FCC00-18G-D3F[illegible]
Computer Monitor		Bwizibwera-HC IV-27531	DEL		S/N:CN-ONH7J1-FCC00-18F-D8[illegible]
Computer Monitor		Bwizibwera-HC IV-27547	DEL		CN/07CXPR-7287
Computer Monitor		Bwizibwera-HC IV-27561		HSTND-3761-Q	CNK6461ZPH
Computer Monitor		Mbarara-RRH-724	DELL	E220H	CN-OTYH11-FCC00-15M
Computer Monitor		Mbarara-RRH-761	DELL	E1920H	CN-ONKOPa-FCCOO-IBF-C8LB-A[illegible]
Computer Monitor		Mbarara-RRH-782	DELL	E1920H	CN-ONKOP9-FCOO-IBN-AULB-A[illegible]
Computer Monitor		Mbarara-RRH-784	DELL	E1920H	CN-ONKOP9-FCOO-IBN-AULB-A[illegible]
Computer Monitor		Mbarara-RRH-811	DELL	SUNON MAGLEV	CN-07CXPR-72872-727A66B-A0[illegible]
Computer Monitor		Mbarara-RRH-812	DELL	SUNON MAGLEV	CN-07CXPR-72872-727A66B-A0[illegible]
Computer Monitor		Mbarara-RRH-813	DELL	SUNON MAGLEV	CN-07CXPR-72872-727A66B-A0[illegible]
Computer Monitor		Mbarara-RRH-814	DELL	SUNON MAGLEV	CN-07CXPR-72872-727A66B-A0[illegible]
Computer Monitor		Mbarara-RRH-815	DELL	SUNON MAGLEV	CN-07CXPR-72872-727A66B-A0[illegible]
Computer Monitor		Mbarara-RRH-816	DELL	SUNON MAGLEV	CN-07CXPR-72872-727A66B-A0[illegible]
Computer Monitor		Mbarara-RRH-817	DELL	SUNON MAGLEV	CN-07CXPR-72872-727A66B-A0[illegible]

Equipment Name	Type	System ID	Model Name	Model No.	Serial No.
Computer Monitor		Mbarara-RRH-1434	HP		CNC442NXNW
Computer Monitor		Mbarara-RRH-1438	ACER	VI96HQL	MML YOEEOO7309001FA8501
Computer Monitor		Mbarara-RRH-31652	Sango CT Work place	DSC 1910-D	E151108559
Computer Monitor		Mbarara-RRH-31654			6CM431O745
Computer Monitor		Mbarara-RRH-31661		12041303	502801381
Computer Monitor		Mbarara-RRH-31662	DSC2417-D		229235
Computer Monitor		Mbarara-RRH-31663	DSC2417-D		229238
Computer Printer		Mbarara-RRH-1435	HP COLOR LASER		
Computer Printer		Mbarara-RRH-1436	HP LASERJET P2035	CE461A	VNB3L01006
Computer Printer		Mbarara-RRH-31653	Hp		OxE279-64180-6BS-0THS
Computer Printer		Mbarara-RRH-31656	Hp	T6B70A	VNC 3826563
Computer Printer		Mbarara-RRH-31659	DRY PIX 6000		16954835
Computer Printer		Mbarara-RRH-31668	SEOLA-1801-03		CNBLQ5QGT5
Computer Printer		Bwizibwera-HC IV-27562		M521DW	CNDKL-392J2
Computer Printer		Mbarara-RRH-733	HP	W1A52A	PHCD214041
Computer Printer		Mbarara-RRH-734	HP	W1A52A	PHCD214041
Computer Printer		Mbarara-RRH-735	HP	W1A52A	PHCD214041
Computer Printer		Mbarara-RRH-736	HP	W1A52A	PHCD214041
Computer Printer		Mbarara-RRH-737	HP	W1A52A	PHCD214041
Computer Printer		Mbarara-RRH-738	HP	W1A52A	PHCD214041
Computer Printer		Mbarara-RRH-739	HP	W1A52A	PHCD214041
Computer Printer		Mbarara-RRH-740	HP	W1A52A	PHCD214041
Computer Printer		Mbarara-RRH-741	HP	W1A52A	PHCD214041
Computer Printer		Mbarara-RRH-742	HP	W1A52A	PHCD214041
Computer		Mbarara-	HP	W1A52A	PHCD214041

Equipment Name	Type	System ID	Model Name	Model No.	Serial No.
Printer		RRH-743			
Computer Printer		Mbarara-RRH-744	HP	W1A52A	PHCD214041
Computer Printer		Mbarara-RRH-745	HP	W1A52A	PHCD214041
Computer Printer		Mbarara-RRH-746	HP	W1A52A	PHCD214041
Computer Printer		Mbarara-RRH-747	HP	W1A52A	PHCD214041
Computer Printer		Mbarara-RRH-748	HP	W1A52A	PHCD214041
Computer Printer		Mbarara-RRH-749	HP	W1A52A	PHCD214041
Computer Printer		Mbarara-RRH-750	HP	W1A52A	PHCD214041
Computer Printer		Mbarara-RRH-751	HP	W1A52A	PHCD214041
Computer Printer		Mbarara-RRH-904	HP	MFPM430	CNBRQ184L3
Computer Printer		Mbarara-RRH-905	HP	MFPM430	CNBRQ184L3
Computer Printer		Mbarara-RRH-906	HP	MFPM430	CNBRQ184L3
Computer Printer		Mbarara-RRH-907	HP	MFPM430	CNBRQ184L3
Computer Printer		Mbarara-RRH-908	HP	MFPM430	CNBRQ184L3
Computer Printer		Mbarara-RRH-909	HP	MFPM430	CNBRQ184L3
Computer Printer		Mbarara-RRH-910	HP	MFPM430	CNBRQ184L3
Computer Printer		Mbarara-RRH-911	HP	MFPM430	CNBRQ184L3
Computer Printer		Mbarara-RRH-912	HP	MFPM430	CNBRQ184L3
Computer Printer		Mbarara-RRH-913	HP	MFPM430	CNBRQ184L3
Computer Printer		Mbarara-RRH-914	HP	MFPM430	CNBRQ184L3
Computer Printer		Mbarara-RRH-915	HP	MFPM430	CNBRQ184L3
Computer Printer		Mbarara-RRH-969	HP	MFPM430	CNBRQ184KB
Computer Printer		Mbarara-RRH-970	HP	MFPM430	CNBRQ184KB
Computer Printer		Mbarara-RRH-971	HP	MFPM430	CNBRQ184KB
Computer Printer		Mbarara-RRH-972	HP	MFPM430	CNBRQ184KB
Computer Printer		Mbarara-RRH-973	HP	MFPM430	CNBRQ184KB
Computer Printer		Mbarara-RRH-974	HP	MFPM430	CNBRQ184KB

Equipment Name	Type	System ID	Model Name	Model No.	Serial No.
Computer Printer		Mbarara-RRH-975	HP	MFPM430	CNBRQ184KB
Computer Printer		Mbarara-RRH-976	HP	MFPM430	CNBRQ184KB
Computer Printer		Mbarara-RRH-977	HP	MFPM430	CNBRQ184HH
Computer Printer		Mbarara-RRH-978	HP	MFPM430	CNBRQ184HH
Computer Printer		Mbarara-RRH-979	HP	MFPM430	CNBRQ184HH
Computer Printer		Mbarara-RRH-980	HP	MFPM430	CNBRQ184HH
Computer Printer		Mbarara-RRH-981	HP	MFPM430	CNBRQ184HH
Computer Printer		Mbarara-RRH-1154	HP		CZC1447879
Computer Printer		Mbarara-RRH-1185	CANON	iR1024iF	(21)DRQ55579
Computer Printer		Mbarara-RRH-1186	CANON	iR1024iF	(21)DRQ55579
Computer Printer		Mbarara-RRH-1188	HP laserjet	600M601	CZC1447879
Computer Printer		Mbarara-RRH-1190	HP laserjet	P1102	VNF8YO4693
Computer Printer		Mbarara-RRH-1191	HP laserjet	P1102	VNF8YO4693
Computer Printer		Mbarara-RRH-1192	HP laserjet	P2035	CNCOPO4328
Computer Printer		Mbarara-RRH-1193	HP laserjet	P2035	CNCOPO4328
Computer Printer		Mbarara-RRH-1194	HP laserjet	P2035	CNCOPO4328
Computer Printer		Mbarara-RRH-1196	HP laserjet	Pro M102a	VNF3B86922
Computer Printer		Mbarara-RRH-1197	HP laserjet	Pro M102a	VNF3B86922
Computer Printer		Mbarara-RRH-1198	HP laserjet	Pro 400M401dn	VNH 4220424
Computer Printer		Mbarara-RRH-1200	CANON	Image runner2206	212FA44066
Computer Printer		Mbarara-RRH-1202	HP DESKJET	Deskjet3055A	CN33Q1CGBN
Computer Printer		Mbarara-RRH-1204	HP laserjet pro	Pro M404dn	VNF3D18325
Computer Printer		Mbarara-RRH-1205	HP laserjet pro	Pro M404dn	VNF3D18325
Computer Printer		Mbarara-RRH-1206	HP laserjet pro	Pro M404dn	VNF3D18325
Computer Printer		Mbarara-RRH-1207	HP laserjet pro	Pro M404dn	VNF3D18325
Computer Printer		Mbarara-RRH-1208	HP laserjet pro	Pro M404dn	VNF3D18325
Computer		Mbarara-	HP laserjet pro	Pro M404dn	VNF3D18325

Equipment Name	Type	System ID	Model Name	Model No.	Serial No.
Printer		RRH-1209			
Computer Printer		Mbarara-RRH-1210	HP laserjet pro	Pro M404dn	VNF3D18325
Computer Printer		Mbarara-RRH-1211	HP laserjet pro	Pro M404dn	VNF3D18325
Computer Printer		Mbarara-RRH-1212	HP laserjet pro	Pro M404dn	VNF3D18325
Computer Printer		Mbarara-RRH-1213	HP laserjet pro	Pro M404dn	VNF3D18325
Computer Printer		Mbarara-RRH-1214	HP laserjet pro	Pro M404dn	VNF3D18325
Computer Printer		Mbarara-RRH-1215	HP laserjet pro	Pro M404dn	VNF3D18325
Computer Printer		Mbarara-RRH-1216	HP laserjet pro	Pro M404dn	VNF3D18325
Computer Printer		Mbarara-RRH-1217	HP laserjet pro	Pro M404dn	VNF3D18325
Computer Printer		Mbarara-RRH-1218	HP laserjet pro	Pro M404dn	VNF3D18325
Computer Printer		Mbarara-RRH-1219	HP laserjet pro	Pro M404dn	VNF3D18325
Computer Printer		Mbarara-RRH-1220	HP laserjet pro	Pro M404dn	VNF3D18325
Computer Printer		Mbarara-RRH-1221	HP laserjet pro	Pro M404dn	VNF3D18325
Computer Printer		Mbarara-RRH-1223	HP laserjet		
Computer Printer		Mbarara-RRH-1224	HP laserjet	Pro M102A	VNF3B86922
Computer Printer		Mbarara-RRH-1226	HP laserjet	Pro M201dw	VNC3K25348
Computer Printer		Mbarara-RRH-1262		(21)6034441	(01)04548736044678
Computer Printer		Ruhoko-HC IV-27674			
Computer, Desktop		Mbarara-RRH-1078			CZC013B9MK
Computer, Desktop		Mbarara-RRH-1147	DELL	D12M	110711-11
Computer, Desktop		Mbarara-RRH-1148	DELL	D18M	150051-15
Computer, Desktop		Mbarara-RRH-1149	HP		CZC5123FPL
Computer, Desktop		Mbarara-RRH-1150	HP	X21	CRW6HQ2
Computer, Desktop		Mbarara-RRH-1152	HP	PRODesk	CXZC013B9MF
Computer, Desktop		Mbarara-RRH-1156	DELL	D19M	D19M003
Computer, Desktop		Mbarara-RRH-1157	DELL	DC8M	HBK7L3J
Computer, Desktop		Mbarara-RRH-1159	DELL	OPTiPLEX 7080	1WCX5C3

Equipment Name	Type	System ID	Model Name	Model No.	Serial No.
Computer, Desktop		Mbarara-RRH-1161	DELL	D19A	463L0S1
Computer, Desktop		Mbarara-RRH-1162	DELL	D19A	463L0S1
Computer, Desktop		Mbarara-RRH-1163	DELL	D19A	463L0S1
Computer, Desktop		Mbarara-RRH-1164	DELL	D19A	463L0S1
Computer, Desktop		Mbarara-RRH-1165	DELL	D19A	463L0S1
Computer, Desktop		Mbarara-RRH-1166	DELL	D19A	463L0S1
Computer, Desktop		Mbarara-RRH-1167	DELL	D19A	463L0S1
Computer, Desktop		Mbarara-RRH-1168	DELL	D19A	463L0S1
Computer, Desktop		Mbarara-RRH-1169	DELL	D19A	463L0S1
Computer, Desktop		Mbarara-RRH-1170	DELL	D19A	463L0S1
Computer, Desktop		Mbarara-RRH-1171	DELL	D19A	463L0S1
Computer, Desktop		Mbarara-RRH-1178	DELL	DESKTOP	GBVH2F2
Computer, Desktop		Mbarara-RRH-1180	DELL	DESKTOP 025	CZC013B9R6
Computer, Desktop		Mbarara-RRH-1182	DELL	LAPTOP	1PPVXN2
Computer, Desktop		Mbarara-RRH-1183	HP-SYSMEX	IPU	CZC0334Z5P
Computer, Laptop		Mbarara-RRH-1174	DELL	LAPTOP	7DFX6A00
Computer, Laptop		Mbarara-RRH-1175	DELL	LAPTOP	
CT Scan, 16/32 Slice	ME	Mbarara-RRH-31651		10165880	85529
CT Scan, 16/32 Slice	ME	Mbarara-RRH-31671		11101885	1918
Cupboard, Instrument	MF	Bugamba-HC IV-32409			
Cutlery Set	HSS	Kabuyanda-HC IV-27869			
Cylinder Manifold System, Oxygen	HSS	Nyamuyanja-HC IV-28030			
Cylinder Manifold System, Oxygen	HSS	Kabuyanda-HC IV-27960			
Deep Freezer, Blood (-20 to -30 degrees celsius)	HSS	Ishongororo-HC IV-27622			
Deep Freezer,	HSS	Bwizibwera-	Vest Frost		20181607856

Equipment Name	Type	System ID	Model Name	Model No.	Serial No.
Blood (-20 to -30 degrees celsius)		HC IV-27549			
Defibrillator Tester	HSS	Mbarara-RRH-1058			OES200024
Delivery Bed	MF	Kiruhura-HC IV-27837	ARELAX	1080	0012010000017
Delivery Bed	MF	Kiruhura-HC IV-27839	ARELAX	1080	
Delivery Bed	MF	Kyabugimbi-HC IV-29519		KL-2C	
Delivery Bed	MF	Kyabugimbi-HC IV-30100			
Delivery Bed	MF	Kyabugimbi-HC IV-30169			
Delivery Bed	MF	Kyabugimbi-HC IV-30364			
Delivery Bed	MF	Kyabugimbi-HC IV-30365			
Delivery Bed	MF	Kyabugimbi-HC IV-30367			
Delivery Bed	MF	Bushenyi-HC IV-29482			
Delivery Bed	MF	Ruhoko-HC IV-27708			
Delivery Bed	MF	Ruhoko-HC IV-27709			
Delivery Bed	MF	Kabuyanda-HC IV-27929			
Delivery Bed	MF	Kazo-HC IV-27746			
Delivery Bed	MF	Kazo-HC IV-27760		KL-2C	
Delivery Bed	MF	Kiruhura-HC IV-27827		KL-2C	
Delivery Bed	MF	Bugamba-HC IV-30422			
Delivery Bed	MF	Bugamba-HC IV-30423			
Delivery Bed	MF	Bugamba-HC IV-32408			
Delivery Bed	MF	Bugamba-HC IV-32420			
Delivery Bed	MF	Rugazi-HC IV-28552			
Delivery Bed	MF	Rugazi-HC IV-28930			
Delivery Bed	MF	Shuuku-HC IV-29481			
Delivery Bed (for persons with disability)	MF	Kiruhura-HC IV-27838		KL-2C	
Delivery Bed (for persons with	MF	Bwizibwera-HC IV-27539			

Equipment Name	Type	System ID	Model Name	Model No.	Serial No.
disability)					
Delivery Bed (for persons with disability)	MF	Bwizibwera-HC IV-27557			
Delivery Bed (for persons with disability)	MF	Ishongororo-HC IV-27638			
Delivery Bed (for persons with disability)	MF	Ruhoko-HC IV-27689			
Delivery Bed (for persons with disability)	MF	Kazo-HC IV-27759		KL-2C	
Dental Chair		Ruhoko-HC IV-27718	UMS		
Dental Chair		Bugamba-HC IV-32410			
Dental X-ray Machine	ME	Mbarara-RRH-47453	GE	5189248	80930HL1
Differential Counter, Manual	ME	Kazo-HC IV-27742		DBC-8E	103269
Differential Counter, Manual	ME	Kazo-HC IV-27743		DBC-8E	103292
Drip Stand	MF	Kyabugimbi-HC IV-30353			
Drip Stand	MF	Kyabugimbi-HC IV-30354			
Drip Stand	MF	Kyabugimbi-HC IV-30366			
Drip Stand	MF	Kyabugimbi-HC IV-30408			
Drip Stand	MF	Kyabugimbi-HC IV-30409			
Drip Stand	MF	Bushenyi-HC IV-29002			
Drip Stand	MF	Bushenyi-HC IV-29003			
Drip Stand	MF	Bushenyi-HC IV-29015			
Drip Stand	MF	Nyamuyanja-HC IV-27971			
Drip Stand	MF	Nyamuyanja-HC IV-28028			
Drip Stand	MF	Kabuyanda-HC IV-27863			
Drip Stand	MF	Kabuyanda-HC IV-27882			
Drip Stand	MF	Kabuyanda-HC IV-27931			
Drip Stand	MF	Kabuyanda-HC IV-27963			
Drip Stand	MF	Kinoni-HC IV-28048			

Equipment Name	Type	System ID	Model Name	Model No.	Serial No.
Drip Stand	MF	Kinoni-HC IV-28049			
Drip Stand	MF	Kinoni-HC IV-28050			
Drip Stand	MF	Kinoni-HC IV-28051			
Drip Stand	MF	Kinoni-HC IV-28052			
Drip Stand	MF	Kinoni-HC IV-28053			
Drip Stand	MF	Kinoni-HC IV-28054			
Drip Stand	MF	Kinoni-HC IV-28055			
Drip Stand	MF	Kinoni-HC IV-28056			
Drip Stand	MF	Kinoni-HC IV-28057			
Drip Stand	MF	Kinoni-HC IV-28058			
Drip Stand	MF	Kinoni-HC IV-28059			
Drip Stand	MF	Kinoni-HC IV-28060			
Drip Stand	MF	Kinoni-HC IV-28061			
Drip Stand	MF	Kinoni-HC IV-28062			
Drip Stand	MF	Kinoni-HC IV-28063			
Drip Stand	MF	Kinoni-HC IV-28064			
Drip Stand	MF	Kinoni-HC IV-28065			
Drip Stand	MF	Bugamba-HC IV-30425			
Drip Stand	MF	Bugamba-HC IV-30441			
Drip Stand	MF	Bugamba-HC IV-30442			
Drip Stand	MF	Bugamba-HC IV-30452			
Drip Stand	MF	Bugamba-HC IV-32321			
Drip Stand	MF	Bugamba-HC IV-32393			
Drip Stand	MF	Bugamba-HC IV-32411			
Drip Stand	MF	Bugamba-HC IV-32427			
Drip Stand	MF	Rugazi-HC IV-28892			
Drip Stand	MF	Rugazi-HC			

Equipment Name	Type	System ID	Model Name	Model No.	Serial No.
		IV-28919			
Drip Stand	MF	Rugazi-HC IV-28931			
Drip Stand	MF	Rugazi-HC IV-28934			
Drip Stand	MF	Kabwohe-HC IV-29823			
Drip Stand	MF	Kabwohe-HC IV-29845			
Drip Stand	MF	Shuuku-HC IV-29513			
Drip Stand	MF	Shuuku-HC IV-29536			
Drip Stand	MF	Mbarara-RRH-1263			2019z90785
Drug Cabinet		Mbarara-RRH-2748			
Drug Cabinet		Bugamba-HC IV-32324			
Drug Cabinet		Bugamba-HC IV-32325			
Drug Cabinet		Shuuku-HC IV-29516			
Drug Cabinet		Kyabugimbi-HC IV-30352			
Drug Cabinet		Kyabugimbi-HC IV-30399			
Drug Cabinet		Bushenyi-HC IV-29000			
Drug Cabinet		Kabuyanda-HC IV-27879			
ECG, 12 Channel		Mbarara-RRH-1266		SE-1200Express	460016-M19600700002
ECG, 12 Lead	ME	Mbarara-RRH-1056	EKG1212T		21021500005
Electrical Safety Analyzer	HSS	Bushenyi-HC IV-29476	Humalyte plus		2105245
Electrical suction machine	ME	Kyabugimbi-HC IV-30098	YX920S		
Electrical suction machine	ME	Kyabugimbi-HC IV-30345			133070
Electrical suction machine	ME	Kyabugimbi-HC IV-30346			1655PM-01/08
Electrical suction machine	ME	Bushenyi-HC IV-28944	FA-23D		00029
Electrical suction machine	ME	Bushenyi-HC IV-29004	7A-23B		00001
Electrical suction machine	ME	Ishongororo-HC IV-27618			844PM-11/05
Electrical suction machine	ME	Ishongororo-HC IV-27624		YX940D	
Electrical suction machine	ME	Ruhoko-HC IV-27699		220-1	

Equipment Name	Type	System ID	Model Name	Model No.	Serial No.
Electrical suction machine	ME	Kabuyanda-HC IV-27856		YX980D	014212201
Electrical suction machine	ME	Kazo-HC IV-27747		SM-180	00075
Electrical suction machine	ME	Kazo-HC IV-27754		CA-M1	2833
Electrical suction machine	ME	Kiruhura-HC IV-27829		F-3000	10003
Electrical suction machine	ME	Mbarara-RRH-1280	Eurovac Elite		TGE20845
Electrical suction machine	ME	Mbarara-RRH-1497	ASKIR	ASKIR C20 BASIC 2	9333
Electrical suction machine	ME	Mbarara-RRH-2508	ASKIRC30		11804
Electrical suction machine	ME	Mbarara-RRH-34111	SAM 35	01050102	0223-0179
Electrical suction machine	ME	Mbarara-RRH-34112	SAM35	01050102	0223-0178
Electrical suction machine	ME	Mbarara-RRH-34113	SAM35	01050102	0223-0180
Electrical suction machine	ME	Bugamba-HC IV-30454	YX940D		IF3.061
Electrical suction machine	ME	Bugamba-HC IV-30455	YX940D		IF3.061
Electrical suction machine	ME	Bugamba-HC IV-30456	YX940D		IF3.061
Electrical suction machine	ME	Rugazi-HC IV-28570	F-90		1500009
Electrical suction machine	ME	Rugazi-HC IV-28904	5320231		9760-47006
Electrical suction machine	ME	Rugazi-HC IV-28905	RE210300		7749
Electrical suction machine	ME	Rugazi-HC IV-28906	RE210300		7685
Electrical suction machine	ME	Kabwohe-HC IV-29807		V7	9760-47-021
Electrical suction machine	ME	Shuuku-HC IV-29504	F-30000		100033
Electrical suction machine	ME	Shuuku-HC IV-29515	F-170		090461
Electrical suction machine	ME	Bwizibwera-HC IV-27520	SM-180		00100
Electrical suction machine	ME	Bwizibwera-HC IV-27523	CA-MI		2812
Electrical suction machine	ME	Mbarara-RRH-381		410250	11801
Electrical suction machine	ME	Mbarara-RRH-382		310350107	
Electrical suction machine	ME	Mbarara-RRH-424			7GE20842
Electrical suction machine	ME	Mbarara-RRH-522	ASKIRC30	410250	12019
Electrical	ME	Mbarara-	ASKIRC30	410250	12011

Equipment Name	Type	System ID	Model Name	Model No.	Serial No.
suction machine		RRH-525			
Electrical suction machine	ME	Mbarara-RRH-526		SM-180	00054
Electrical suction machine	ME	Mbarara-RRH-527		SM-180	00054
Electrical suction machine	ME	Mbarara-RRH-584		RE410250	12012
Electro Surgical Unit	ME	Kabuyanda-HC IV-27854		EK-410	3076097308
Examination Couch	MF	Kyabugimbi-HC IV-29540			
Examination Couch	MF	Kyabugimbi-HC IV-30347			
Examination Couch	MF	Kyabugimbi-HC IV-30368			
Examination Couch	MF	Ishongororo-HC IV-27603			
Examination Couch	MF	Ishongororo-HC IV-27607			
Examination Couch	MF	Ishongororo-HC IV-27621			
Examination Couch	MF	Ishongororo-HC IV-27654			
Examination Couch	MF	Ishongororo-HC IV-27655			
Examination Couch	MF	Ruhoko-HC IV-27695			
Examination Couch	MF	Ruhoko-HC IV-27710			
Examination Couch	MF	Ruhoko-HC IV-27720			
Examination Couch	MF	Nyamuyanja-HC IV-27969			
Examination Couch	MF	Nyamuyanja-HC IV-27973			
Examination Couch	MF	Kabuyanda-HC IV-27927			
Examination Couch	MF	Kabuyanda-HC IV-28012			
Examination Couch	MF	Kazo-HC IV-27765			
Examination Couch	MF	Bwizibwera-HC IV-27545			
Examination Couch	MF	Mbarara-RRH-512			
Examination Couch	MF	Bugamba-HC IV-32405			
Examination Couch	MF	Bugamba-HC IV-32406			
Examination Couch	MF	Bugamba-HC IV-32413			
Examination Couch	MF	Bugamba-HC IV-32422			

Equipment Name	Type	System ID	Model Name	Model No.	Serial No.
Examination Couch	MF	Kabwohe-HC IV-29818			
Examination Couch	MF	Kabwohe-HC IV-29820			
Examination Couch	MF	Kabwohe-HC IV-30074			
Examination Couch	MF	Shuuku-HC IV-29747			
Phlebotomy Couch	MF	Rugazi-HC IV-28936			
Phlebotomy Couch	MF	Rugazi-HC IV-28937			
Examination Light	ME	Bugamba-HC IV-30424	KS-Q6		
Examination Light	ME	Rugazi-HC IV-28572	YDE10		
Examination Light	ME	Shuuku-HC IV-29503	YDE10		DZ20211224
Examination Light	ME	Ishongororo-HC IV-27597		YDOIA (LED)	
Examination Light	ME	Ishongororo-HC IV-27631			
Examination Light	ME	Ruhoko-HC IV-27693			
Examination Light	ME	Kazo-HC IV-27748		EK-Q3	
Examination Light	ME	Kazo-HC IV-27762			
Examination Light	ME	Bwizibwera-HC IV-27541			
Feeding Pump	ME	Mbarara-RRH-34107	Amika ZA	Z044191	25504141
Feeding Pump	ME	Mbarara-RRH-34108	Amika ZA	Z044191	25504123
Feeding Pump	ME	Mbarara-RRH-34109	Amika ZA	Z044191	25504135
Fetoscope, Doppler		Nyamuyanja-HC IV-28040	SONOTRAX BASIC		550042-M18509280086
Filing Cabinet	HSS	Shuuku-HC IV-29498			
Fire Extinguisher	HSS	Mbarara-RRH-1234	FIRE BLANKET		
Fire Extinguisher	HSS	Mbarara-RRH-1235			
Fire Extinguisher	HSS	Mbarara-RRH-1236			
Fire Extinguisher	HSS	Mbarara-RRH-1237			
Fire Extinguisher	HSS	Mbarara-RRH-1238			
Fire Extinguisher	HSS	Mbarara-RRH-1239			
Fire Extinguisher	HSS	Mbarara-			

Equipment Name	Type	System ID	Model Name	Model No.	Serial No.
		RRH-1240			
Fire Extinguisher	HSS	Mbarara-RRH-1241			
Fire Extinguisher	HSS	Mbarara-RRH-1242			
Fire Extinguisher	HSS	Mbarara-RRH-1243			
First Aid Kit	MES	Mbarara-RRH-1244			
First Aid Kit	MES	Mbarara-RRH-1245			
Gas Cylinders		Rugazi-HC IV-28911			
Generator Three Phase, 15 - 80KW		Kiruhura-HC IV-27847			101001923
Generator Three Phase, 15 - 80KW		Kyabugimbi-HC IV-30372			
Generator Three Phase, 15 - 80KW		Ishongororo-HC IV-27598	YAMAHA	EF6600E	
Generator Three Phase, 15 - 80KW		Kabuyanda-HC IV-27979	DUETZ		136885/33
GeneXpert machine	ME	Ruhoko-HC IV-27673			805885
GeneXpert machine	ME	Kabuyanda-HC IV-28010			
GeneXpert machine	ME	Kazo-HC IV-27731			
GeneXpert machine	ME	Kiruhura-HC IV-27817	CEPHEID		809775
GeneXpert machine	ME	Mbarara-RRH-1111	Cepheid 16M	GX-XVIR2	84431
GeneXpert machine	ME	Mbarara-RRH-1256	CEPHEID	GX-IVR2	809775
GeneXpert machine	ME	Mbarara-RRH-1257	CEPHEID	GX-IVR2	803501
GeneXpert machine	ME	Bwizibwera-HC IV-27560			816686
Glassware Set, Laboratory, Basic	MES	Kabuyanda-HC IV-27871			
Glucometer	ME	Bushenyi-HC IV-29480			
Glucometer	ME	Kabuyanda-HC IV-27905	Freestyle optimum	0086	XEMT3656
Glucometer	ME	Kabuyanda-HC IV-27907	ONE CALL PLUS		103L11EE3AD
Glucometer	ME	Kabuyanda-HC IV-27998	One touch		L2289RB00185
Glucometer	ME	Kabuyanda-	One touch		SANBN3MM

Equipment Name	Type	System ID	Model Name	Model No.	Serial No.
		HC IV-28001			
Glucometer	ME	Kabuyanda-HC IV-28002	ONE CALL PLUS		103A309E90A
Glucometer	ME	Bugamba-HC IV-32404	ONE TOUCH		SAGVTND9
Glucometer	ME	Kabwohe-HC IV-29918			
Glucometer	ME	Kabwohe-HC IV-29919			
Glucometer	ME	Kabwohe-HC IV-29920			
Glucometer	ME	Kabwohe-HC IV-29921			
Glucometer	ME	Mbarara-RRH-1189	Dr's	2017.11	FC17KB00102
Glucometer	ME	Mbarara-RRH-1195	One touch		
Glucometer	ME	Mbarara-RRH-1199	One touch		
Glucometer	ME	Mbarara-RRH-1201	One touch		
Glucometer	ME	Mbarara-RRH-1203	One touch		
Glucometer	ME	Mbarara-RRH-1222	One touch		
Glucometer	ME	Mbarara-RRH-1225	Dr's		FC17KB00095
Glucometer	ME	Mbarara-RRH-1227	Wellion		59106000461
HB Meter, Heamocue		Kyabugimbi-HC IV-29551	Diaspect tm		20TB5073
HB Meter, Heamocue		Bushenyi-HC IV-29479	HB301		1737821005
HB Meter, Heamocue		Bugamba-HC IV-32401		3040-0010-0218	3040-22-0835
HB Meter, Heamocue		Bugamba-HC IV-32402		3040-0010-0218	3040-22-0802
HB Meter, Heamocue		Kabwohe-HC IV-29925	AICEZ 2.0		ETS20920170
HB Meter, Heamocue		Kabwohe-HC IV-29947		3040-0010-0218	3040-22-0820
HB Meter, Heamocue		Kabwohe-HC IV-29949		3040-0010-0218	3040-22-0858
Hb Meter, Sahli		Kabuyanda-HC IV-27992		HB301	1947821768
Height Meter, Adult	ME	Kyabugimbi-HC IV-29542			
Height Meter, Adult	ME	Kyabugimbi-HC IV-29543			
Height Meter, Adult	ME	Nyamuyanja-HC IV-27959			
Height Meter, Adult	ME	Nyamuyanja-HC IV-27964			

Equipment Name	Type	System ID	Model Name	Model No.	Serial No.
Height Meter, Adult	ME	Kabwohe-HC IV-29937			
Height Meter, Adult	ME	Kabwohe-HC IV-30079			
Height Meter, Adult	ME	Kabwohe-HC IV-30080			
Hematology Analyser, 3-part	ME	Kyabugimbi-HC IV-29549	Humacount 30TS		8250028
Hematology Analyser, 3-part	ME	Bushenyi-HC IV-29478	MEK-6510K		06009
Hematology Analyser, 3-part	ME	Ruhoko-HC IV-27675	HUMA COUNT30 TS		820388
Hematology Analyser, 3-part	ME	Kabuyanda-HC IV-28015		MEK-65510K	0817
Hematology Analyser, 3-part	ME	Kazo-HC IV-27729	HUMA Count 60		921065
Hematology Analyser, 3-part	ME	Kazo-HC IV-27737	HUMALYTE PLUS		1407115
Hematology Analyser, 3-part	ME	Kazo-HC IV-27738	HUMASTAR 80		21146
Hematology Analyser, 3-part	ME	Kiruhura-HC IV-27819	HUMA Count 30		825002762
High Flow Oxygen Therapy Machine	ME	Mbarara-RRH-2687		MP04201	QMHF20205285
High Flow Oxygen Therapy Machine	ME	Mbarara-RRH-2688		MP04201	QMHF20205196
High Flow Oxygen Therapy Machine	ME	Mbarara-RRH-2693		MP04201	QMHF20205284
Hot Air Oven	ME	Rugazi-HC IV-28558			
Hot Air Oven	ME	Rugazi-HC IV-28938	202-0A		20210107140103
Hot Air Oven	ME	Kyabugimbi-HC IV-30359			
Hot Air Oven	ME	Kabuyanda-HC IV-27987		HH-B11-500BY-II	211376
Hot Air Oven	ME	Kabuyanda-HC III-27895		GZX-CF10-1-4-5	211482
ICU Bed with weighing facility	MF	Mbarara-RRH-379		AG-DY008	RS/CP20190715018
ICU Bed with weighing facility	MF	Mbarara-RRH-380		AG-DY009	RS/CP20190N039001
Incubator, Culture	ME	Bwizibwera-HC IV-27554			
Incubator, Culture	ME	Bushenyi-HC IV-29472	BJPX-H54BK(B)		BJPXH5452102025
Incubator, Culture	ME	Ruhoko-HC IV-27672		IN55	
Incubator, Culture	ME	Mbarara-RRH-987	BINDER HOT AIR OVEN	ED 240	13-11-64

Equipment Name	Type	System ID	Model Name	Model No.	Serial No.
Incubator, Culture	ME	Mbarara-RRH-995	FISHER BRAND		42496142
Incubator, Culture, Aerobic	ME	Mbarara-RRH-986	MEMMERT	ISE300	C 3980090
Incubator, Thermal Range up to 1800C	ME	Mbarara-RRH-731	BIOBASE	BJPX-H54BK(D)	BJPXH5452102028
Infant Warmer	ME	Bwizibwera-HC IV-27538		BN-100A	1160906023
Infant Warmer	ME	Mbarara-RRH-391	BABYTHERM8004		
Infant Warmer	ME	Mbarara-RRH-406		1WS3300	HCCM50501
Infant Warmer	ME	Mbarara-RRH-419		NOC100	2462
Infant Warmer	ME	Mbarara-RRH-491		IR-200	22AIA202002
Infant Warmer	ME	Mbarara-RRH-498		EP300	70PTO5201312
Infant Warmer	ME	Mbarara-RRH-545		IR-200	22AIAZO2002
Infant Warmer	ME	Mbarara-RRH-547	IR-200		22AIAZ02002
Infant Warmer	ME	Mbarara-RRH-590		EP300	70PTO5201316
Infant Warmer	ME	Mbarara-RRH-591		EP300	70PTO5201316
Infant Warmer	ME	Mbarara-RRH-592		EP300	70PTO5201316
Infant Warmer	ME	Mbarara-RRH-600		BN-100A	1191119062
Infant Warmer	ME	Mbarara-RRH-1278	Warmer		W15105018
Infant Warmer	ME	Mbarara-RRH-1284	Air-shield system 7830		VV10019
Infant Warmer	ME	Mbarara-RRH-1286		BRW-3000B	211082452
Infant Warmer	ME	Bushenyi-HC IV-28943	IR-200		A22BAKHO2001
Infant Warmer	ME	Ruhoko-HC IV-27687	BRW-3000B		211082442
Infant Warmer	ME	Nyamuyanja-HC IV-28019		IR-200	A22BB0035001
Infant Warmer	ME	Nyamuyanja-HC IV-28023			17031
Infant Warmer	ME	Kabuyanda-HC IV-27853	OHMEDA MEDICAL	3300	HCCN50500
Infant Warmer	ME	Kabuyanda-HC IV-27922		HKN90	21130303072
Infant Warmer	ME	Kabuyanda-HC IV-27958		BLR-2100	BDZ00119
Infant Warmer	ME	Rugazi-HC IV-28553	BN-100A		1160906010

Equipment Name	Type	System ID	Model Name	Model No.	Serial No.
Infusion Pump	ME	Mbarara-RRH-34116	Agilia VP MC ZA	Z019691	25415161
Infusion Pump	ME	Mbarara-RRH-34117	Agilia VP MC ZA	Z019691	25415175
Infusion Pump	ME	Mbarara-RRH-34118	Agilia SP MC ZA	Z018691	25446476
Infusion Pump	ME	Mbarara-RRH-1311	Aitecs	DF-12	
Infusion Pump	ME	Mbarara-RRH-1432	LIFECARE		12930806
Infusion Pump	ME	Mbarara-RRH-369		Z018631	24696738
Infusion Pump	ME	Mbarara-RRH-489		P600	XD20200925D064
Infusion Pump	ME	Mbarara-RRH-504		BENEINFUSION VP3	SK10105977
Infusion Pump	ME	Mbarara-RRH-586		P600	XD20200925D064
Infusion Pump	ME	Mbarara-RRH-587		P600	XD20200925D064
Infusion Pump	ME	Mbarara-RRH-588		P600	XD20200925D064
Infusion Pump	ME	Mbarara-RRH-606	BONE FUSSION	VP3	SK01204700
Infusion Pump	ME	Mbarara-RRH-608		P600	XD20200925B037
Infusion Pump	ME	Mbarara-RRH-615	BIO LIGHT P600		XD20200904B142
Infusion Pump	ME	Mbarara-RRH-618	BIO LIGHT P600		XD20200904B075
Infusion Pump	ME	Mbarara-RRH-620	BIO LIGHT P600		XD20200904B037
Infusion Pump	ME	Mbarara-RRH-622	BIO LIGHT P600		XD20200904B141
Infusion Pump	ME	Mbarara-RRH-623		P600	XD20200925B013
Infusion Pump	ME	Mbarara-RRH-624	BIO LIGHT P600		XD20200904B005
Infusion Pump	ME	Mbarara-RRH-625	BIO LIGHT P600		XD20200904B013
Infusion Pump	ME	Mbarara-RRH-718	BIO LIGHT P600		XD20200904B064
Infusion Pump	ME	Mbarara-RRH-719	BIO LIGHT P600		XD20200904B064
Infusion Pump	ME	Mbarara-RRH-720	BIO LIGHT P600		XD20200904B141
Infusion Pump	ME	Mbarara-RRH-721	BIO LIGHT P600		XD20200904B055

Equipment Name	Type	System ID	Model Name	Model No.	Serial No.
Infusion Pump	ME	Mbarara-RRH-730	Benefusion VP3		SK01204700
Infusion Pump	ME	Mbarara-RRH-732	Benefusion VP3		SK01204702
Infusion Pump	ME	Mbarara-RRH-752	Benefusion VP3		SK10105983
Infusion Pump	ME	Mbarara-RRH-754	Benefusion VP3		SK10105979
Infusion Pump	ME	Mbarara-RRH-755	Benefusion VP3		SK10105977
Infusion Pump	ME	Mbarara-RRH-757	Benefusion VP3		SK1204699
Infusion Pump	ME	Mbarara-RRH-759	Benefusion VP3		SK01204703
Infusion Pump	ME	Mbarara-RRH-760	Bene Fusion VP3		SK10105985
Infusion Pump	ME	Mbarara-RRH-762	Bene Fusion VP3		SK01204697
Infusion Pump	ME	Mbarara-RRH-764	Bene Fusion VP3		SK10105981
Infusion Pump	ME	Mbarara-RRH-765	Bene Fusion VP3		SK01204700
Infusion Pump	ME	Kabuyanda-HC IV-27865		8715440	200/230/240
Instrument set, BTL	MIS	Kabuyanda-HC IV-27873			
Instrument set, hernia, adult	MIS	Kabuyanda-HC IV-27872			
Instrument Trolley	MF	Kyabugimbi-HC IV-29523			
Instrument Trolley	MF	Kyabugimbi-HC IV-30091			
Instrument Trolley	MF	Kyabugimbi-HC IV-30122			
Instrument Trolley	MF	Kyabugimbi-HC IV-30341			
Instrument Trolley	MF	Kyabugimbi-HC IV-30342			
Instrument Trolley	MF	Kyabugimbi-HC IV-30343			
Instrument Trolley	MF	Kyabugimbi-HC IV-30344			
Instrument Trolley	MF	Kyabugimbi-HC IV-30369			
Instrument Trolley	MF	Kyabugimbi-HC IV-30396			
Instrument Trolley	MF	Bushenyi-HC IV-28999			
Instrument Trolley	MF	Bushenyi-HC IV-29014			
Instrument Trolley	MF	Nyamuyanja-HC IV-27991			
Instrument	MF	Nyamuyanja-			

Equipment Name	Type	System ID	Model Name	Model No.	Serial No.
Trolley		HC IV-28022			
Instrument Trolley	MF	Nyamuyanja-HC IV-28043			
Instrument Trolley	MF	Kabuyanda-HC IV-27857			
Instrument Trolley	MF	Kabuyanda-HC IV-27858			
Instrument Trolley	MF	Kabuyanda-HC IV-27932			
Instrument Trolley	MF	Mbarara-RRH-2686			
Instrument Trolley	MF	Mbarara-RRH-2695			
Instrument Trolley	MF	Kinoni-HC IV-28067			
Instrument Trolley	MF	Bugamba-HC IV-30429			
Instrument Trolley	MF	Bugamba-HC IV-32322			
Instrument Trolley	MF	Bugamba-HC IV-32323			
Instrument Trolley	MF	Bugamba-HC IV-32407			
Instrument Trolley	MF	Rugazi-HC IV-28564			
Instrument Trolley	MF	Rugazi-HC IV-28568			
Instrument Trolley	MF	Rugazi-HC IV-28569			
Instrument Trolley	MF	Rugazi-HC IV-28893			
Instrument Trolley	MF	Rugazi-HC IV-28897			
Instrument Trolley	MF	Rugazi-HC IV-28907			
Instrument Trolley	MF	Kabwohe-HC IV-29821			
Instrument Trolley	MF	Kabwohe-HC IV-29827			
Instrument Trolley	MF	Shuuku-HC IV-29487			
Instrument Trolley	MF	Shuuku-HC IV-29493			
Instrument Trolley	MF	Shuuku-HC IV-29526			
Instrument Trolley	MF	Shuuku-HC IV-30532			
Instrument Trolley	MF	Mbarara-RRH-403			
Instrument Trolley	MF	Mbarara-RRH-411			
Instrument Trolley	MF	Mbarara-RRH-416			

Equipment Name	Type	System ID	Model Name	Model No.	Serial No.
Instrument Trolley	MF	Mbarara-RRH-427			
Instrument Trolley	MF	Mbarara-RRH-459			
Instrument Trolley	MF	Mbarara-RRH-462			
Instrument Trolley	MF	Mbarara-RRH-477			
Instrument Trolley	MF	Mbarara-RRH-480			
Procedure Trolley	MF	Kabuyanda-HC IV-27974			
Procedure Trolley	MF	Mbarara-RRH-2745			
Inverter-Charge Backup System		Mbarara-RRH-1427		LV2505	
Inverter-Charge Backup System		Ruhoko-HC IV-27671			
Inverter-Charger Power backup system, Battery Bank	HSS	Bwizibwera-HC IV-27525	2900VASU-KAM		S/N:00206A10262702110127
Refrigerator, General Purpose	HSS	Kiruhura-HC IV-27843		P700	1492461
Refrigerator, General Purpose	HSS	Kiruhura-HC IV-27846		VC65FB	1689-005
Refrigerator, General Purpose	HSS	Kiruhura-HC IV-27848		VLS054	20157800615
Refrigerator, General Purpose	HSS	Kiruhura-HC IV-27849		HTCD-160	BEOH40E1T00QJM8N0015
Refrigerator, General Purpose	HSS	Bwizibwera-HC IV-27552			S/N 1B0302Z0036JBC5LL52004
Refrigerator, General Purpose	HSS	Bwizibwera-HC IV-27553	1783		
Refrigerator, General Purpose	HSS	Bwizibwera-HC IV-27555		TCW2000	0461943
Refrigerator, General Purpose	HSS	Bwizibwera-HC IV-27565	Thermo scientific		72801003
Refrigerator, General Purpose	HSS	Bwizibwera-HC IV-27567		RF/175	161103219097
Refrigerator, General Purpose	HSS	Ishongororo-HC IV-27570	Vest Frost		
Refrigerator, General Purpose	HSS	Ishongororo-HC IV-27615		L-476	
Refrigerator, General Purpose	HSS	Ishongororo-HC IV-27617		GL-E292LRVL	611NRJT025252
Refrigerator, General Purpose	HSS	Ruhoko-HC IV-27669		DCR165/C	70630103
Refrigerator, General Purpose	HSS	Ruhoko-HC IV-27680		MR-PR-400	MR-PR-400G62W1308V00011
Refrigerator, General Purpose	HSS	Ruhoko-HC IV-27682	GML	GR-M282	ME26221712
Refrigerator,	HSS	Ruhoko-HC	GLE292RLVL		JZ4WUDRR

Equipment Name	Type	System ID	Model Name	Model No.	Serial No.
General Purpose		IV-27683			
Refrigerator, General Purpose	HSS	Ruhoko-HC IV-27721		TCW3005DD	5162530
Refrigerator, General Purpose	HSS	Ruhoko-HC IV-27722		TCW2000SDD	
Refrigerator, General Purpose	HSS	Kabuyanda-HC IV-27968		HTCD-150	DE17000KAG0025
Refrigerator, General Purpose	HSS	Kabuyanda-HC IV-28006		HXC-158	BEO6L9E1T00B2CCS0003
Refrigerator, General Purpose	HSS	Kabuyanda-HC IV-28016		SW311M	74900988
Refrigerator, General Purpose	HSS	Kabuyanda-HC IV-28018		VIIOGE	84200008
Refrigerator, General Purpose	HSS	Kazo-HC IV-27726	32KHS105754		1B0092Z0272JBCPVFE70545
Refrigerator, General Purpose	HSS	Kazo-HC IV-27727	Thermo scientific		5042425444443265
Refrigerator, General Purpose	HSS	Kazo-HC IV-27735			2U052
Refrigerator, General Purpose	HSS	Kazo-HC IV-27736		ML380CSG	7462589
Refrigerator, General Purpose	HSS	Kazo-HC IV-27764		HTC-240	BE0G37EATOOQJM8A0018
Refrigerator, General Purpose	HSS	Kazo-HC IV-27766		TCW3000SDD	5162547
Refrigerator, General Purpose	HSS	Kazo-HC IV-27767	ELECTROLUX		
Refrigerator, General Purpose	HSS	Kiruhura-HC IV-27809		RCW42EG	1030667
Refrigerator, General Purpose	HSS	Kiruhura-HC IV-27811	Thermo scientific		70800875
Refrigerator, General Purpose	HSS	Rugazi-HC IV-28925	FKS411		20015
Refrigerator, Laboratory, Reagents	HSS	Ruhoko-HC IV-27679		ML380CSG	7462569
Refrigerator, Laboratory, Reagents	HSS	Mbarara-RRH-1249	BPR-5V310	BIO BASE	YC031025212971
Refrigerator, Laboratory, Reagents	HSS	Mbarara-RRH-1250	BIO BASE	BPR-5V310	YC031025212970
Laboratory Stool	MF	Bushenyi-HC IV-29475			
Laptop/ Computer with Accessories		Mbarara-RRH-31666		24011513652	212206
Laptop/ Computer with Accessories		Mbarara-RRH-727	DELL		5C26293
Laptop/ Computer with Accessories		Mbarara-RRH-756	VOSTRO	3910	DX936Q3

Equipment Name	Type	System ID	Model Name	Model No.	Serial No.
Laptop/ Computer with Accessories		Mbarara-RRH-763	DELL	D29N	8ZRJKG3
Laptop/ Computer with Accessories		Mbarara-RRH-807	DELL	D29N	615JKG3
Laptop/ Computer with Accessories		Mbarara-RRH-839	DELL	OPTIPLEX7040	GVBH2F2
Laptop/ Computer with Accessories		Mbarara-RRH-840	DELL	OPTIPLEX7040	GVBH2F2
Laptop/ Computer with Accessories		Mbarara-RRH-841	DELL	OPTIPLEX7040	GVBH2F2
Laptop/ Computer with Accessories		Mbarara-RRH-842	DELL	OPTIPLEX7040	GVBH2F2
Laptop/ Computer with Accessories		Mbarara-RRH-843	DELL	OPTIPLEX7040	GVBH2F2
Laptop/ Computer with Accessories		Mbarara-RRH-844	DELL	OPTIPLEX7040	GVBH2F2
Laptop/ Computer with Accessories		Mbarara-RRH-845	DELL	OPTIPLEX7040	GVBH2F2
Laptop/ Computer with Accessories		Mbarara-RRH-846	DELL	OPTIPLEX7040	GVBH2F2
Laptop/ Computer with Accessories		Mbarara-RRH-847	DELL	OPTIPLEX7040	GVBH2F2
Laptop/ Computer with Accessories		Mbarara-RRH-848	DELL	OPTIPLEX7040	GVBH2F2
Laptop/ Computer with Accessories		Mbarara-RRH-849	DELL	OPTIPLEX7040	GVBH2F2
Laptop/ Computer with Accessories		Mbarara-RRH-850	DELL	OPTIPLEX7040	GVBH2F2
Laptop/ Computer with Accessories		Mbarara-RRH-851	DELL	OPTIPLEX7040	GVBH2F2
Laptop/ Computer with Accessories		Mbarara-RRH-852	DELL	OPTIPLEX7040	GVBH2F2
Laptop/ Computer with Accessories		Mbarara-RRH-853	DELL	OPTIPLEX7040	GVBH2F2
Laptop/ Computer with Accessories		Mbarara-RRH-854	DELL	OPTIPLEX7040	GVBH2F2
Laptop/		Mbarara-	DELL	OPTIPLEX7040	GVBH2F2

Equipment Name	Type	System ID	Model Name	Model No.	Serial No.
Computer with Accessories		RRH-855			
Laptop/ Computer with Accessories		Mbarara-RRH-856	DELL	OPTIPLEX7040	GVBH2F2
Laptop/ Computer with Accessories		Mbarara-RRH-857	DELL	OPTIPLEX7040	GVBH2F2
Laptop/ Computer with Accessories		Mbarara-RRH-858	DELL	OPTIPLEX7040	GVBH2F2
Laptop/ Computer with Accessories		Mbarara-RRH-859	DELL	OPTIPLEX7040	GVBH2F2
Laptop/ Computer with Accessories		Mbarara-RRH-860	DELL	OPTIPLEX7040	GVBH2F2
Laptop/ Computer with Accessories		Mbarara-RRH-861	DELL	OPTIPLEX7040	GVBH2F2
Laptop/ Computer with Accessories		Mbarara-RRH-862	DELL	OPTIPLEX7040	GVBH2F2
Laptop/ Computer with Accessories		Mbarara-RRH-863	DELL	OPTIPLEX7040	GVBH2F2
Laptop/ Computer with Accessories		Mbarara-RRH-864	DELL	OPTIPLEX7040	GVBH2F2
Laptop/ Computer with Accessories		Mbarara-RRH-865	DELL	OPTIPLEX7040	GVBH2F2
Laptop/ Computer with Accessories		Mbarara-RRH-866	DELL	OPTIPLEX7040	GVBH2F2
Laptop/ Computer with Accessories		Mbarara-RRH-867	DELL	OPTIPLEX7040	GVBH2F2
Laptop/ Computer with Accessories		Mbarara-RRH-868	DELL	OPTIPLEX7040	GVBH2F2
Laptop/ Computer with Accessories		Mbarara-RRH-869	DELL	OPTIPLEX7040	GVBH2F2
Laptop/ Computer with Accessories		Mbarara-RRH-870	DELL	OPTIPLEX7040	GVBH2F2
Laptop/ Computer with Accessories		Mbarara-RRH-871	DELL	OPTIPLEX7040	GVBH2F2
Laptop/ Computer with Accessories		Mbarara-RRH-872	DELL	OPTIPLEX7040	GVBH2F2
Laptop/ Computer with		Mbarara-RRH-873	DELL	OPTIPLEX7040	GVBH2F2

Equipment Name	Type	System ID	Model Name	Model No.	Serial No.
Accessories					
Laptop/ Computer with Accessories		Mbarara-RRH-874	DELL	OPTIPLEX7040	GVBH2F2
Laptop/ Computer with Accessories		Mbarara-RRH-875	DELL	OPTIPLEX7040	GVBH2F2
Laptop/ Computer with Accessories		Mbarara-RRH-876	DELL	OPTIPLEX7040	GVBH2F2
Laptop/ Computer with Accessories		Mbarara-RRH-877	DELL	OPTIPLEX7040	GVBH2F2
Laptop/ Computer with Accessories		Mbarara-RRH-878	DELL	OPTIPLEX7040	GVBH2F2
Laptop/ Computer with Accessories		Mbarara-RRH-879	DELL	OPTIPLEX7040	GVBH2F2
Laptop/ Computer with Accessories		Mbarara-RRH-880	DELL	OPTIPLEX7040	GVBH2F2
Laptop/ Computer with Accessories		Mbarara-RRH-881	DELL	OPTIPLEX7040	GVBH2F2
Laptop/ Computer with Accessories		Mbarara-RRH-882	DELL	OPTIPLEX7040	GVBH2F2
Laptop/ Computer with Accessories		Mbarara-RRH-883	DELL	OPTIPLEX7040	GVBH2F2
Laptop/ Computer with Accessories		Mbarara-RRH-884	DELL	OPTIPLEX7040	GVBH2F2
Laptop/ Computer with Accessories		Mbarara-RRH-885	DELL	OPTIPLEX7040	GVBH2F2
Laptop/ Computer with Accessories		Mbarara-RRH-886	DELL	OPTIPLEX7040	GVBH2F2
Laptop/ Computer with Accessories		Mbarara-RRH-887	DELL	OPTIPLEX7040	GVBH2F2
Laptop/ Computer with Accessories		Mbarara-RRH-888	DELL	OPTIPLEX7040	GVBH2F2
Laptop/ Computer with Accessories		Mbarara-RRH-889	DELL	OPTIPLEX7040	GVBH2F2
Laptop/ Computer with Accessories		Mbarara-RRH-890	DELL	OPTIPLEX7040	GVBH2F2
Laptop/ Computer with Accessories		Mbarara-RRH-891	DELL	OPTIPLEX7040	GVBH2F2

Equipment Name	Type	System ID	Model Name	Model No.	Serial No.
Laptop/ Computer with Accessories		Mbarara-RRH-892	DELL	OPTIPLEX7040	GVBH2F2
Laptop/ Computer with Accessories		Mbarara-RRH-893	DELL	OPTIPLEX7040	GVBH2F2
Laptop/ Computer with Accessories		Mbarara-RRH-894	DELL	OPTIPLEX7040	GVBH2F2
Laptop/ Computer with Accessories		Mbarara-RRH-1270			JB-97006242
Laryngoscope	ME	Shuuku-HC IV-29512			
Laryngoscope	ME	Kabuyanda-HC IV-27921			
Laundry Trolley 500 Ltr	MF	Shuuku-HC IV-29530			
LCD Projector and Screen	HSS	Mbarara-RRH-1130	EPSON	H838B	X4GB8202157
LCD Projector and Screen	HSS	Mbarara-RRH-1131	EPSON	H976B	X8A215000367
Light, Torch, Rubber coated	HSS	Mbarara-RRH-2747		TCLYJ_052H	YZ06RRDI6
Magnetic Stirrer	ME	Mbarara-RRH-1176	Raypa		68640
Medical Gas Flow Meter	HSS	Mbarara-RRH-1287	EASYVAC	1000	008MXE
Medical Gas Flow Meter	HSS	Mbarara-RRH-1288	EASYVAC	1000	008MXQ
Medical Gas Flow Meter	HSS	Mbarara-RRH-1289	EASYVAC	1000	008MXZ9
Medical Gas Flow Meter	HSS	Mbarara-RRH-1290	EASYVAC	1000	008MXX
Medical Gas Flow Meter	HSS	Mbarara-RRH-1291	EASYVAC	1000	008MX6
Medical Gas Flow Meter	HSS	Mbarara-RRH-1292	EASYVAC	1000	008MXyK
Medical Gas Flow Meter	HSS	Mbarara-RRH-1293	EASYVAC	1000	008MXO
Medical Gas Flow Meter	HSS	Mbarara-RRH-1294	EASYVAC	1000	008MXZ5
Medical Gas Flow Meter	HSS	Mbarara-RRH-1295	EASYVAC	1000	008MXZ5
Medical Gas Flow Meter	HSS	Mbarara-RRH-1296	EASYVAC	1000	008MXZ5
Medical Gas Flow Meter	HSS	Mbarara-RRH-1297	EASYVAC	1000	008MZ0
Medical Gas Flow Meter	HSS	Mbarara-RRH-1298	EASYCARE 02	BS 341	008N05
Medical Gas Flow Meter	HSS	Mbarara-RRH-1299	EASYCARE 02	BS 341	008N08
Medical Gas	HSS	Mbarara-	EASYCARE 02	BS 341	008MZL

Equipment Name	Type	System ID	Model Name	Model No.	Serial No.
Flow Meter		RRH-1300			
Medical Gas Flow Meter	HSS	Mbarara-RRH-1301	FM-02	BS 341	008MZQ
Medical Gas Flow Meter	HSS	Mbarara-RRH-1302	EASYCARE 02	BS 341	008MY7
Medical Gas Flow Meter	HSS	Mbarara-RRH-1303	EASYCARE 02	BS 341	008MZD
Medical Gas Flow Meter	HSS	Mbarara-RRH-1304	EASYCARE 02	BS 341	008MZD
Medical Gas Flow Meter	HSS	Mbarara-RRH-1305	EASYCARE 02	BS 341	008N09
Medical Gas Flow Meter	HSS	Mbarara-RRH-1306	EASYCARE 02	BS 341	008NOL
Medical Gas Flow Meter	HSS	Mbarara-RRH-1307	EASYCARE 02	BS 341	008MYN
Medical Gas Flow Meter	HSS	Mbarara-RRH-1308	EASYCARE 02	BS 341	008MXD
Medical Oxygen Plant	HSS	Mbarara-RRH-993	XUAN LI DA		JB/T10391-2002
Medicine Trolley	MF	Mbarara-RRH-366	NTHM112		NTH1120744
Medicine Trolley	MF	Mbarara-RRH-395			
Medicine Trolley	MF	Mbarara-RRH-397			
Medicine Trolley	MF	Mbarara-RRH-398			
Medicine Trolley	MF	Mbarara-RRH-420			
Medicine Trolley	MF	Mbarara-RRH-457			
Medicine Trolley	MF	Mbarara-RRH-460			
Micro Hematocrit Centrifuge, Electrical	ME	Mbarara-RRH-1416	HEMATASTAT		0905ER11006
Microscope Binocular	ME	Mbarara-RRH-1035	OLYMPUS	CX23LEDFS1	8M87190
Microscope Binocular	ME	Kabwohe-HC IV-29948	CX21FS1		5A92622
Microscope Binocular	ME	Kyabugimbi-HC IV-29547		JH47037	003869
Microscope Binocular	ME	Kyabugimbi-HC IV-29548		CX23LEDRFS1	9L86121
Microscope Binocular	ME	Ishongororo-HC IV-27592		C02X10B	0003649
Microscope Binocular	ME	Ishongororo-HC IV-27594		1130	
Microscope Binocular	ME	Ishongororo-HC IV-27595		CX23 OLYMPUS	9K8711
Microscope Binocular	ME	Ishongororo-HC IV-27613	Olympus	CX21FSI	T5

Equipment Name	Type	System ID	Model Name	Model No.	Serial No.
Microscope Binocular	ME	Ishongororo-HC IV-27614			300370
Microscope Binocular	ME	Kabuyanda-HC IV-27982		XSZ-N207	000414
Microscope Binocular	ME	Kabuyanda-HC IV-27997		CX21FSI	9E82172
Microscope Binocular	ME	Kazo-HC IV-27728		CX21FSI	0F81905
Microscope Binocular	ME	Kiruhura-HC IV-27807	CX22 OLYMPUS		2L80526
Microscope Binocular	ME	Bwizibwera-HC IV-27568	CX 23 OLYMPUS		OJ82659
Microscope, Fluorescent	ME	Mbarara-RRH-570	OLYMPUS	CH30RF200	8M16141
Microscope, Fluorescent	ME	Mbarara-RRH-571	OLYMPUS	CH30RF200	8M16141
Microscope, Fluorescent	ME	Mbarara-RRH-572	OLYMPUS	CH30RF200	8M16141
Microscope, Fluorescent	ME	Mbarara-RRH-573	OLYMPUS	CH30RF200	8M16141
Microscope, Fluorescent	ME	Mbarara-RRH-574	OLYMPUS	CH30RF200	8M16141
Microscope, Fluorescent	ME	Mbarara-RRH-575	OLYMPUS	CH30RF200	8M16141
Microscope, Fluorescent	ME	Mbarara-RRH-576	OLYMPUS	CH30RF200	8M16141
Microscope, Fluorescent	ME	Mbarara-RRH-717		CX21LEDFS1	IC86492
Microscope, Fluorescent	ME	Mbarara-RRH-1013	OLYMPUS	CX2ILEDFSI	6A24409
Microscope, Fluorescent	ME	Mbarara-RRH-1015	OLYMPUS	CX21LEDFS1	1C86492
Microscope, Fluorescent	ME	Mbarara-RRH-1017	OLYMPUS	CX21LEDFSI	1C86491
Microscope, Fluorescent	ME	Mbarara-RRH-1020	OLYMPUS	CX23NEDFSI	5J85923
Microscope, Fluorescent	ME	Mbarara-RRH-1024	OLYMPUS	CX22RFS1	2M82721
Microscope, Fluorescent	ME	Mbarara-RRH-1026	OLYMPUS	CX22RFS1	2L89153
Microscope, Fluorescent	ME	Mbarara-RRH-1028	OPTIKA	B-292PLi	513349
Microscope, Fluorescent	ME	Mbarara-RRH-1030	OLYMPUS	CH30RF200	T3 8M16141
Microscope, Fluorescent	ME	Mbarara-RRH-1032	CARL ZEISS	PRIMO STAR	3133000795
Microscope, Fluorescent	ME	Mbarara-RRH-1252	Olympus CX23	CX23LEDRFS1	OG82289
Microscope, Fluorescent	ME	Mbarara-RRH-1253	Olympus CX23	CX23LEDRFS1	OD82008
Microscope, monocular	ME	Bugamba-HC IV-32394	CX23RTFS2		BE49322
Mobile	ME	Mbarara-	Mindray LED	HYLED 600	50-2B001649

Equipment Name	Type	System ID	Model Name	Model No.	Serial No.
Operating Light		RRH-34055	Lighthead		
Mobile Operating Light	ME	Bugamba-HC IV-30453		KL04L/I	240601107196
Mobile Operating Light	ME	Rugazi-HC IV-28566	SW-YS-10-03LB		22062002-005023
Mobile Operating Light	ME	Rugazi-HC IV-28567	DLR-92		23581
Mobile Operating Light	ME	Kabwohe-HC IV-29812			23702
Mobile Operating Light	ME	Shuuku-HC IV-29502	KL04L.I		ZY0601107197
Mobile Operating Light	ME	Bwizibwera-HC IV-27521	LED 30		LED-30-0237
Mobile Operating Light	ME	Bwizibwera-HC IV-27534			23591
Mobile Operating Light	ME	Bwizibwera-HC IV-27544		SW-KS-Q10-03LB	S/N :22062002-020/023
Mobile Operating Light	ME	Kyabugimbi-HC IV-30340		IP20	071123-42473
Mobile Operating Light	ME	Bushenyi-HC IV-28942	KS -Q10-030		
Mobile Operating Light	ME	Bushenyi-HC IV-29019	YDK05D		150101180203
Mobile Operating Light	ME	Ishongororo-HC IV-27623		300A	208
Mobile Operating Light	ME	Ruhoko-HC IV-27702	CEILING		
Mobile Operating Light	ME	Nyamuyanja-HC IV-27978	300A		133
Mobile Operating Light	ME	Kabuyanda-HC IV-27862	AMAX3030FD		1271/4
Mobile Operating Light	ME	Kazo-HC IV-27752		KL04LIII	
Mobile Operating Light	ME	Kiruhura-HC IV-27821		KL04LI	
Magnetic Resonance Imaging (MRI) Machine	ME	Mbarara-RRH-31669	Somatom	10840816	182217
Nebulizer, Compressed Air Jet	ME	Shuuku-HC IV-29525	JLN-2306AS		180500149
Nebulizer, Compressed Air Jet	ME	Ishongororo-HC IV-27600		CWAN-2	201960987
Nebulizer, Compressed Air Jet	ME	Mbarara-RRH-1260	Porta neb		10AG930105
Nebulizer, Compressed Air Jet	ME	Mbarara-RRH-458	OMRON NE-C900		20200500165UF
Nebulizer, Compressed Air Jet	ME	Mbarara-RRH-466	OMRON NE-C900		20200500168UF

Equipment Name	Type	System ID	Model Name	Model No.	Serial No.
Nebulizer, Compressed Air Jet	ME	Mbarara-RRH-469	OMRON NE-C900		20200500167UF
Nebulizer, Compressed Air Jet	ME	Mbarara-RRH-481	OMRON NE-C900		20200500166UF
Nebulizer, Compressed Air Jet	ME	Mbarara-RRH-528		NE-C900	20200500166UF
Nebulizer, Compressed Air Jet	ME	Mbarara-RRH-51784	Classic	ABOX	66321FX09
Cold Light Source	ME	Mbarara-RRH-389		A/06230VAC	
Cold Light Source	ME	Mbarara-RRH-414		MERILUX X3	101119-82142
Operating Microscope Orthopedics	ME	Ishongororo-HC IV-27593		1130	
Operating Table, Electric		Ruhoko-HC IV-27701		3001A	
Operating Table, Electric		Mbarara-RRH-405		SURGICAL2080MIA	0434576010
Operating Table, Electric		Mbarara-RRH-412		SURGICAL2080	0429780045
Operating Table, Electric		Mbarara-RRH-470		2080	0411186042
Operating Table, Manual		Bwizibwera-HC IV-27522	SW-2T-OT-M3H		2020023-EOTM3H-011
Operating Table, Manual		Kyabugimbi-HC IV-30357			
Operating Table, Manual		Bushenyi-HC IV-28945	3008A		RHYL
Operating Table, Manual		Bushenyi-HC IV-28998	3008A		RHYL
Operating Table, Manual		Bushenyi-HC IV-29013	3008A		
Operating Table, Manual		Ishongororo-HC IV-27619			
Operating Table, Manual		Ishongororo-HC IV-27628		1650	
Operating Table, Manual		Kabuyanda-HC IV-27861	TECHNOMED 30		
Operating Table, Manual		Kazo-HC IV-27758		3008AB	
Operating Table, Manual		Kinoni-HC III-28066		1650	
Operating Table, Manual		Bugamba-HC IV-32320		3008B	150602101
Operating Table, Manual		Rugazi-HC IV-28894	M30		
Operating Table, Manual		Rugazi-HC IV-28895	SW-ZT-OT-M3H		20220623-E-OTM3H-008

Equipment Name	Type	System ID	Model Name	Model No.	Serial No.
Operating Table, Manual		Rugazi-HC IV-28920	ROT-160		ROT-160-13017
Operating Table, Manual		Rugazi-HC IV-28921	ROT-160		ROT-160-13017
Operating Table, Manual		Shuuku-HC IV-29538	3008B		750602101221
Operation Lamp, Ceiling Mounted	ME	Mbarara-RRH-428		MERILUX X3	
Operation Lamp, Ceiling Mounted	ME	Mbarara-RRH-450		MERILUX X3	
Operation Lamp, Ceiling Mounted	ME	Mbarara-RRH-467		MERILUX X3	101119-82145
Operation Lamp, Ceiling Mounted	ME	Mbarara-RRH-468		MERILUX X3	101119-145
Oxygen Concentrator	ME	Mbarara-RRH-367	OXGYEN- TECH	5S	20206770
Oxygen Concentrator	ME	Mbarara-RRH-396	Kro2:00		10T20030592
Oxygen Concentrator	ME	Mbarara-RRH-400	Krober O2	KrO2:00	10T20030593
Oxygen Concentrator	ME	Mbarara-RRH-402	Krober O2	KrO2:00	10T20030587
Oxygen Concentrator	ME	Mbarara-RRH-404	Krober O2	KrO2:00	10T20030586
Oxygen Concentrator	ME	Mbarara-RRH-407	Krober O2	KrO2:00	10T20030589
Oxygen Concentrator	ME	Mbarara-RRH-408	Krober O2	KrO2:00	10T20030590
Oxygen Concentrator	ME	Mbarara-RRH-413	Newlife intensity 10		CBB012029050
Oxygen Concentrator	ME	Mbarara-RRH-415	Newlife intensity 10		CBB0120290907
Oxygen Concentrator	ME	Mbarara-RRH-493		525KD-333REVA	B209020155KJ
Oxygen Concentrator	ME	Mbarara-RRH-496		JAY-5	MZJ5D37942
Oxygen Concentrator	ME	Mbarara-RRH-507		AS099-210	CBB0121020809
Oxygen Concentrator	ME	Mbarara-RRH-515	Newlife intensity 10		CBB0121020726
Oxygen Concentrator	ME	Mbarara-RRH-518	Newlife intensity 10		CBB0121020872
Oxygen Concentrator	ME	Mbarara-RRH-521	Newlife intensity 10		CBB0121020873
Oxygen Concentrator	ME	Mbarara-RRH-523	New life intensity 10		CBB0121020872
Oxygen Concentrator	ME	Mbarara-RRH-524	New life intensity 10		CBB0121020879
Oxygen Concentrator	ME	Mbarara-RRH-538		A5099-210	CBB0121020837
Oxygen	ME	Mbarara-		OLV-5	L807081

Equipment Name	Type	System ID	Model Name	Model No.	Serial No.
Concentrator		RRH-540			
Oxygen Concentrator	ME	Mbarara-RRH-541		525KS	B209090366KS
Oxygen Concentrator	ME	Mbarara-RRH-546	Newlife intensity 10		CBB0121020679
Oxygen Concentrator	ME	Mbarara-RRH-548	Newlife intensity 10		CBB0121020723
Oxygen Concentrator	ME	Mbarara-RRH-549	Newlife intensity 10		CBB0121020809
Oxygen Concentrator	ME	Mbarara-RRH-550	Newlife intensity 10		CBB0121020719
Oxygen Concentrator	ME	Mbarara-RRH-563	KROBER02	KRO200	IOT20030590
Oxygen Concentrator	ME	Mbarara-RRH-565	KROBER02	KRO200	IOT20030592
Oxygen Concentrator	ME	Mbarara-RRH-585	JAY-5		MZJ5D37942
Oxygen Concentrator	ME	Mbarara-RRH-593	JAY-5		MZJ5D37937
Oxygen Concentrator	ME	Mbarara-RRH-602		V5-WN-NS	2249071062821
Oxygen Concentrator	ME	Mbarara-RRH-604		525KS	B209020176KS
Oxygen Concentrator	ME	Mbarara-RRH-616		AS099-210	CBB0121020679
Oxygen Concentrator	ME	Mbarara-RRH-621		JAY-5	M2J5D37939
Oxygen Concentrator	ME	Mbarara-RRH-626	NEW LIFE INTENSITY 10		LA422-2C
Oxygen Concentrator	ME	Mbarara-RRH-627	JAY-5		MZJ5D37943
Oxygen Concentrator	ME	Mbarara-RRH-628	JAY-5		MZJ5D37943
Oxygen Concentrator	ME	Mbarara-RRH-629	JAY-5		MZJ5D37943
Oxygen Concentrator	ME	Mbarara-RRH-630	JAY-5		MZJ5D37943
Oxygen Concentrator	ME	Mbarara-RRH-631	JAY-5		MZJ5D37943
Oxygen Concentrator	ME	Mbarara-RRH-632	JAY-5		MZJ5D37943
Oxygen Concentrator	ME	Mbarara-RRH-633	JAY-5		MZJ5D37943

Equipment Name	Type	System ID	Model Name	Model No.	Serial No.
Oxygen Concentrator	ME	Mbarara-RRH-634	JAY-5		MZJ5D37943
Oxygen Concentrator	ME	Mbarara-RRH-635	JAY-5		MZJ5D37943
Oxygen Concentrator	ME	Mbarara-RRH-636	JAY-5		MZJ5D37943
Oxygen Concentrator	ME	Mbarara-RRH-637	JAY-5		MZJ5D37943
Oxygen Concentrator	ME	Mbarara-RRH-638	JAY-5		MZJ5D37943
Oxygen Concentrator	ME	Mbarara-RRH-639	JAY-5		MZJ5D37943
Oxygen Concentrator	ME	Mbarara-RRH-640	JAY-5		MZJ5D37943
Oxygen Concentrator	ME	Mbarara-RRH-641	JAY-5		MZJ5D37943
Oxygen Concentrator	ME	Mbarara-RRH-642	JAY-5		MZJ5D37943
Oxygen Concentrator	ME	Mbarara-RRH-643	JAY-5		MZJ5D37943
Oxygen Concentrator	ME	Mbarara-RRH-644	JAY-5		MZJ5D37943
Oxygen Concentrator	ME	Mbarara-RRH-645	JAY-5		MZJ5D37943
Oxygen Concentrator	ME	Mbarara-RRH-646	JAY-5		MZJ5D37943
Oxygen Concentrator	ME	Mbarara-RRH-647	JAY-5		MZJ5D37943
Oxygen Concentrator	ME	Mbarara-RRH-648	JAY-5		MZJ5D37943
Oxygen Concentrator	ME	Mbarara-RRH-649	JAY-5		MZJ5D37943
Oxygen Concentrator	ME	Mbarara-RRH-650	JAY-5		MZJ5D37943
Oxygen Concentrator	ME	Mbarara-RRH-651	JAY-5		MZJ5D37943
Oxygen	ME	Mbarara-	JAY-5		MZJ5D37943

Equipment Name	Type	System ID	Model Name	Model No.	Serial No.
Concentrator		RRH-652			
Oxygen Concentrator	ME	Mbarara-RRH-653	JAY-5		MZJ5D37943
Oxygen Concentrator	ME	Mbarara-RRH-654	JAY-5		MZJ5D37943
Oxygen Concentrator	ME	Mbarara-RRH-655	JAY-5		MZJ5D37943
Oxygen Concentrator	ME	Mbarara-RRH-656	JAY-5		MZJ5D37943
Oxygen Concentrator	ME	Mbarara-RRH-657	JAY-5		MZJ5D37943
Oxygen Concentrator	ME	Mbarara-RRH-658	JAY-5		MZJ5D37943
Oxygen Concentrator	ME	Mbarara-RRH-659	JAY-5		MZJ5D37943
Oxygen Concentrator	ME	Mbarara-RRH-660	JAY-5		MZJ5D37943
Oxygen Concentrator	ME	Mbarara-RRH-661	JAY-5		MZJ5D37943
Oxygen Concentrator	ME	Mbarara-RRH-662	JAY-5		MZJ5D37943
Oxygen Concentrator	ME	Mbarara-RRH-663	JAY-5		MZJ5D37943
Oxygen Concentrator	ME	Mbarara-RRH-664	JAY-5		MZJ5D37943
Oxygen Concentrator	ME	Mbarara-RRH-665	JAY-5		MZJ5D37943
Oxygen Concentrator	ME	Mbarara-RRH-666	JAY-5		MZJ5D37943
Oxygen Concentrator	ME	Mbarara-RRH-667	JAY-5		MZJ5D37943
Oxygen Concentrator	ME	Mbarara-RRH-668	JAY-5		MZJ5D37943
Oxygen Concentrator	ME	Mbarara-RRH-669	JAY-5		MZJ5D37943
Oxygen Concentrator	ME	Mbarara-RRH-670	JAY-5		MZJ5D37943

Equipment Name	Type	System ID	Model Name	Model No.	Serial No.
Oxygen Concentrator	ME	Mbarara-RRH-671	JAY-5		MZJ5D37943
Oxygen Concentrator	ME	Mbarara-RRH-672	JAY-5		MZJ5D37943
Oxygen Concentrator	ME	Mbarara-RRH-673	JAY-5		MZJ5D37943
Oxygen Concentrator	ME	Mbarara-RRH-674	JAY-5		MZJ5D37943
Oxygen Concentrator	ME	Mbarara-RRH-675	JAY-5		MZJ5D37943
Oxygen Concentrator	ME	Mbarara-RRH-676	JAY-5		MZJ5D37943
Oxygen Concentrator	ME	Mbarara-RRH-677	JAY-5		MZJ5D37943
Oxygen Concentrator	ME	Mbarara-RRH-678	JAY-5		MZJ5D37943
Oxygen Concentrator	ME	Mbarara-RRH-679	JAY-5		MZJ5D37943
Oxygen Concentrator	ME	Mbarara-RRH-680	JAY-5		MZJ5D37943
Oxygen Concentrator	ME	Mbarara-RRH-681	JAY-5		MZJ5D37943
Oxygen Concentrator	ME	Mbarara-RRH-682	JAY-5		MZJ5D37943
Oxygen Concentrator	ME	Mbarara-RRH-683	JAY-5		MZJ5D37943
Oxygen Concentrator	ME	Mbarara-RRH-684	JAY-5		MZJ5D37943
Oxygen Concentrator	ME	Mbarara-RRH-685	JAY-5		MZJ5D37943
Oxygen Concentrator	ME	Mbarara-RRH-686	JAY-5		MZJ5D37943
Oxygen Concentrator	ME	Mbarara-RRH-687	JAY-5		MZJ5D37943
Oxygen Concentrator	ME	Mbarara-RRH-688	JAY-5		MZJ5D37943

Equipment Name	Type	System ID	Model Name	Model No.	Serial No.
Oxygen Concentrator	ME	Mbarara-RRH-689	JAY-5		MZJ5D37943
Oxygen Concentrator	ME	Mbarara-RRH-690	JAY-5		MZJ5D37943
Oxygen Concentrator	ME	Mbarara-RRH-691	JAY-5		MZJ5D37943
Oxygen Concentrator	ME	Mbarara-RRH-692	JAY-5		MZJ5D37943
Oxygen Concentrator	ME	Mbarara-RRH-693	JAY-5		MZJ5D37943
Oxygen Concentrator	ME	Mbarara-RRH-694	JAY-5		MZJ5D37943
Oxygen Concentrator	ME	Mbarara-RRH-695	JAY-5		MZJ5D37943
Oxygen Concentrator	ME	Mbarara-RRH-696	JAY-5		MZJ5D37943
Oxygen Concentrator	ME	Mbarara-RRH-697	JAY-5		MZJ5D37943
Oxygen Concentrator	ME	Mbarara-RRH-698	JAY-5		MZJ5D37943
Oxygen Concentrator	ME	Mbarara-RRH-699	JAY-5		MZJ5D37943
Oxygen Concentrator	ME	Mbarara-RRH-700	JAY-5		MZJ5D37943
Oxygen Concentrator	ME	Mbarara-RRH-701	JAY-5		MZJ5D37943
Oxygen Concentrator	ME	Mbarara-RRH-702	JAY-5		MZJ5D37943
Oxygen Concentrator	ME	Mbarara-RRH-703	JAY-5		MZJ5D37943
Oxygen Concentrator	ME	Mbarara-RRH-704	JAY-5		MZJ5D37943
Oxygen Concentrator	ME	Mbarara-RRH-705	JAY-5		MZJ5D37943
Oxygen Concentrator	ME	Mbarara-RRH-706	JAY-5		MZJ5D37943
Oxygen	ME	Mbarara-	JAY-5		MZJ5D37943

Equipment Name	Type	System ID	Model Name	Model No.	Serial No.
Concentrator		RRH-707			
Oxygen Concentrator	ME	Mbarara-RRH-708	JAY-5		MZJ5D37943
Oxygen Concentrator	ME	Mbarara-RRH-709	JAY-5		MZJ5D37943
Oxygen Concentrator	ME	Mbarara-RRH-710	JAY-5		MZJ5D37943
Oxygen Concentrator	ME	Mbarara-RRH-711	JAY-5		MZJ5D37943
Oxygen Concentrator	ME	Mbarara-RRH-712	JAY-5		MZJ5D37943
Oxygen Concentrator	ME	Mbarara-RRH-713	JAY-5		MZJ5D37943
Oxygen Concentrator	ME	Mbarara-RRH-714	JAY-5		MZJ5D37943
Oxygen Concentrator	ME	Mbarara-RRH-715	JAY-5		MZJ5D37943
Oxygen Concentrator	ME	Mbarara-RRH-716	NEW LIFE INTENSITY 10		CBB0121020809
Oxygen Concentrator	ME	Mbarara-RRH-722	JAY-5		IP 21 MZJ5D37942
Oxygen Concentrator	ME	Mbarara-RRH-723	JAY-5		MZJ5D37937
Oxygen Concentrator	ME	Mbarara-RRH-725	JAY-5		MZJ5D37938
Oxygen Concentrator	ME	Mbarara-RRH-726	JAY-5		MZJ5D37939
Oxygen Concentrator	ME	Mbarara-RRH-728	JAY-5		MZJ5D37943
Oxygen Concentrator	ME	Bugamba-HC IV-30426	New life		N5130183
Oxygen Concentrator	ME	Bugamba-HC IV-32386	Krober O2		1110073849
Oxygen Concentrator	ME	Rugazi-HC IV-28556	OLV-5		12106013
Oxygen Concentrator	ME	Rugazi-HC IV-28913	KROBER		72117570
Oxygen Concentrator	ME	Kabwohe-HC IV-29842		525KS	R043811K
Oxygen Concentrator	ME	Shuuku-HC IV-29483			R043803KS

Equipment Name	Type	System ID	Model Name	Model No.	Serial No.
Oxygen Concentrator	ME	Shuuku-HC IV-29484	Krober o2		1110073850
Oxygen Concentrator	ME	Kyabugimbi-HC IV-30082	New life		N5063702
Oxygen Concentrator	ME	Bushenyi-HC IV-29006	JAY-5		MZJ5031671
Oxygen Concentrator	ME	Ishongororo-HC IV-27599		OZ-5-01GWO	W20210702950
Oxygen Concentrator	ME	Ruhoko-HC IV-27684	Newlife intensity 10		M5130260
Oxygen Concentrator	ME	Ruhoko-HC IV-27716		D02-5EH	DM210719002
Oxygen Concentrator	ME	Nyamuyanja-HC IV-27999	Newlife		838147
Oxygen Concentrator	ME	Kabuyanda-HC IV-27876	V5-WN-NS		2249135062821
Oxygen Concentrator	ME	Kabuyanda-HC IV-27902	Newlife intensity 10		CBB0121130146
Oxygen Concentrator	ME	Kabuyanda-HC IV-27956	JAY-10		MZJ10517191
Oxygen Concentrator	ME	Kiruhura-HC IV-27824	Krober O2		1110073851
Oxygen Concentrator	ME	Mbarara-RRH-1259	AIR SEP	New life intensity 10	CBB0117430115
Oxygen Concentrator	ME	Mbarara-RRH-1271		Jay-5	Mzj5d29362
Oxygen Concentrator	ME	Mbarara-RRH-1279	JAY-5		MZJ5S17426
Oxygen Concentrator	ME	Mbarara-RRH-1281	JAY-5		MZJ5D37939
Oxygen Concentrator	ME	Mbarara-RRH-1282	JAY-5		MZJ5D37938
Oxygen Concentrator	ME	Mbarara-RRH-1283	V5-WN-NS		2249071062821
Oxygen Regulator		Mbarara-RRH-25249			
Oxygen Regulator		Mbarara-RRH-25253			
Oxygen Regulator		Mbarara-RRH-25255			
Oxygen Regulator		Ruhoko-HC IV-27707			41063121
Oxygen Therapy Apparatus with 40Lt. Cylinder	ME	Bushenyi-HC IV-29021			
Oxygen Therapy Apparatus with 40Lt. Cylinder	ME	Bushenyi-HC IV-29025			
Oxygen Therapy Apparatus with 40Lt. Cylinder	ME	Nyamuyanja-HC IV-28003			
Oxygen Therapy Apparatus with	ME	Rugazi-HC IV-28555			

Equipment Name	Type	System ID	Model Name	Model No.	Serial No.
40Lt. Cylinder					
Oxygen Therapy Apparatus with 40Lt. Cylinder	ME	Rugazi-HC IV-28898			
Oxygen Therapy Apparatus with 40Lt. Cylinder	ME	Rugazi-HC IV-28916			
Oxygen Therapy Apparatus with 40Lt. Cylinder	ME	Rugazi-HC IV-28927			
Oxygen Therapy Apparatus with 40Lt. Cylinder	ME	Shuuku-HC IV-29489			
Oxygen Therapy Apparatus with 40Lt. Cylinder	ME	Shuuku-HC IV-29531			
Oxygen Therapy Apparatus with 40Lt. Cylinder	ME	Shuuku-HC IV-29748			
Oxygen Therapy Apparatus with 40Lt. Cylinder	ME	Shuuku-HC IV-29749			
Patient Monitor, Portable with Roller Stand	ME	Mbarara-RRH-2778	CARE SCAPE	V100	SH617280057SA
Patient Monitor, Portable with Roller Stand	ME	Mbarara-RRH-31664	M12		M76B0000748
Patient Monitor, Portable with Roller Stand	ME	Mbarara-RRH-31665	Q5		Q51D0003903
Patient Monitor, Portable with Roller Stand	ME	Mbarara-RRH-31673	Elite V5		M22C19820021,M22C19820011
Patient Monitor, Portable with Roller Stand	ME	Mbarara-RRH-34104	Elite V8		002338-M22C 19820014
Patient Monitor, Portable with Roller Stand	ME	Mbarara-RRH-34105	Elite V5		002338-M22C 19820011
Patient Monitor, Portable with Roller Stand	ME	Mbarara-RRH-34106	Elite V8		002338-M22C 19820021
Patient Monitor, Portable with Roller Stand	ME	Mbarara-RRH-1265	Bene vision Nis	M	F5-97007495
Patient Monitor, Portable with Roller Stand	ME	Mbarara-RRH-1285	Hamilton-C2		8359
Patient Monitor, Portable with Roller Stand	ME	Mbarara-RRH-1425	BENEVIEW T6		CG-0B101035
Patient Monitor, Portable with Roller Stand	ME	Bwizibwera-HC IV-27532	M9000A		M013E007194

Equipment Name	Type	System ID	Model Name	Model No.	Serial No.
Patient Monitor, Portable with Roller Stand	ME	Mbarara-RRH-421	UMECIO Mindray		KN-OC088319
Patient Monitor, Portable with Roller Stand	ME	Mbarara-RRH-423	UMECIO Mindray		KN-OC088302
Patient Monitor, Portable with Roller Stand	ME	Mbarara-RRH-426	BENEVIEW T6		CG-0BIO1041
Patient Monitor, Portable with Roller Stand	ME	Mbarara-RRH-455		DASH 4000	SD009037297GA
Patient Monitor, Portable with Roller Stand	ME	Mbarara-RRH-456		Q6100F-PA0D219	PV05475-HO
Patient Monitor, Portable with Roller Stand	ME	Mbarara-RRH-461	UMECIO Mindray		KN-OC088294
Patient Monitor, Portable with Roller Stand	ME	Mbarara-RRH-463		BENE VIEW T6	CG-OB101034
Patient Monitor, Portable with Roller Stand	ME	Mbarara-RRH-502		MD9012	MD1491389
Patient Monitor, Portable with Roller Stand	ME	Mbarara-RRH-517		MD9012	MD1491398
Patient Monitor, Portable with Roller Stand	ME	Mbarara-RRH-539		MD9012	MD901219G240024
Patient Monitor, Portable with Roller Stand	ME	Mbarara-RRH-542		CMS9000	21031100005
Patient Monitor, Portable with Roller Stand	ME	Mbarara-RRH-557	UMECIO		KN-0C088284
Patient Monitor, Portable with Roller Stand	ME	Mbarara-RRH-595		MD9012	
Patient Monitor, Portable with Roller Stand	ME	Mbarara-RRH-596		MD9012	
Patient Monitor, Portable with Roller Stand	ME	Mbarara-RRH-597		MD9012	
Patient Monitor, Portable with Roller Stand	ME	Mbarara-RRH-605	CON TEC CMS 700		20120600094
Patient Monitor, Portable with Roller Stand	ME	Mbarara-RRH-611		CMS7000	20120600060
Patient Monitor, Portable with Roller Stand	ME	Ishongororo-HC IV-27626		EDAN X10	261590-MZ1B12340038
Patient Monitor,	ME	Ruhoko-HC		X10	261590-M2270409003

Equipment Name	Type	System ID	Model Name	Model No.	Serial No.
Portable with Roller Stand		IV-27697			
Patient Monitor, Portable with Roller Stand	ME	Nyamuyanja-HC IV-28021		VS-600	FU-24032047
Patient Monitor, Portable with Roller Stand	ME	Kabuyanda-HC IV-27860	DASH	2500	SCG0741383810A
Patient Monitor, Portable with Roller Stand	ME	Kabuyanda-HC IV-27888	CADIOCAP/5		6473347
Patient Monitor, Portable with Roller Stand	ME	Kabuyanda-HC IV-27920	EDAN	IM60	360079-M20801570003
Patient Monitor, Portable with Roller Stand	ME	Kiruhura-HC IV-27823	EDAN	X10	261590-M20704780023
Patient Monitor, Wall/Bed/Table top Mounted	ME	Kyabugimbi-HC IV-30348	XY10		261590-M21606430008
Patient Monitor, Wall/Bed/Table top Mounted	ME	Bushenyi-HC IV-29005	M9000A		MO13E008979
Patient Monitor, Wall/Bed/Table top Mounted	ME	Kabuyanda-HC IV-27985			
Patient Monitor, Wall/Bed/Table top Mounted	ME	Mbarara-RRH-989	M3A		360125-M21318150005
Patient Monitor, Wall/Bed/Table top Mounted	ME	Mbarara-RRH-991	M3A		360125-M21318150034
Patient Monitor, Wall/Bed/Table top Mounted	ME	Mbarara-RRH-994	M3A		360125-M21318150021
Patient Monitor, Wall/Bed/Table top Mounted	ME	Mbarara-RRH-996	IM8B		360001-M21318090001
Patient Monitor, Wall/Bed/Table top Mounted	ME	Mbarara-RRH-999	M3A		360125-M213181500021
Patient Monitor, Wall/Bed/Table top Mounted	ME	Mbarara-RRH-1001	M3A		360125-M21318150009
Patient Monitor, Wall/Bed/Table top Mounted	ME	Mbarara-RRH-1003	M3A		360125-M21318150036
Patient Monitor, Wall/Bed/Table top Mounted	ME	Mbarara-RRH-1006	M3A		360125-M21318150032
Patient Monitor, Wall/Bed/Table top Mounted	ME	Mbarara-RRH-1008	M3A		360125-M213181500008
Patient Monitor, Wall/Bed/Table	ME	Mbarara-RRH-1009			360125-M21318150040

Equipment Name	Type	System ID	Model Name	Model No.	Serial No.
top Mounted					
Patient Monitor, Wall/Bed/Table top Mounted	ME	Mbarara-RRH-1011	M3A		360125-M21318150027
Patient Monitor, Wall/Bed/Table top Mounted	ME	Mbarara-RRH-1012	M3A		360125-M21318150017
Patient Monitor, Wall/Bed/Table top Mounted	ME	Mbarara-RRH-1014			360125-M213181500002
Patient Monitor, Wall/Bed/Table top Mounted	ME	Mbarara-RRH-1016	M3A		360125-M21318150011
Patient Monitor, Wall/Bed/Table top Mounted	ME	Mbarara-RRH-1018	M3A		360125-M19705440003-01
Patient Monitor, Wall/Bed/Table top Mounted	ME	Mbarara-RRH-1019	M3A		360125-M21318150037
Patient Monitor, Wall/Bed/Table top Mounted	ME	Mbarara-RRH-1021	M3A		360125-M19705440005-01
Patient Monitor, Wall/Bed/Table top Mounted	ME	Mbarara-RRH-1022	M3A		360125-M213181500003
Patient Monitor, Wall/Bed/Table top Mounted	ME	Mbarara-RRH-1023	M3A		360125-M213181500028
Patient Monitor, Wall/Bed/Table top Mounted	ME	Mbarara-RRH-1025	M3A		360125-M213181500024
Patient Monitor, Wall/Bed/Table top Mounted	ME	Mbarara-RRH-1027	IM8B		360001-M21318090006
Patient Monitor, Wall/Bed/Table top Mounted	ME	Mbarara-RRH-1029	IM8B		360001-M21318090005
Patient Monitor, Wall/Bed/Table top Mounted	ME	Mbarara-RRH-1031	IM8B		360125-M21318090005
Patient Monitor, Wall/Bed/Table top Mounted	ME	Mbarara-RRH-1033	IM8B		360001-M21318090008
Patient Monitor, Wall/Bed/Table top Mounted	ME	Mbarara-RRH-1038	CMS5100		21041000013
Patient Monitor, Wall/Bed/Table top Mounted	ME	Mbarara-RRH-1040	CMS5100		21041000005
Patient Monitor, Wall/Bed/Table top Mounted	ME	Mbarara-RRH-1042			21041000001
Patient Monitor, Wall/Bed/Table top Mounted	ME	Mbarara-RRH-1044	CMS5100		21041000009

Equipment Name	Type	System ID	Model Name	Model No.	Serial No.
Patient Monitor, Wall/Bed/Table top Mounted	ME	Mbarara-RRH-1046	CMS5100		21041000004
Patient Monitor, Wall/Bed/Table top Mounted	ME	Mbarara-RRH-1048	CMS5100		21041000042
Patient Monitor, Wall/Bed/Table top Mounted	ME	Mbarara-RRH-1309	EDAN	X12	261594-M205084700
Patient Monitor, Wall/Bed/Table top Mounted	ME	Mbarara-RRH-1310	EDAN	X12	261594-M20508470028
Patient Monitor, Wall/Bed/Table top Mounted	ME	Mbarara-RRH-1312	MINDAY	Beneview T6	CG-0B101033
Patient Monitor, Wall/Bed/Table top Mounted	ME	Mbarara-RRH-1423			CG-0B101039
Patient Monitor, Wall/Bed/Table top Mounted	ME	Mbarara-RRH-2488		M-8000E	MD1491002
Patient Monitor, Wall/Bed/Table top Mounted	ME	Mbarara-RRH-2496		X12	261594-M20508470060
Patient Monitor, Wall/Bed/Table top Mounted	ME	Mbarara-RRH-2501		X12	261594-M20508470052
Patient Monitor, Wall/Bed/Table top Mounted	ME	Mbarara-RRH-2743	BENEVIEW T6	6801B_CTO_SO1	GC _OB101031
Patient Monitor, Wall/Bed/Table top Mounted	ME	Kinoni-HC IV-28047		M9000A	M013E008568
Patient Monitor, Wall/Bed/Table top Mounted	ME	Bugamba-HC IV-32328		X10	261590-M2112340036
Patient Monitor, Wall/Bed/Table top Mounted	ME	Rugazi-HC IV-28571	X10		261590-M21B12340007
Patient Monitor, Wall/Bed/Table top Mounted	ME	Mbarara-RRH-446	UMECIO Mindray		KN-OC088308
Patient Monitor, Wall/Bed/Table top Mounted	ME	Mbarara-RRH-451	UMECIO Mindray		KN-OC088280
Patient Monitor, Wall/Bed/Table top Mounted	ME	Mbarara-RRH-453	UMECIO Mindray		KN-OC088273
Patient Monitor, Wall/Bed/Table top Mounted	ME	Mbarara-RRH-454	UMECIO Mindray		KN-OC088297
Patient Monitor, Wall/Bed/Table top Mounted	ME	Mbarara-RRH-473	Mindray		KN-OC088284
Patient Monitor,	ME	Mbarara-	Mindray		KN-OC088289

Equipment Name	Type	System ID	Model Name	Model No.	Serial No.
Wall/Bed/Table top Mounted		RRH-478			
Patient Monitor, Wall/Bed/Table top Mounted	ME	Mbarara-RRH-511	M3A		360125-M21318150022
Patient Monitor, Wall/Bed/Table top Mounted	ME	Mbarara-RRH-589	MD9012		MD1491393
Patient Monitor, Wall/Bed/Table top Mounted	ME	Mbarara-RRH-594	MD9012		MD1491390
Patient Monitor, Wall/Bed/Table top Mounted	ME	Mbarara-RRH-599	MD9012		MD1491397
Patient Monitor, Wall/Bed/Table top Mounted	ME	Mbarara-RRH-601	MD9012		MD1491397
Patient Monitor, Wall/Bed/Table top Mounted	ME	Mbarara-RRH-603	CON TEC CMS 700		20120600060
Patient Monitor, Wall/Bed/Table top Mounted	ME	Mbarara-RRH-607	CON TEC CMS 700		20120600098
Patient Monitor, Wall/Bed/Table top Mounted	ME	Mbarara-RRH-609			MD1491398
Patient Monitor, Wall/Bed/Table top Mounted	ME	Mbarara-RRH-612	CMS9000		BSEA100143
Patient Monitor, Wall/Bed/Table top Mounted	ME	Mbarara-RRH-614	MD9012		MD9012199G240024
Patient Monitors, Central Monitoring System	ME	Mbarara-RRH-368	BENEVIEW	T6	CG-OB101040
Patient Monitors, Central Monitoring System	ME	Mbarara-RRH-370	BENEVIEW	T6	CG-08101037
Patient Monitors, Central Monitoring System	ME	Mbarara-RRH-373	BENEVIEW	T6	
Patient Monitors, Central Monitoring System	ME	Mbarara-RRH-374	BENEVIEW	T6	
Patient Monitors, Central Monitoring System	ME	Mbarara-RRH-1268	Del	E1916Hu	CN-07cxPR-FCC00-915-Dv2u

Equipment Name	Type	System ID	Model Name	Model No.	Serial No.
Patient Monitors, Central Monitoring System	ME	Mbarara-RRH-1269	Del	E1916Hu	CN-07cxPR-FCC00-915-Dv2u
Patient Screen	MF	Kyabugimbi-HC IV-29522			
Patient Screen	MF	Kyabugimbi-HC IV-29527			
Patient Screen	MF	Kyabugimbi-HC IV-30143			
Patient Screen	MF	Kyabugimbi-HC IV-30147			
Patient Screen	MF	Kyabugimbi-HC IV-30149			
Patient Screen	MF	Kyabugimbi-HC IV-30150			
Patient Screen	MF	Kyabugimbi-HC IV-30334			
Patient Screen	MF	Kyabugimbi-HC IV-30338			
Patient Screen	MF	Kyabugimbi-HC IV-30370			
Patient Screen	MF	Kyabugimbi-HC IV-30410			
Patient Screen	MF	Kyabugimbi-HC IV-30412			
Patient Screen	MF	Bushenyi-HC IV-29020			
Patient Screen	MF	Ruhoko-HC IV-27713	CASTOS		
Patient Screen	MF	Ruhoko-HC IV-27714	CASTOS		
Patient Screen	MF	Nyamuyanja-HC IV-28034			
Patient Screen	MF	Nyamuyanja-HC IV-28036			
Patient Screen	MF	Bugamba-HC IV-32421			
Patient Screen	MF	Kabwohe-HC IV-29815			
Patient Screen	MF	Kabwohe-HC IV-29841			
Patient Screen	MF	Kabwohe-HC IV-29862			
Patient Screen	MF	Shuuku-HC IV-29500			
Patient Screen	MF	Shuuku-HC IV-30530			
Patient Stretcher/Trolley	MF	Kinoni-HC IV-28068			
Patient Stretcher/Trolley	MF	Kinoni-HC IV-28070			

Equipment Name	Type	System ID	Model Name	Model No.	Serial No.
Patient Stretcher/Trolley	MF	Bugamba-HC IV-30443			
Patient Stretcher/Trolley	MF	Bugamba-HC IV-32387			
Patient Stretcher/Trolley	MF	Rugazi-HC IV-28917	SW-ZT-PT-01		20220623-EPTO1-022
Patient Stretcher/Trolley	MF	Rugazi-HC IV-28928			
Patient Stretcher/Trolley	MF	Rugazi-HC IV-28932			
Patient Stretcher/Trolley	MF	Rugazi-HC IV-28941			
Patient Stretcher/Trolley	MF	Kabwohe-HC IV-29794			
Patient Stretcher/Trolley	MF	Kabwohe-HC IV-29796			
Patient Stretcher/Trolley	MF	Kabwohe-HC IV-29816			
Patient Stretcher/Trolley	MF	Shuuku-HC IV-29494			
Patient Stretcher/Trolley	MF	Shuuku-HC IV-29521			
Patient Stretcher/Trolley	MF	Kiruhura-HC IV-27833			
Patient Stretcher/Trolley	MF	Kiruhura-HC IV-27835			
Patient Stretcher/Trolley	MF	Mbarara-RRH-556			
Patient Stretcher/Trolley	MF	Mbarara-RRH-559			
Patient Stretcher/Trolley	MF	Mbarara-RRH-568			
Patient Stretcher/Trolley	MF	Kyabugimbi-HC IV-30362			
Patient Stretcher/Trolley	MF	Kyabugimbi-HC IV-30363			
Patient Stretcher/Trolley	MF	Bushenyi-HC IV-29017			
Patient Stretcher/Trolley	MF	Bushenyi-HC IV-29026			
Patient Stretcher/Trolley	MF	Ishongororo-HC IV-27602			
Patient Stretcher/Trolley	MF	Ruhoko-HC IV-27686			
Patient Stretcher/Trolley	MF	Ruhoko-HC IV-27715			
Patient Stretcher/Trolley	MF	Kabuyanda-HC IV-27901			
Patient Stretcher/Trolley	MF	Kazo-HC IV-27761	FAZZINI		
Phototherapy Unit	ME	Mbarara-RRH-838			6993

Equipment Name	Type	System ID	Model Name	Model No.	Serial No.
Phototherapy Unit	ME	Mbarara-RRH-895			20FTT0820411
Phototherapy Unit	ME	Mbarara-RRH-900			20FPT0820406
Phototherapy Unit	ME	Mbarara-RRH-902			7049
Phototherapy Unit	ME	Mbarara-RRH-903			
Phototherapy Unit	ME	Mbarara-RRH-916	EW200		20FPT05W0389
Phototherapy Unit	ME	Mbarara-RRH-957	EW200		20FPT05203911
Phototherapy Unit	ME	Mbarara-RRH-985			7046
Phototherapy Unit	ME	Mbarara-RRH-988			
Phototherapy Unit	ME	Mbarara-RRH-483	AS20L		46AIAZ01004
Phototherapy Unit	ME	Mbarara-RRH-485	AS20L		46AIAZ01002
Phototherapy Unit	ME	Mbarara-RRH-487	AS20L		B42BZZG01003
Phototherapy Unit	ME	Mbarara-RRH-530	BRILLIANCE PRO		7068
Phototherapy Unit	ME	Mbarara-RRH-531	BRILLIANCE PRO		7049
Phototherapy Unit	ME	Mbarara-RRH-532	BRILLIANCE PRO		7067
Phototherapy Unit	ME	Mbarara-RRH-533			
Phototherapy Unit	ME	Mbarara-RRH-543	BRILLIANCE PRO		6993
Phototherapy Unit	ME	Mbarara-RRH-544	BRILLIANCE PRO		7004
Phototherapy Unit	ME	Mbarara-RRH-552		AS20L	46AIAZ01004
Phototherapy Unit	ME	Mbarara-RRH-554		PT312	
Phototherapy Unit	ME	Mbarara-RRH-567	LULLABY		0086
Phototherapy Unit	ME	Mbarara-RRH-580			
Phototherapy Unit	ME	Mbarara-RRH-598		IREX20	20FPT0820411
Phototherapy	ME	Mbarara-	BRILLIANCE PRO		7058

Equipment Name	Type	System ID	Model Name	Model No.	Serial No.
Unit		RRH-610			
Phototherapy Unit	ME	Mbarara-RRH-613		IREX20	20FPT0820406
Phototherapy Unit	ME	Mbarara-RRH-617		AS20L	
Phototherapy Unit	ME	Mbarara-RRH-619		AS20L	
Phototherapy Unit	ME	Mbarara-RRH-810			7068
Phototherapy Unit	ME	Bwizibwera-HC IV-27537		A20	42130404022
Phototherapy Unit	ME	Kabuyanda-HC IV-27952		CFL101	7077
Power Stabilizers		Bushenyi-HC IV-29023	EPG 7500E2		phase
Power Stabilizers		Bushenyi-HC IV-29024			
Power Stabilizers		Ruhoko-HC IV-27670		5VC-5000VA	
Power Stabilizers		Kazo-HC IV-27740		SVC-5000VA	
Pulse Oximeter, Fingertip	ME	Kabuyanda-HC IV-27906		PM-60	CR-2B143385T
Pulse Oximeter, Fingertip	ME	Rugazi-HC IV-28922			
Pulse Oximeter, Fingertip	ME	Rugazi-HC IV-28923			
Pulse Oximeter, Fingertip	ME	Mbarara-RRH-1063	H100B		360101-M20314830142
Pulse Oximeter, Fingertip	ME	Mbarara-RRH-1064	H100B		
Pulse Oximeter, Fingertip	ME	Mbarara-RRH-1066	H100B		
Pulse Oximeter, Fingertip	ME	Mbarara-RRH-1068	H100B		
Pulse Oximeter, Fingertip	ME	Mbarara-RRH-1070	H100B		
Pulse Oximeter, Fingertip	ME	Mbarara-RRH-1071	H100B		
Pulse Oximeter, Fingertip	ME	Mbarara-RRH-1073	H100B		
Pulse Oximeter, Fingertip	ME	Mbarara-RRH-1075	H100B		
Pulse Oximeter, Fingertip	ME	Mbarara-RRH-1429			AH07080064
Refrigerator, Vaccine, Solar with battery	HSS	Mbarara-RRH-1050	KRYOSAFE		
Refrigerator, Vaccine, Solar with battery	HSS	Mbarara-RRH-1053	LG		3854JD1014F
Refrigerator,	HSS	Mbarara-	LG	LG	GC-5202SL

Equipment Name	Type	System ID	Model Name	Model No.	Serial No.
Vaccine, Solar with battery		RRH-1054			
Refrigerator, Vaccine, Solar with battery	HSS	Mbarara-RRH-1055	LG	GR-S392QVC	3850TZ-0043L
Refrigerator, Vaccine, Solar with battery	HSS	Mbarara-RRH-1057	DAYTEX	DTF-240	100403797
Refrigerator, Vaccine, Solar with battery	HSS	Mbarara-RRH-1059	DOMETIC	MLB3800CSG	7472189
Refrigerator, Vaccine, Solar with battery	HSS	Mbarara-RRH-1061	TAVER		
Refrigerator, Vaccine, Solar with battery	HSS	Mbarara-RRH-1062	TAVER	2001/2302	80440/46
Refrigerator, Vaccine, Solar with battery	HSS	Mbarara-RRH-1065	ZANUSSI	B25930	80900030
Refrigerator, Vaccine, Solar with battery	HSS	Mbarara-RRH-1067	PANASONIC	MPR-1014 PE	13050066
Refrigerator, Vaccine, Solar with battery	HSS	Mbarara-RRH-1069	THERMOSCIENTIFIC		
Refrigerator, Vaccine, Solar with battery	HSS	Mbarara-RRH-1072	GRAM	K210LG	862100461
Refrigerator, Vaccine, Solar with battery	HSS	Mbarara-RRH-1074	LABFREEZE		
Refrigerator, Vaccine, Solar with battery	HSS	Mbarara-RRH-1076	FREEZER	DWFL270	30002
Refrigerator, Vaccine, Solar with battery	HSS	Mbarara-RRH-1077	FREEZER	DWFL270	30002
Refrigerator, Vaccine, Solar with battery	HSS	Kyabugimbi-HC IV-29552		MF314	20031705269
Refrigerator, Vaccine, Solar with battery	HSS	Kyabugimbi-HC IV-30131			MEZ 64948601
Refrigerator, Vaccine, Solar with battery	HSS	Kyabugimbi-HC IV-30339			
Refrigerator, Vaccine, Solar with battery	HSS	Bushenyi-HC IV-29029	HXC-358		BE06NSEITOOQJK6H0009
Refrigerator, Vaccine, Solar with battery	HSS	Bushenyi-HC IV-29470	BRS-230		F286D0357
Refrigerator, Vaccine, Solar	HSS	Bushenyi-HC IV-29474	HXC106		BE02T0EOOOOQTN5J008

Equipment Name	Type	System ID	Model Name	Model No.	Serial No.
with battery					
Refrigerator, Vaccine, Solar with battery	HSS	Kabuyanda-HC IV-27900	Blood Bank		1771
Refrigerator, Vaccine, Solar with battery	HSS	Mbarara-RRH-516			
Refrigerator, Vaccine, Solar with battery	HSS	Mbarara-RRH-560		GR-222MVF	472018268
Refrigerator, Vaccine, Solar with battery	HSS	Mbarara-RRH-578		LR-100	11181080361
Refrigerator, Vaccine, Solar with battery	HSS	Bugamba-HC IV-32400	VLS300AC		20181708432
Refrigerator, Vaccine, Solar with battery	HSS	Bugamba-HC IV-32414		VC65F	1922-022
Refrigerator, Vaccine, Solar with battery	HSS	Kabwohe-HC IV-29799		VG50	946169028068300010
Refrigerator, Vaccine, Solar with battery	HSS	Kabwohe-HC IV-29870	RD-27DR		JB0205Z0276JBCS4BP10188
Refrigerator, Vaccine, Solar with battery	HSS	Kabwohe-HC IV-29950		F60HOI	0161133000801
Refrigerator, Vaccine, Solar with battery	HSS	Kabwohe-HC IV-29951			
Refrigerator, Vaccine, Solar with battery	HSS	Kabwohe-HC IV-29952			
Refrigerator, Pharmaceuticals	HSS	Mbarara-RRH-422		BLCRF 290W	02430007
Resuscitator, Manual, Adult	ME	Shuuku-HC IV-29490			
Space/Room Heaters	ME	Mbarara-RRH-386	MEDICAL ELECTRICAL	135	192-13514893
Space/Room Heaters	ME	Mbarara-RRH-553			
Spray Gun	HSS	Mbarara-RRH-2749	JAMBO	JS_20	TSWZZZ
Spray Gun	HSS	Mbarara-RRH-2750			
Spray Gun	HSS	Mbarara-RRH-2752			
Spray Gun	HSS	Mbarara-RRH-2753			
Spray Gun	HSS	Mbarara-RRH-2755			
Spray Gun	HSS	Mbarara-RRH-2756			

Equipment Name	Type	System ID	Model Name	Model No.	Serial No.
Spray Gun	HSS	Mbarara-RRH-2758			
Spray Gun	HSS	Mbarara-RRH-2760			
Spray Gun	HSS	Mbarara-RRH-2762			
Spray Gun	HSS	Mbarara-RRH-2765			
Spray Gun	HSS	Mbarara-RRH-2767			
Spray Gun	HSS	Mbarara-RRH-2768			
Spray Gun	HSS	Mbarara-RRH-2769			
Spray Unit, 15m	HSS	Mbarara-RRH-2770			
Steam Foaming Machine	HSS	Bushenyi-HC IV-29009	25X-2		0012589
Sterilizer Drum, Medium		Kyabugimbi-HC IV-29524			
Sterilizer Drum, Medium		Kyabugimbi-HC IV-30084			
Sterilizer Drum, Medium		Kyabugimbi-HC IV-30088			
Sterilizer Drum, Medium		Kyabugimbi-HC IV-30114			
Sterilizer Drum, Medium		Kyabugimbi-HC IV-30361			
Sterilizer Drum, Medium		Kyabugimbi-HC IV-30397			
Sterilizer Drum, Medium		Kyabugimbi-HC IV-30398			
Sterilizer Drum, Medium		Bushenyi-HC IV-29001			
Sterilizer Drum, Medium		Bushenyi-HC IV-29010			
Sterilizer Drum, Medium		Kabuyanda-HC IV-27868			
Sterilizer Drum, Medium		Kabuyanda-HC IV-27898			
Sterilizer Drum, Medium		Kabuyanda-HC IV-27925			
Sterilizer Drum, Medium		Rugazi-HC IV-28559			
Sterilizer Drum, Medium		Rugazi-HC IV-28560			
Sterilizer Drum, Medium		Rugazi-HC IV-28900			
Sterilizer Drum, Medium		Kabwohe-HC IV-29830			
Sterilizer Drum, Medium		Shuuku-HC IV-29491			
Sterilizer Drum,		Shuuku-HC			

Equipment Name	Type	System ID	Model Name	Model No.	Serial No.
Medium		IV-29751			
Sterilising Drum, Set (Small, Medium, Large)	HSS	Bugamba-HC IV-32326			
Sterilising Drum, Set (Small, Medium, Large)	HSS	Shuuku-HC IV-29492			
Sterilising Drum, Set (Small, Medium, Large)	HSS	Shuuku-HC IV-29752			
Sterilising Drum, Set (Small, Medium, Large)	HSS	Nyamuyanja-HC IV-28031			
Stethoscope	ME	Kyabugimbi-HC IV-30351			
Stethoscope	ME	Bushenyi-HC IV-29022			
Stethoscope	ME	Kabuyanda-HC IV-27904			
Stethoscope	ME	Kabwohe-HC IV-30073			
Stethoscope	ME	Shuuku-HC IV-30535			
Stethoscope nurses	ME	Kabuyanda-HC IV-27934			
Suction Apparatus, Electric	ME	Nyamuyanja-HC IV-27983	YX940D		Y63041
Suction Apparatus, Electric	ME	Nyamuyanja-HC IV-27988	ALSA		845PM-11/05
Suction Apparatus, Electric	ME	Mbarara-RRH-1408	SAM14	01010101	1012-2254
Suction Apparatus, Electric	ME	Mbarara-RRH-1410	SAM14	01010101	
Suction Apparatus, Electric	ME	Mbarara-RRH-1412	EUROVAC ELITE		7GELQ844
Suction Apparatus, Electric	ME	Mbarara-RRH-1414	SAM14	0410-0982	
Suction Apparatus, Electric	ME	Mbarara-RRH-1421	HERSILL		9760-47-039
Suction Apparatus, Electric	ME	Mbarara-RRH-464	SM-200D		00076
Suction Apparatus, Electric	ME	Mbarara-RRH-492	SM-200D		C518611-A
Suction Apparatus, Electric	ME	Mbarara-RRH-551	ASKIRC30		12019

Equipment Name	Type	System ID	Model Name	Model No.	Serial No.
Suction Apparatus, Electric	ME	Mbarara-RRH-569	ASKIRC30		1208
Suction Apparatus, Electric	ME	Mbarara-RRH-577	ASKIRC30		12013
Suction Apparatus, Electric	ME	Mbarara-RRH-581	ASKIRC30		12011
Suction Apparatus, Electric	ME	Mbarara-RRH-582		RE410250	12018
Suction Apparatus, Electric	ME	Mbarara-RRH-583	ASKIRC30		
Suction Apparatus, Foot	ME	Bugamba-HC IV-30428		F.170	090464
Suction Apparatus, Foot	ME	Rugazi-HC IV-28903			
Stool, Surgeon	MF	Bugamba-HC IV-30448			
Stool, Surgeon	MF	Bugamba-HC IV-30449			
Stool, Surgeon	MF	Bugamba-HC IV-30450			
Stool, Surgeon	MF	Bugamba-HC IV-30451			
Stool, Surgeon	MF	Rugazi-HC IV-28901	SW-D7-A		SW-D720220618
Stool, Surgeon	MF	Shuuku-HC IV-29518			
Stool, Surgeon	MF	Bwizibwera-HC IV-27546			
Stool, Surgeon	MF	Kyabugimbi-HC IV-30355			
Stool, Surgeon	MF	Kyabugimbi-HC IV-30356			
Stool, Surgeon	MF	Kyabugimbi-HC IV-30360			
Stool, Surgeon	MF	Kabuyanda-HC IV-27877			
Syringe Pump	ME	Mbarara-RRH-34103	AgiliaSP MC ZA	Z018691	25446480
Syringe Pump	ME	Mbarara-RRH-34114	Agilia SP MC ZA	Z018691	25446470
Syringe Pump	ME	Mbarara-RRH-34115	SAM 35	Z018691	255446476
Television Set		Ruhoko-HC IV-27725		21CD1RGE-TH	803GTBU00125
Television Set		Bwizibwera-HC IV-27511	32A5200F		3TE32W20500901CCGB00824
Television Set		Mbarara-RRH-896	LG		

Equipment Name	Type	System ID	Model Name	Model No.	Serial No.
Television Set		Mbarara-RRH-897	LG		
Television Set		Mbarara-RRH-898	LG		
Television Set		Mbarara-RRH-899	LG		
Timer	ME	Mbarara-RRH-1132	Brannan	Lot 2012/	C10Q65
Timer	ME	Mbarara-RRH-1133	Brannan	Lot 2012/	C10Q69
Timer	ME	Mbarara-RRH-1134	Assistent		
Timer	ME	Mbarara-RRH-1136	Assistent		
Timer	ME	Mbarara-RRH-1137	DIGITAL TIMER	VMR EU 609-0128	
Timer	ME	Mbarara-RRH-1139	DIGITAL TIMER	VMR EU 609-0129	
Timer	ME	Mbarara-RRH-1140	DIGITAL TIMER	VMR EU 609-0130	
Timer	ME	Mbarara-RRH-1142	DIGITAL TIMER	VMR EU 609-0131	111638512
Timer	ME	Kyabugimbi-HC IV-29546			
Timer	ME	Shuuku-HC IV-29510			
Tool Cabins		Kabuyanda-HC IV-27891			
Ultrasound Machine Colour Doppler	ME	Mbarara-RRH-47460	Ecube 8		L05150
Ultrasound Machine Colour Doppler	ME	Mbarara-RRH-1261	TYPE: GE	2019-07-24	603513WXO
Point of Care (POC) Handle Held Ultrasound	ME	Rugazi-HC IV-28541	SDIWE		04530004
Point of Care (POC) Handle Held Ultrasound	ME	Kyabugimbi-HC IV-30121	HD3-EXP-V2		A78202300003986
Ultrasound Scanner, Portable	ME	Mbarara-RRH-47461	EDAN	DUS 60	331034-M15800030006
Ultrasound Scanner, Portable	ME	Ishongororo-HC IV-27620	Sonodiagonostic 100E		70527013
Uninterruptible Power Supply (UPS)	HSS	Ishongororo-HC IV-27589		MST7501	351617x08337
Uninterruptible Power Supply (UPS)	HSS	Ishongororo-HC IV-27590		BVX700LUI-MS	9B2127A11635
Uninterruptible Power Supply	HSS	Ishongororo-HC IV-27591		BVX700LUI-MS	9B2127A11831

Equipment Name	Type	System ID	Model Name	Model No.	Serial No.
(UPS)					
Uninterruptible Power Supply (UPS)	HSS	Kazo-HC IV-27730			
Uninterruptible Power Supply (UPS)	HSS	Mbarara-RRH-31658	TRIPP-LITE	SU80KX	2130DLCP5680000003
Uninterruptible Power Supply (UPS)	HSS	Mbarara-RRH-31670		E3MUP560KHB	OE2207K41015
Uninterruptible Power Supply (UPS)	HSS	Mbarara-RRH-31672	E3MUES60KHE		OE2148K41022
Uninterruptible Power Supply (UPS)	HSS	Mbarara-RRH-901			9B2119A02495
Uninterruptible Power Supply (UPS)	HSS	Mbarara-RRH-958		BVX1600L1	9B2115A11435
Uninterruptible Power Supply (UPS)	HSS	Mbarara-RRH-959			
Uninterruptible Power Supply (UPS)	HSS	Mbarara-RRH-960			
Uninterruptible Power Supply (UPS)	HSS	Mbarara-RRH-961			
Uninterruptible Power Supply (UPS)	HSS	Mbarara-RRH-962			
Uninterruptible Power Supply (UPS)	HSS	Mbarara-RRH-1228	ACP	SMC30001	AS1718352062
Uninterruptible Power Supply (UPS)	HSS	Mbarara-RRH-1229	ACP	SUA10001	AS1232241757
Uninterruptible Power Supply (UPS)	HSS	Mbarara-RRH-1230	ACP	SUA10001	AS1232241746
Uninterruptible Power Supply (UPS)	HSS	Mbarara-RRH-1231	ONLINE	Zinto 800	1.70907E+11
Uninterruptible Power Supply (UPS)	HSS	Mbarara-RRH-1232	ACP	SmartC1000	3C1647X02607
Uninterruptible Power Supply (UPS)	HSS	Mbarara-RRH-1233	E-SERIES	NV	639700271
Uninterruptible Power Supply (UPS)	HSS	Bwizibwera-HC IV-27513	M108800		162490004762
Uninterruptible Power Supply (UPS)	HSS	Bwizibwera-HC IV-27514	L600242207511004		

Equipment Name	Type	System ID	Model Name	Model No.	Serial No.
Uninterruptible Power Supply (UPS)	HSS	Bwizibwera-HC IV-27517	BV6501-MSX		9B2112A09640
Uninterruptible Power Supply (UPS)	HSS	Bwizibwera-HC IV-27518	BV6501-MSX		9B2112A09637
Uninterruptible Power Supply (UPS)	HSS	Bwizibwera-HC IV-27527	BV6501-MSX		S/N: 9B2025A00129
Uninterruptible Power Supply (UPS)	HSS	Bwizibwera-HC IV-27529	BV6501-MSX		S/N:9B2017A23057
Uninterruptible Power Supply (UPS)	HSS	Bwizibwera-HC IV-27530	BV6501-MSX		SN:9B2025A00131
Uninterruptible Power Supply (UPS)	HSS	Mbarara-RRH-495	SUA3000		AS1042244482
Uninterruptible Power Supply (UPS)	HSS	Mbarara-RRH-499	SUA30001		AS2222576456
Uninterruptible Power Supply (UPS)	HSS	Mbarara-RRH-501	SUA 3000		AS1154576456
Uninterruptible Power Supply (UPS)	HSS	Mbarara-RRH-505		SUA 3000	AS1152245486
Uninterruptible Power Supply (UPS)	HSS	Mbarara-RRH-729	APC		9B2115A11434
Uninterruptible Power Supply (UPS)	HSS	Mbarara-RRH-786	APC	BVX1600L1	9B2115A11425
Uninterruptible Power Supply (UPS)	HSS	Mbarara-RRH-787	APC	BVX1600L1	9B2115A11425
Uninterruptible Power Supply (UPS)	HSS	Mbarara-RRH-788	APC	BVX1600L1	9B2115A11425
Uninterruptible Power Supply (UPS)	HSS	Mbarara-RRH-789	APC	BVX1600L1	9B2115A11425
Uninterruptible Power Supply (UPS)	HSS	Mbarara-RRH-790	APC	BVX1600L1	9B2115A11425
Uninterruptible Power Supply (UPS)	HSS	Mbarara-RRH-791	APC	BVX1600L1	9B2115A11425
Uninterruptible Power Supply (UPS)	HSS	Mbarara-RRH-792	APC	BVX1600L1	9B2115A11425
Uninterruptible Power Supply (UPS)	HSS	Mbarara-RRH-793	APC	BVX1600L1	9B2115A11425
Uninterruptible	HSS	Mbarara-	APC	BVX1600L1	9B2115A11425

Equipment Name	Type	System ID	Model Name	Model No.	Serial No.
Power Supply (UPS)		RRH-794			
Uninterruptible Power Supply (UPS)	HSS	Mbarara-RRH-795	APC	BVX1600L1	9B2115A11425
Uninterruptible Power Supply (UPS)	HSS	Mbarara-RRH-796	APC	BVX1600L1	9B2115A11425
Uninterruptible Power Supply (UPS)	HSS	Mbarara-RRH-797	APC	BVX1600L1	9B2115A11425
Uninterruptible Power Supply (UPS)	HSS	Mbarara-RRH-798	APC	BVX1600L1	9B2115A11425
Uninterruptible Power Supply (UPS)	HSS	Mbarara-RRH-799	APC	BVX1600L1	9B2115A11425
Uninterruptible Power Supply (UPS)	HSS	Mbarara-RRH-800	APC	BVX1600L1	9B2115A11425
Uninterruptible Power Supply (UPS)	HSS	Mbarara-RRH-801	APC	BVX1600L1	9B2115A11425
Uninterruptible Power Supply (UPS)	HSS	Mbarara-RRH-802	APC	BVX1600L1	9B2115A11425
Uninterruptible Power Supply (UPS)	HSS	Mbarara-RRH-803	APC	BVX1600L1	9B2115A11425
Uninterruptible Power Supply (UPS)	HSS	Mbarara-RRH-804	APC	BVX1600L1	9B2115A11425
Uninterruptible Power Supply (UPS)	HSS	Mbarara-RRH-805	APC	BVX1600L1	9B2115A11425
Vacuum Extractor, Manual	ME	Shuuku-HC IV-29488			
Vacuum Extractor, Manual	ME	Kyabugimbi-HC IV-30116			
Vacuum Pump	ME	Mbarara-RRH-992	BUSCH	RA 0160 D	C1624000259
VDRL Shaker	ME	Kazo-HC IV-27741	Stuart		R800008350
Vein Finder (Locater)	ME	Mbarara-RRH-1051	ZD-JM-260-04		210324Z104
Vein Finder (Locater)	ME	Mbarara-RRH-1052	ZD-JM-260-04		210326Z104
Ventilator with high frequency mode	ME	Mbarara-RRH-1264	CARE SCAPE	R860	CBRY02074
Ventilator with high frequency mode	ME	Mbarara-RRH-25257	Ventilation LS		35842

Equipment Name	Type	System ID	Model Name	Model No.	Serial No.
Ventilator with high frequency mode	ME	Mbarara-RRH-25260	Ventilation LS		35749
Ventilator with high frequency mode	ME	Mbarara-RRH-25262	Ventilation LS		35844
Ventilator with high frequency mode	ME	Mbarara-RRH-25264	Ventilation LS		35552
Ventilator with high frequency mode	ME	Mbarara-RRH-25266	WM120DT		99001723
Ventilator with high frequency mode	ME	Mbarara-RRH-25268	WM120DT		99002655
Ventilator with high frequency mode	ME	Mbarara-RRH-34097	Luft 5		G21460
Ventilator with high frequency mode	ME	Mbarara-RRH-34098	Luft5		G21457
Ventilator with high frequency mode	ME	Mbarara-RRH-34099	Luft5		G21456
Ventilator with high frequency mode	ME	Mbarara-RRH-384	ENGSTROM PRO		CBCRO2050
Ventilator with high frequency mode	ME	Mbarara-RRH-387	GRADIAN CCV		20200810015
Ventilator with high frequency mode	ME	Mbarara-RRH-388	GRADIAN CCV		201711170009
Ventilator, Adult, Mechanical	ME	Mbarara-RRH-353	ENGSTROM PRO		15059003000
Ventilator, Adult, Mechanical	ME	Mbarara-RRH-383	GRADIAN CCV		201712130006
Ventilator, Adult, Portable	ME	Mbarara-RRH-355	ENGSTROM PRO		99002855
Ventilator, Adult, Portable	ME	Mbarara-RRH-358		6882000	19947
Ventilator, Adult, Portable	ME	Mbarara-RRH-362	GRADIAN CCV		202010050030
Ventilator, Adult, Portable	ME	Mbarara-RRH-363			2020009030005
Ventilator, Adult, Portable	ME	Mbarara-RRH-378	GRADIAN CCV		201801230009
Ventilator, Adult, Portable	ME	Mbarara-RRH-31674	Left 5		G21457,G21460,G21456
Voltage Regulator		Mbarara-RRH-1418	STAC	SVR-1500W	
Vortex Mixer	ME	Mbarara-RRH-1041	DENILLE	NJ 08554	9110094
Vortex Mixer	ME	Mbarara-	STUART	SA8	R8400005769

Equipment Name	Type	System ID	Model Name	Model No.	Serial No.
		RRH-1043			
Vortex Mixer	ME	Kabuyanda-HC IV-27996		CL001	11068289
Waste Bin Trolley	HSS	Mbarara-RRH-392			8000
Water Bath	ME	Mbarara-RRH-1045	KOTTERMANN	3041	546161
Water Bath	ME	Mbarara-RRH-1047	GRANT	JB1	609639010
Water Bath	ME	Mbarara-RRH-1049	GFL	TYP 1013	10721413J
Water Distiller	ME	Mbarara-RRH-1112	GFL	2108	10346213J
Water Heater		Mbarara-RRH-753	ARISTON		
Weighing Scale, Adult with Height Meter	ME	Kyabugimbi-HC IV-29534			
Weighing Scale, Adult with Height Meter	ME	Ishongororo-HC IV-27588	KINLEE		
Weighing Scale, Adult with Height Meter	ME	Ruhoko-HC IV-27700	SECA		8354245150828
Weighing Scale, Adult with Height Meter	ME	Nyamuyanja-HC IV-27948	RGZ-160		
Weighing Scale, Adult with Height Meter	ME	Nyamuyanja-HC IV-27967			5762294165246
Weighing Scale with Height Meter (Paediatric)		Nyamuyanja-HC IV-27995	44LBSX20Z		
Weighing Scale with Height Meter (Paediatric)		Mbarara-RRH-494		57660	
Weighing Scale with Height Meter (Paediatric)		Mbarara-RRH-555		57660	C0566751
Weighing Scale with Height Meter (Paediatric)		Rugazi-HC IV-28554	M108800		16249-0004807
Weighing Scale, Adult	ME	Mbarara-RRH-34047	seca		2762276136452
Weighing Scale, Adult	ME	Mbarara-RRH-34048	seca		5762299165722
Weighing Scale, Adult	ME	Bugamba-HC IV-32412	RGZ-160		
Weighing Scale, Adult	ME	Rugazi-HC IV-28557	5758		07-2018

Equipment Name	Type	System ID	Model Name	Model No.	Serial No.
Weighing Scale, Adult	ME	Rugazi-HC IV-28902	S758		07-2018
Weighing Scale, Adult	ME	Kabwohe-HC IV-29866			
Weighing Scale, Adult	ME	Kabwohe-HC IV-29867			
Weighing Scale, Adult	ME	Kabwohe-HC IV-29888	EB2056		
Weighing Scale, Adult	ME	Kabwohe-HC IV-29953		ZT-160	
Weighing Scale, Adult	ME	Kabwohe-HC IV-30076			
Weighing Scale, Adult	ME	Kiruhura-HC IV-27836	DETECTO		
Weighing Scale, Adult	ME	Kiruhura-HC IV-27841	SALTER	180	
Weighing Scale, Adult	ME	Kiruhura-HC IV-27844	DETECTO		
Weighing Scale, Adult	ME	Kiruhura-HC IV-27845	SECA		57622991655694
Weighing Scale, Adult	ME	Bwizibwera-HC IV-27524	Sec93541		8354100151066
Weighing Scale, Adult	ME	Bwizibwera-HC IV-27542		S7660	
Weighing Scale, Adult	ME	Kyabugimbi-HC IV-29517		8741021658	5874061187108
Weighing Scale, Adult	ME	Kyabugimbi-HC IV-29544		7621019008	5762291164
Weighing Scale, Adult	ME	Kyabugimbi-HC IV-30107		874102658	5874061187118
Weighing Scale, Adult	ME	Kyabugimbi-HC IV-30371		8741021658	5874061187138
Weighing Scale, Adult	ME	Kyabugimbi-HC IV-30395		7661019004	2762152110107
Weighing Scale, Adult	ME	Ishongororo-HC IV-27571	KINLEE		
Weighing Scale, Adult	ME	Ishongororo-HC IV-27596	ELECTRO GENESIS		
Weighing Scale, Adult	ME	Ishongororo-HC IV-27601	KORONA		6128120
Weighing Scale, Adult	ME	Ishongororo-HC IV-27605		8741321009	5874057160788
Weighing Scale, Adult	ME	Ishongororo-HC IV-27606			
Weighing Scale, Adult	ME	Ishongororo-HC IV-27608	M320600-01		52834-0001538
Weighing Scale, Adult	ME	Ishongororo-HC IV-27609	DETECTO		
Weighing Scale, Adult	ME	Ishongororo-HC IV-27627			
Weighing Scale, Adult	ME	Ruhoko-HC IV-27717	KINLEE		
Weighing Scale,	ME	Ruhoko-HC			

Equipment Name	Type	System ID	Model Name	Model No.	Serial No.
Adult		IV-27719			
Weighing Scale, Adult	ME	Ruhoko-HC IV-27723	SECA		5874101189021
Weighing Scale, Adult	ME	Ruhoko-HC IV-27724	DETECTO		
Weighing Scale, Adult	ME	Nyamuyanja-HC IV-27953	874025658		5874100188674
Weighing Scale, Adult	ME	Nyamuyanja-HC IV-27955	7621019004		2762084137663
Weighing Scale, Adult	ME	Kabuyanda-HC IV-27855			
Weighing Scale, Adult	ME	Kabuyanda-HC IV-27965		8741021658	5784034184872
Weighing Scale, Adult	ME	Kabuyanda-HC IV-28014			
Weighing Scale, Adult	ME	Kazo-HC IV-27744			
Weighing Scale, Adult	ME	Kazo-HC IV-27751	SECA		
Weighing Scale, Adult	ME	Kiruhura-HC IV-27826	SECA		8354245151131
Weighing Scale, Infant, Beam Type	ME	Kyabugimbi-HC IV-30095			
Weighing Scale, Infant, Beam Type	ME	Nyamuyanja-HC IV-28027			57660
Weighing Scale, Infant, Beam Type	ME	Kabuyanda-HC IV-27924			
Weighing Scale, Infant, Beam Type	ME	Kabuyanda-HC IV-27972		M11260011	52411-0004393
Weighing Scale, Infant, Beam Type	ME	Bwizibwera-HC IV-27512			162490004762
Weighing Scale, Infant, Beam Type	ME	Mbarara-RRH-418		OT01	430044
Weighing Scale, Infant, Beam Type	ME	Mbarara-RRH-579		ACS-20B-YE	
Weighing Scale, Infant, Beam Type	ME	Mbarara-RRH-783			
Weighing Scale, Infant, Beam Type	ME	Mbarara-RRH-785			
Weighing Scale, Infant, Beam Type	ME	Mbarara-RRH-806			
Weighing Scale, Infant, Beam Type	ME	Mbarara-RRH-808	ASC-20B-YE		

Equipment Name	Type	System ID	Model Name	Model No.	Serial No.
Weighing Scale, Infant, Beam Type	ME	Mbarara-RRH-809	ACS-20B-YE		
Weighing Scale, Infant, Beam Type	ME	Bugamba-HC IV-30427		S7600	
Weighing Scale, Infant, Beam Type	ME	Bugamba-HC IV-30446			
Weighing Scale, Infant, Beam Type	ME	Bugamba-HC IV-30447		S7550	062018
Weighing Scale, Infant, Beam Type	ME	Bugamba-HC IV-32415		2356S	
Weighing Scale, Infant, Beam Type	ME	Kabwohe-HC IV-29803		354	35408502020398
Weighing Scale, Infant, Beam Type	ME	Shuuku-HC IV-29520	354		8354070100648
Weighing Scale, Paediatric	ME	Rugazi-HC IV-28908	TSEC		
Weighing Scale, Paediatric	ME	Shuuku-HC IV-29485	354		8354255150873
Weighing Scale, Paediatric	ME	Shuuku-HC IV-29486			
Wheel Chair	HSS	Mbarara-RRH-561			
Wheel Chair	HSS	Mbarara-RRH-562			
Wheel Chair	HSS	Mbarara-RRH-564			
Wheel Chair	HSS	Mbarara-RRH-566			
Wheel Chair	HSS	Ishongororo-HC IV-27604			
Wheel Chair	HSS	Ruhoko-HC IV-27712			1
Wheel Chair	HSS	Kazo-HC IV-27750			
Wheel Chair, Adult	HSS	Kyabugimbi-HC IV-30139			
Wheel Chair, Adult	HSS	Bushenyi-HC IV-29018			
Wheel Chair, Adult	HSS	Mbarara-RRH-31657			
Wheel Chair, Adult	HSS	Kabwohe-HC IV-30081			
Workstation Computer	HSS	Mbarara-RRH-1267		D19m	Vv7D401
Worktable, Stainless Steel	MF	Mbarara-RRH-31667			

Equipment Name	Type	System ID	Model Name	Model No.	Serial No.
X-ray Film Drier, Electric		Mbarara-RRH-47457			
X-ray Film Viewer	ME	Mbarara-RRH-399		NBX1I	0110/0257
X-ray Film Viewer	ME	Mbarara-RRH-401		NBX1I	0110/0244
X-ray Film Viewer	ME	Mbarara-RRH-409		NBX1I	0110/0254
X-ray Film Viewer	ME	Mbarara-RRH-410		NBX1I	0110/0249
X-ray Film Viewer	ME	Mbarara-RRH-472		NBX1I	0110/0259
X-ray Film Viewer	ME	Mbarara-RRH-475		NBX1I	0110/0260
X-ray Film Viewer	ME	Mbarara-RRH-513			
X-Ray Film Viewer, Portable	ME	Mbarara-RRH-371	NBX11		0110/0262
X-Ray Film Viewer, Portable	ME	Mbarara-RRH-372	NBX11		0110/0261
X-Ray Film Viewer, Portable	ME	Mbarara-RRH-377			0110/0262
X-Ray Machine, Mobile, Digital	ME	Mbarara-RRH-47454	Siemens	7742450	6639
